# Transgenic Expression of the Anti-parasitic Factor TEP1 in the Malaria Mosquito *Anopheles gambiae*

**DOI:** 10.1371/journal.ppat.1006113

**Published:** 2017-01-17

**Authors:** Gloria Volohonsky, Ann-Katrin Hopp, Mélanie Saenger, Julien Soichot, Heidi Scholze, Jens Boch, Stéphanie A. Blandin, Eric Marois

**Affiliations:** 1 Université de Strasbourg, CNRS UPR9022, INSERM U963, Institut de Biologie Moléculaire et Cellulaire, Strasbourg, France; 2 Martin-Luther Universität Halle-Wittenberg, Institut für Genetik, Halle (Saale), Germany; Institut Pasteur, FRANCE

## Abstract

Mosquitoes genetically engineered to be resistant to *Plasmodium* parasites represent a promising novel approach in the fight against malaria. The insect immune system itself is a source of anti-parasitic genes potentially exploitable for transgenic designs. The *Anopheles gambiae* thioester containing protein 1 (TEP1) is a potent anti-parasitic protein. TEP1 is secreted and circulates in the mosquito hemolymph, where its activated cleaved form binds and eliminates malaria parasites. Here we investigated whether TEP1 can be used to create malaria resistant mosquitoes. Using a GFP reporter transgene, we determined that the fat body is the main site of *TEP1* expression. We generated transgenic mosquitoes that express TEP1r, a potent refractory allele of *TEP1*, in the fat body and examined the activity of the transgenic protein in wild-type or *TEP1* mutant genetic backgrounds. Transgenic TEP1r rescued loss-of-function mutations, but did not increase parasite resistance in the presence of a wild-type susceptible allele. Consistent with previous reports, TEP1 protein expressed from the transgene in the fat body was taken up by hemocytes upon a challenge with injected bacteria. Furthermore, although maturation of transgenic TEP1 into the cleaved form was impaired in one of the *TEP1* mutant lines, it was still sufficient to reduce parasite numbers and induce parasite melanization. We also report here the first use of Transcription Activator Like Effectors (TALEs) in *Anopheles gambiae* to stimulate expression of endogenous *TEP1*. We found that artificial elevation of *TEP1* expression remains moderate *in vivo* and that enhancement of endogenous *TEP1* expression did not result in increased resistance to *Plasmodium*. Taken together, our results reveal the difficulty of artificially influencing TEP1-mediated *Plasmodium* resistance, and contribute to further our understanding of the molecular mechanisms underlying mosquito resistance to *Plasmodium* parasites.

## Author Summary

We examined whether the natural anti-parasitic protein TEP1 can be harnessed to generate malaria resistant mosquitoes. We report the expression pattern of genes of the *TEP1* immune pathway and the effects of both exogenous and enhanced endogenous expression of *TEP1* on the development of *Plasmodium* in *Anopheles gambiae*. We found that exogenous expression of *TEP1* increases resistance to *Plasmodium* in *TEP1* mutants but not in a wild type background, and that enhancing expression of endogenous *TEP1* does not increase resistance. We conclude that increased expression of *TEP1* alone is not sufficient to render mosquitoes resistant to *Plasmodium*.

## Introduction

Malaria is a devastating disease annually infecting over 200 million people worldwide, and is a leading cause of death in Sub-Saharan Africa [1]. The malaria-causing *Plasmodium* parasites are vectored by Anopheline mosquitoes, which constitute the obligatory primary host for *Plasmodium*. Out of 462 described species of *Anopheles* mosquitoes, about 34 are dominant vectors of human malaria [2,3]. *A*. *gambiae* is the major vector of the most deadly parasite, *P*. *falciparum*, in Sub-Saharan Africa. Thanks to vector control and the availability of anti-malarial combination therapies, estimated global malaria cases and mortality rates between 2000 and 2015 have declined by 37% and 60%, respectively, falling to approximately 440,000 deaths annually. Despite this downward trend, the fight against malaria is complicated by the spread of genetic resistance to insecticides in mosquitoes, and to anti-malarial drugs in *Plasmodium*. To prevent a reversal in the current malaria decline, new vector control strategies need to be developed.

Vector control strategies based on genetically modified mosquitoes have been advocated for over 15 years to complement existing anti-malaria interventions (reviewed by [4] and [5]). Interventions based on the sterile insect technique (SIT) [6,7] or its transgenic variants, such as the Release of Insects carrying a Dominant Lethal (RIDL) technique deployed in recent years against the dengue vector mosquito *Aedes aegypti* [8,9,10], are unpractical on the vast geographical scales of malaria transmission. Gene drive-based strategies, whereby a desired genetic character (female sterility for population suppression, malaria resistance for population replacement) is inserted on a selfish genetic element designed to spread throughout a target population, have raised great interest [11,12,13] but their development has been limited by the paucity of molecular engineering tools. Today, these prospects are regaining momentum thanks to the advent of the CRISPR-Cas9 system, which provides a simple means to build a gene drive construct [14,15]. Major leaps towards establishing CRISPR-Cas9 gene drive designs for spreading malaria resistance and for population suppression have been accomplished in two recent studies [16,17], moving these approaches a step closer to implementation. However, the capacity to modify, or even eradicate, an entire insect species poses serious ethical and environmental questions. While regional population suppression or eradication appears desirable for invasive insect species such as *Aedes aegypti* that is not part of the native ecosystem in most of its current range, the importance of the role that a “legitimate” species like *A*. *gambiae* in Sub-Saharan Africa plays in its ecosystem needs to be examined. In this case, gene drive strategies aiming at population replacement with malaria-refractory mosquitoes rather than population suppression may be preferred, at least as a first test of gene drive interventions within a malaria eradication program. Therefore, candidate genes that would confer mosquito resistance against malaria parasites need to be screened and validated for efficiency in transgenic mosquitoes.

When *Plasmodium* ookinetes, the motile stage of the parasite that forms in the blood meal of female mosquitoes, traverse the mosquito midgut epithelium and reach the basal lamina facing the insect’s hemocoele, they are attacked by the mosquito’s innate immune system. This process has been mostly studied in *A*. *gambiae* mosquitoes in combination with the murine parasite *P*. *berghei*, which offers a convenient model to study *Anopheles*—*Plasmodium* interactions in the laboratory [18]. The killing of ookinetes at the basal lamina is associated with direct binding of mosquito Thioester protein 1 (TEP1) to the ookinete surface [19]. TEP1 is a homolog of the mammalian C3 complement factor. Like C3, which circulates in mammalian blood, TEP1 is a soluble hemolymph protein and its activity is dependent on an intact thioester bond thought to mediate covalent binding to bacteria and parasites [19,20]. TEP1 is secreted as a full-length protein (TEP1-full) and cleaved to produce activated TEP1 (TEP1-cut) in which the two cleaved parts of TEP1 remain connected by a disulfide bond [20,21,22]. TEP1-cut circulates in the hemolymph as a complex with two leucine rich repeat (LRR) proteins, LRIM1 and APL1C. Knocking down *TEP1* itself or any of these LRR genes results in the disappearance of TEP1-cut from the hemolymph, and eliminates parasite killing [21,22]. However, TEP1 does not appear to directly lyse pathogens and the precise mechanism of killing remains unknown.

TEP1 has also been shown to act in mosquito resistance against the human parasite *P*. *falciparum*, though parasite virulence factors such as the ookinete surface protein Pfs47 have evolved in some parasite strains to counteract the TEP1-dependent immune response [23,24]. In addition, several *TEP1* alleles are present in mosquito populations. The abundant TEP1*S alleles (hereinafter termed *TEP1s*) are associated with susceptibility to *P*. *berghei* infection, supporting the production of large numbers of oocysts. The less frequent TEP1*R1 allele (hereinafter *TEP1r*) confers high resistance to *P*. *berghei*, and leads to parasite melanization [25]. Consistently, the laboratory L3-5 line that was selected based on the ability to kill *P*. *cynomolgi* [26] also kills all *P*. *berghei* parasites, is homozygous for *TEP1r* and exhibits high levels of parasite melanization. Furthermore, silencing *TEP1* in mosquitoes before infection further increases parasite loads in susceptible mosquitoes and abolishes resistance and parasite melanization in refractory mosquitoes [19]. Recently obtained TEP1 mutants exhibit the same phenotype [27].

Melanization is a powerful anti-parasitic and anti-microbial defense response used by insects to isolate and kill invading pathogens. The biochemical process has been well characterized in *Drosophila* and shown to be tightly regulated by both positive and negative effectors [28]. Recognition of pathogens triggers activation of a serine protease cascade that culminates in the cleavage of the inactive circulating Prophenoloxidase (PPO) to its active Phenoloxidase (PO) form. PO then oxidizes quinones that polymerize to form melanin, which is in turn cross-linked with proteins and deposited on the pathogen. This chain reaction is localized in close proximity to the pathogen. High local levels of reactive oxygen species arising from quinone oxidation reactions also contribute to pathogen killing [29]. In mosquitoes, this serine protease cascade is negatively regulated by serpins and activated by several CLIP domain proteases [30,31]. In resistant mosquitoes, silencing either *TEP1*, *LRIM1* or *APL1C* abolishes melanization and allows parasite survival [19,21,22,32]. Furthermore, it is possible to disrupt melanization by knocking down genes that regulate actin polymerization without affecting the killing of parasites [33]. These results suggest that the role of *TEP1r* in the resistant L3-5 mosquitoes is first in killing the parasites, then in promoting their melanization.

Here, we investigated the possibility of rendering mosquitoes resistant to *P*. *berghei* by transgenic expression of *TEP1r*. Together with insights regarding the potential of *TEP1* transgenes as malaria control agents, our observations reveal new aspects of TEP1 biology, including expression pattern, promoter characterization, competition between *TEP1* allelic forms, and anti-parasitic activity.

## Results

### The *TEP1*, *LRIM1* and *APL1C* expression pattern in transgenic reporter mosquitoes

**Fig 1:**
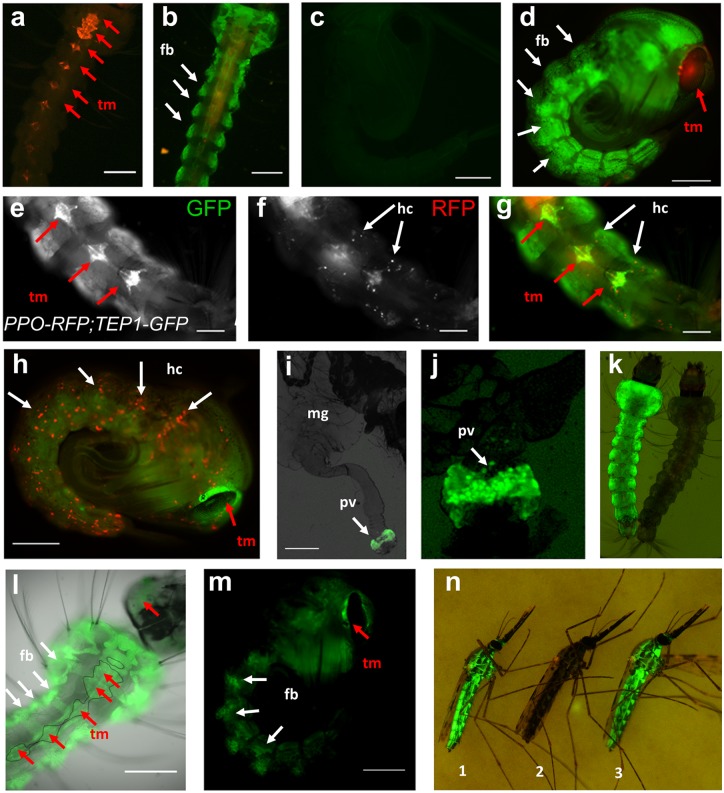
Expression patterns of transgenic reporter lines. The *3xP3* promoter drives expression of transgenesis markers in eyes and nerve cells. **a**. Control *3xP3-RFP* transgenic *A*. *gambiae* larva, image is a merge of green and red channels. Red arrows point to RFP expression in nerve cells, used as transgenesis marker (tm). **b**. *TEP1-GFP*, *3xP3-RFP* larva. Arrows point to some of the lobes of the fat body expressing GFP. Note the fine architecture of thoracic fat body revealed by GFP expression. **c**. control wild type pupa, showing little auto fluorescence in the green channel. **d**. *TEP1-GFP*, *3xP3-RFP* pupa showing GFP expression in fat body (fb, white arrows) and RFP expression in the eye (red arrows, tm). **e-g.** Larva carrying both *ppo6-RFP*, *3xP3-YFP* and *TEP1-GFP*, *3xP3-RFP*. RFP is expressed in hemocytes (white arrows) while GFP is expressed in fat body cells. YFP and RFP transgenic markers (red arrows, tm) are expressed in the nervous system. Scale bar = 200 μm **h.** RFP and YFP expression in *ppo6-RFP*, *3xP3-YFP* pupa, showing RFP expression in hemocytes (hc). White arrows point to some RFP expressing hemocytes. Transgenesis YFP marker is expressed in the eye (red arrow, tm). **i**. Midgut of *TEP1-GFP*,*3xP3-RFP* female 24h after blood feeding, showing distinct GFP expression in proventriculus. (pv) **j**. Close-up on a proventriculus expressing GFP in TEP1-GFP gut. **k**. *LRIM1-GFP*,*OpIE2-pac* larva showing GFP expression in the fat body, alongside a control (wt) larva. **l**. Larva expressing *APL1C-GFP*, *3xP3-CFP* showing GFP expression in the fat body (white arrows) and CFP transgenesis marker expression (red arrows, tm) in the nervous system (delineated with a thin line) **m**. *APL1C-GFP*,*3xP3-CFP* pupa expressing GFP in the fat body (white arrows) and CFP transgenesis marker in the eyes (tm, red arrow). **n.** *TEP1-GFP* (1) and *APL1C-GFP* (3) adult females with intense reporter expression in the fat body, visible through the cuticle, alongside a control female (2). Images **a**-**h**, **j**-**m** were acquired on a Zeiss Axiovert 200M microscope using a 5x or 10x objective. Images **i** and **n** were acquired on a Nikon SMZ18 binocular fluorescence microscope. Scale bar = 500μm except for **d**-**h**, **l**-**m**: 200 μm.

**Fig 2:**
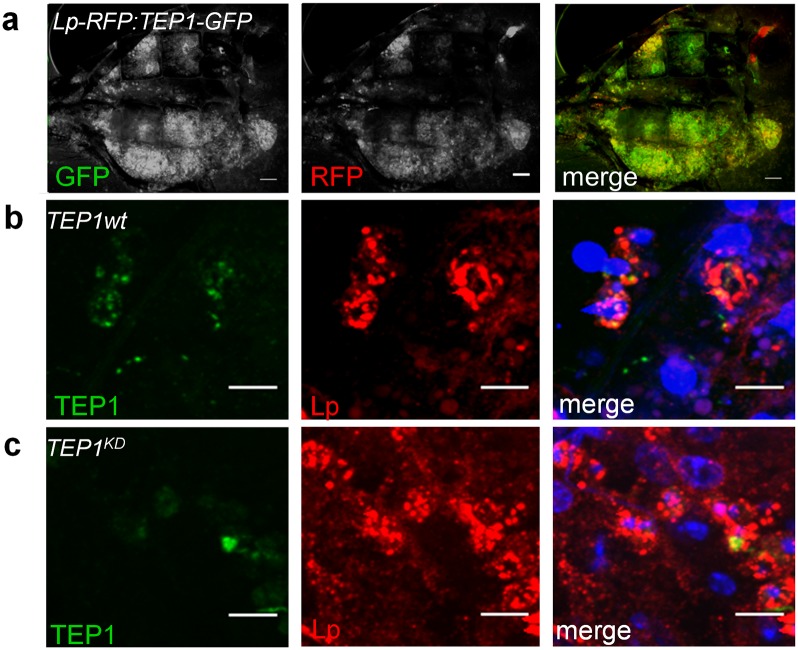
TEP1 expression in fat body cells. **a.** Dissected carcass of *TEP1-GFP*,*3xP3-RFP* and *Lp-RFP*, *3xP3-CFP* mosquito exposing the fat body that shows GFP and RFP expression. Merged image shows co-expression of *Lp* and *TEP1* reporters in the fat body. Scale bar = 200 μm. **b-c.** Stained carcasses of wild type (**b**) and mosquitoes treated with dsTEP1 (*TEP1KD* (**c**)) showing TEP1 antibody staining (green) in Lp-expressing fat body cells. Note reduction in TEP1 staining in *TEP1KD* carcasses. Dapi stained cell nuclei (blue). Shown are z-projections of confocal images, scale bar = 10μm. Images were collected on a Zeiss LSM 710 confocal microscope.

In order to over-express *TEP1* in mosquitoes in the endogenous source tissue, we first sought to determine which cells express *TEP1* mRNA and attempted to perform RNA *in situ* hybridization in both larva and adult mosquitoes. However, the fat body tissue did not withstand the procedure and was lost from the analysis. Thus, we constructed a transgenic reporter line expressing *GFP* under control of the *TEP1* promoter. For this, we cloned a 3.1 kb genomic DNA fragment located 5’ of the *TEP1s* gene from the *A*. *gambiae* G3 strain, ending at the *TEP1s* initiator ATG codon. Transfection assays in *Drosophila* S2 cells confirmed that this cloned *TEP1*s 5’ region is responsive to bacterial challenge (see below), as is the endogenous *TEP1* gene [20]. Investigation of the expression pattern of *GFP* in the transgenic mosquitoes revealed strong expression in the fat body tissue at larval (Fig 1a and 1b), pupal (Fig 1c and 1d) and adult stages (Figs 1n and 2). In addition, intense *GFP* expression was detected in cells of the midgut proventriculus (cardia) of adult mosquitoes, both males and females, regardless of blood feeding (Fig 1i and 1j, and S1a and S1b Fig). Other tissues showing some GFP expression included the oviduct, and more variably, the Malpighian tubules (S1c Fig). GFP was expressed in fat body loosely attached to the testis, but was not observed in the male reproductive organs themselves.

As a control for fat body and hemocyte expression we used the *Lipophorin* (*Lp*) promoter-driven RFP reporter line constitutively expressing RFP in the fat body and the hemocyte-specific *PP06-RFP* line [34]. Comparing the expression of *TEP1-GFP* to the *Lp-RFP* pattern shows similar expression in the same cells in dissected carcass tissues (Fig 2a). Mosquitoes carrying both reporter genes showed perfect GFP/RFP overlap. Upon crossing *TEP1-GFP* mosquitoes to the hemocyte reporter *PPO6-RFP* line, the observed RFP-positive hemocytes were never positive for GFP, indicating that the *TEP1* reporter is not strongly expressed in the hemocytes that express *PPO6* (Fig 1e–1h). Although the reporter mosquitoes did not display obvious GFP-positive hemocytes, we do not rule out a possible expression of endogenous *TEP1* in some hemocytes, as the cloned regulatory sequences may lack distant hemocyte-specific enhancers.

To identify the expression pattern of other genes known to act in the *TEP1* pathway, we also constructed transgenic reporter lines expressing *GFP* under the control of the promoters of the leucine-rich proteins LRIM1 and APL1C that associate with TEP1 in the hemolymph. Both reporter lines showed a *GFP* expression pattern similar to *TEP1*, namely expression in the fat body at all developmental stages (Fig 1k–1n). However, neither *LRIM1-GFP* nor *APL1C-GFP* exhibited *GFP* expression in the proventriculus. Of note, all these reporters were created using the same docking line, and are thus subjected to the same potential positional effects. Many other fluorescent reporter constructs inserted at the same locus exhibit different, promoter-specific patterns of expression [34], indicating that the *TEP1*, *LRIM1* and *APL1C* promoters must contain sequences determining fat body-specific expression.

In mosquitoes, fat body cells are challenging to manipulate. Dissection of mosquitoes in buffer results in immediate loss of large parts of the fat body. The fat body cells that remain attached to the carcass detach easily during fixation and disappear during permeabilization in buffers containing detergents. To verify the expression of TEP1 in the fat body cells that remain attached to dissected mosquito carcasses, we performed an antibody staining of fixed samples using a modified protocol (see materials and methods). Indeed, careful examination showed that some fat body cells that express the fat body specific protein Lipophorin (Lp) also stain positive for TEP1 (Fig 2b). In mosquitoes where *TEP1* was silenced by RNAi, this fat body TEP1 staining was markedly reduced (Fig 2c), indicating that the signal is TEP1-specific.

### *TEP1-GFP* reporter expression varies greatly among individual mosquitoes

Larvae and adults of the transgenic *TEP1-GFP* reporter line express *GFP* at strikingly variable levels (S1d and S1e Fig). We followed the levels of GFP during larval development (S2 Fig) and found an increase in GFP levels in time, most fourth instar larvae having reached a maximum level of GFP expression, though striking differences from larva to larva persist until pupation. Newly eclosed adult mosquitoes also exhibit variable levels of GFP expression (S1e Fig), probably reflecting a high variability in *TEP1* expression that contrasts with the highly reproducible expression level of the *Lp-RFP* reporter. We hypothesized that the variability in GFP expression reflected TEP1 induction by immune pathways [35] upon encounter with various microorganisms, which may differ from larva to larva. We tested whether there is a correlation between the level of GFP in adult mosquitoes and intensity of *P*. *berghei* infection. Adult mosquitoes were scored for GFP expression prior to infectious blood feeding and grouped according to weak or strong reporter expression level. Mosquitoes were infected and oocysts were counted 7 days post infection. S1 Table summarizes the result of 5 independent experiments, showing no significant differences in oocysts loads between the high and low GFP expressing mosquitoes in 4 out of 5 experiments.

### Transgenic expression of *TEP1* in the fat body

Given the localization of *GFP* expression under control of the *TEP1* promoter, we decided to express the *TEP1* transgene in adult fat body tissue. To avoid unnaturally high TEP1 expression throughout development, we used the blood-meal inducible *Vg* promoter. *Vg* is induced at high levels after blood feeding [36] peaking 24h after blood meal, at the time when *P*. *berghei* ookinetes reach the basal lamina and encounter circulating TEP1. This experimental design aimed at achieving high levels of TEP1 simultaneously to *Plasmodium* infection.

Since the *TEP1r* allele is associated with refractoriness to *P*. *berghei* infection and shown to be more potent in *Plasmodium* killing, we based our construct on the *TEP1r* allele. *TEP1r* cDNA was cloned under the *Vg* promoter and injected into the *TEP1s*-containing X1 docking line [34], to create the *Vg-TEP1r*,*3xP3-RFP* transgenic line (henceforth referred to as *Vg-TEP1r)*. Analysis of *TEP1r* mRNA in the transgenic mosquitoes (S3a Fig) showed blood meal induction of *TEP1r* similar to induction of *Vg* [37]. Like *Vg* mRNA, *TEP1r* mRNA levels monitored with *TEP1r*-specific primers peaked 24h after blood meal and dropped gradually in the following days. However, Western blot analysis of the transgenically expressed TEP1r protein in the *TEP1s* background did not reveal a notable increase in TEP1 protein levels in the transgenic mosquitoes 24h after blood feeding (S3b Fig), suggesting tight post-transcriptional control of hemolymph TEP1 protein levels.

**Fig 3:**
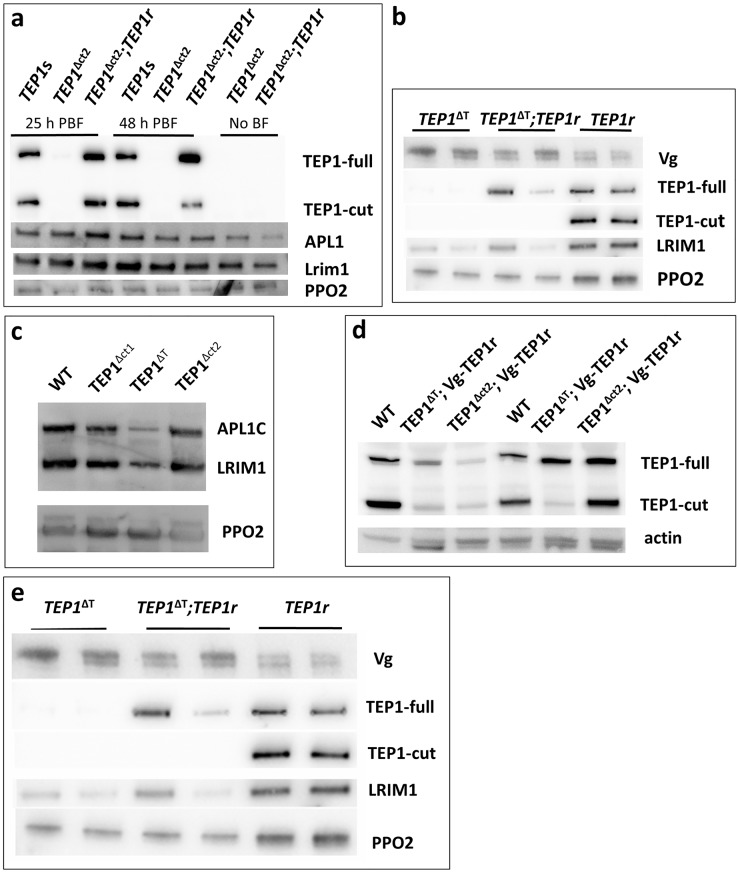
Transgenic expression of *TEP1r* under the *Vg* promoter. **a**. Western blot analysis of hemolymph from *Plasmodium* infected wild type (*TEP1s*), mutant (*TEP1*^*Δct2*^) and mutant mosquitoes ectopically expressing *TEP1r* (*TEP1*^*Δct2*^*;TEP1r;*) showing full (TEP1-full) and cleaved (TEP1-cut) TEP1 forms, LRIM1 and APL1C protein, 25 and 48h after infectious blood feeding. PPO2 is used as a loading control **b**. Western blot analysis of hemolymph from *Plasmodium* infected mosquitoes of the *TEP1*^*ΔT*^ mutant background (*TEP1*^*ΔT*^), ectopically expressing *TEP1r* (*TEP1*^*ΔT*^*; TEP1r*) and wild type L3-5 mosquitoes expressing only the endogenous *TEP1r* allele (*TEP1r*). 3–4 day old mosquitoes were blood fed to induce the *Vg* promoter. Samples were collected 24h after blood meal. The membrane was probed with the indicated antibodies, including anti-Vg to confirm proper induction of the *Vg* promoter. Two independent samples were loaded for each condition to account for variability. **c.** Western blot analysis of hemolymph from control and *TEP1* mutant mosquitoes showing the levels of APL1C and LRIM1. PPO2 is used as a loading control. Note the specific reduction of the two LRR proteins only in *TEP1ΔT* mosquitoes in which TEP1 lacks a single threonin. *TEP1Δct1* and *TEP1Δct2* are two different mutants lacking the entire C-terminus [27]. **d.** Western blot analysis of carcasses from mosquitoes 48h after blood feeding showing the levels of TEP1-full and -cut in the indicated genotypes. Two independent samples of each genotype are shown to represent variability. Actin (upper band of the doublet) used as loading control. **e**. Western blot analysis of hemolymph from mosquitoes injected with heat-killed *E*. *coli* 24h after blood feeding comparing TEP1 levels in injected and non-injected mosquitoes of the indicated genotypes. Note the absence of transgenic TEP1r cleavage and reduced LRIM1 in the *TEP1ΔT* mutation background regardless of bacterial injection. *PPO2* was used as a loading control.

In order to verify expression from the *Vg-TEP1r* transgene, we introduced it into two different *TEP1* null mutant lines [27]. The *TEP1Δct2* mutant has a nonsense mutation causing a premature stop codon and loss of the entire C-terminus. The *TEP1ΔT* mutant has a deletion of a threonine located 16 amino acids before the thioester cysteine. Both mutants lack detectable TEP1 protein in the hemolymph at 24h after blood feeding, but low levels of mutated TEP1 become detectable in old *TEP1ΔT* mosquitoes only. Samples from *TEP1Δct2; Vg-TEP1r* mosquitoes analyzed by polyclonal anti TEP1 antibodies showed the presence of both forms of TEP1, namely TEP1-full and TEP1-cut in the hemolymph expressed from the transgene (Fig 3a). In contrast, hemolymph samples from *TEP1ΔT; Vg-TEP1r* showed only the TEP1-full form (Fig 3b), suggesting that traces of the mutant TEP1ΔT protein inhibit maturation of wild-type TEP1 expressed from the transgene. In addition, *TEP1ΔT* (but not *TEP1Δct2*) mosquitoes show reduced levels of LRIM1 and APL1C (Fig 3a–3c and 3e). Unlike LRIM1 and APL1C depletion by RNAi silencing [21], this decrease does not result in massive TEP1-cut deposition on tissue (Fig 3d). Thus, traces of the mutant TEP1ΔT protein appear to downregulate both the endogenous LRIM1/APL1C complex and the cleavage of transgenic TEP1-full.

It has been previously demonstrated that injection of bacteria into mosquitoes promotes TEP1 maturation and activates *TEP1* transcription [20]. Furthermore, shortly after bacteria injection, TEP1-cut binds to bacterial surfaces and acts as a convertase to recruit the full-length protein [38,39]. The recruited TEP1-full is then cleaved close to bacterial surfaces and increases bacterial opsonization, thus marking bacteria for phagocytosis by hemocytes [40]. We tested whether injection of bacteria induced formation of the TEP1-cut form in the *TEP1ΔT;Vg-TEP1r* mosquitoes (Fig 3e). While the positive control *TEP1r* mosquitoes showed an increase in both TEP1-full and TEP1-cut after bacterial injection, transgenic TEP1-full in the *TEP1ΔT;Vg-TEP1r* mosquitoes’ hemolymph still failed to be converted to TEP1-cut.

### Infection of TEP1r transgenic mosquitoes with *P*. *berghei*

In order to verify that the *TEP1r* transgene was functional, we infected *TEP1* mutant mosquitoes complemented with the *Vg-TEP1r* transgene. The *TEP1Δct2;Vg-TEP1r* mosquitoes resemble the L3-5 line in that they express only the *TEP1r* allele. However, an important difference from the L3-5 line is that *TEP1r* is expressed exclusively in the fat body and only after a blood meal. In the *TEP1ΔT;Vg-TEP1r* line, only the full form of TEP1 is detectable in the hemolymph, which offers an opportunity to examine the activity of the TEP1-full form in the absence of detectable TEP1-cut.

**Fig 4:**
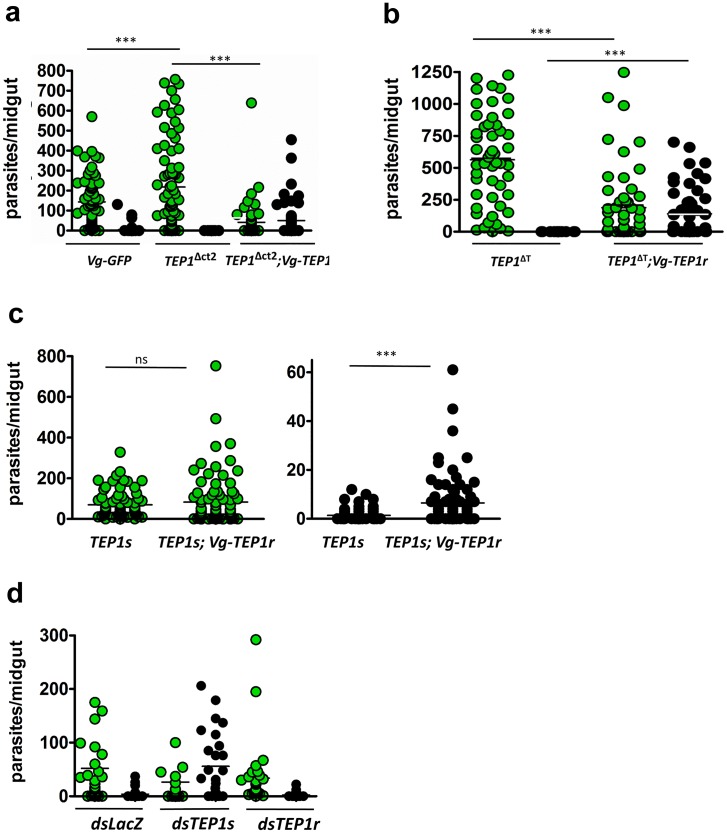
*P*. *berghei* infections of mosquitoes expressing *TEP1r* under the *Vg* promoter. **a**. *P*. *berghei* infection of *TEP1*^*Δct2*^ mutant mosquitoes rescued with transgenic *TEP1*r. Shown are the number of live parasites (green circles) and melanized parasites (black circles) in the midguts of mosquitoes 7 days after infection. Control mosquitoes are *TEP1* mutant alone (*TEP1*^*Δct2*^) and transgenic mosquitoes expressing *GFP* instead of *TEP1r* (*Vg-GFP*). **b**. *P*. *berghei* infection of *TEP1*^*ΔT*^ mutant mosquitoes compared to *TEP1*^*ΔT*^ rescued with transgenic *TEP1*r (*TEP1*^*ΔT*^*;Vg-TEP1r)*. **c.** *P*. *berghei* infection of transgenic mosquitoes expressing *TEP1r* in a wild type *TEP1s* background. Live (green circles) and melanized (black circles) parasites are compared in wild type (*TEP1s*) and transgenic (*TEP1s;Vg-TEP1r*) mosquitoes. **d**. *P*. *berghei* infection of transgenic *TEP1r* mosquitoes in wild type *TEP1s* background after *TEP1* allele-specific knockdown. Compared are transgenic mosquitoes treated with *dsLacZ*, *dsTEP1s* and *dsTEP1r*, 3 days prior to infection. For all *P*. *berghei* infections, experiments were performed at least 3 times, representative experiments are shown. The raw numbers of parasites and calculated *p values* for all infections are provided in S2 Table.

**Fig 5:**
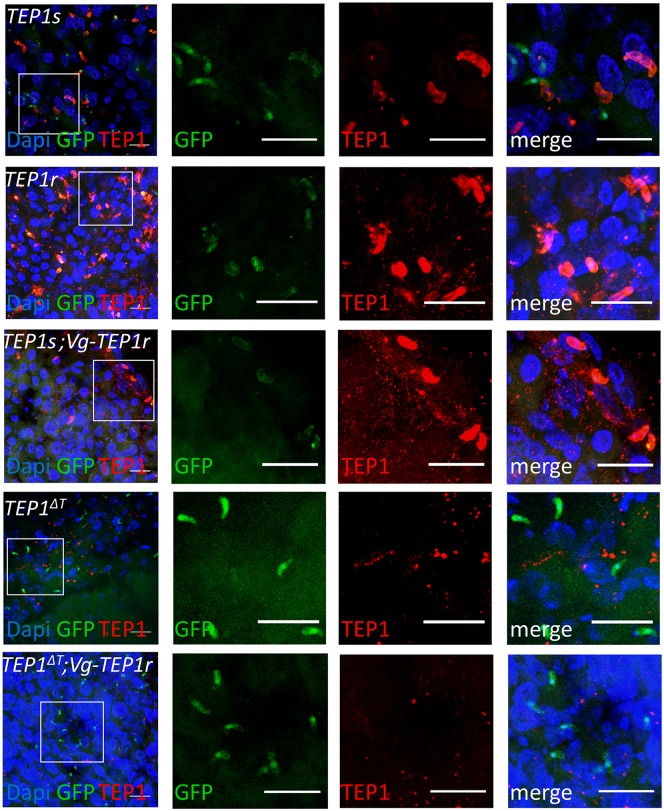
Binding of transgenically expressed TEP1 to ookinetes. TEP1 antibody staining (red) of dissected midguts 24 h after infection with GFP-expressing *P*. *berghei*. Shown are wild type mosquitoes expressing the *TEP1s* allele *(TEP1s)*, wild type L3-5 mosquitoes expressing the *TEP1r* allele (*TEP1r*), mosquitoes transgenically expressing *TEP1r* in a wild type *TEP1s* background (*TEP1s;Vg-TEP1r*), mutant *TEP1* mosquitoes (*TEP1^ΔT^*), and mutant mosquitoes transgenically expressing *TEP1r* (*TEP1^ΔT^;Vg-TEP1r*). Ookinetes express GFP (green) and nuclei are stained with Dapi (blue). Images are stacks of confocal z-sections, scale bar = 20 μm

*P*. *berghei* infection of *TEP1Δct2;Vg-TEP1r* was strikingly reduced compared to mutant controls, with some live oocysts and mostly melanized parasites (Fig 4a), indicating that the introduced TEP1r is fully functional and sufficient to kill parasites. Infection prevalence (S2 Table) in the transgenic is also significantly lower than in control *Vg-GFP* mosquitoes that express TEP1s only. *P*. *berghei* infection of *TEP1ΔT;Vg-TEP1r*, in which only the full form of TEP1 is detected, also resulted in a significant reduction in live oocyst numbers and in the concomitant appearance of high numbers of melanized parasites in some midguts compared to the non-rescued mutant (Fig 4b). High levels of melanization are reminiscent of the L3-5 infection phenotype, where complete refractoriness is achieved by TEP1r-mediated parasite killing followed by parasite melanization [19,33]. However, contrary to L3-5 that are completely refractory to the parasite, the rescued mutant lines which derive from the susceptible G3 background are not fully refractory to *P*. *berghei* infection, with about 70% of infected midguts containing live oocysts 7 days after infection (S2 Table) and infection intensities comparable to wild-type control G3 mosquitoes. In fact, expression of *TEP1r* in mutant backgrounds rescued the mutant at least to the level of the wild-type TEP1s-carrying G3 line in terms of prevalence and intensity, and further conferred a gain-of-function phenotype (melanization) (Fig 4a). In the *TEP1ΔT;Vg-TEP1r* line, it is striking that transgenic TEP1r appears to be functional in spite of its impaired maturation into the TEP1-cut form. These results imply either that TEP1 cleavage may be dispensable for some of its functions, or that low levels of TEP1-cut that we could not detect in the hemolymph (Fig 3b and 3e) or on parasites (Fig 5) may be sufficient for function. The formation of TEP1-cut at foreign surfaces is dependent on a convertase complex controlled by SPCLIP1 [39]. To test if maturation of TEP1-full into TEP1-cut is required for the observed phenotype, we silenced *SPCLIP1* by dsRNA injection in *TEP1ΔT;Vg-TEP1r* mosquitoes (S1 File). This fully abolished melanization and tended to increase parasite numbers, suggesting that some TEP1r cleavage by the convertase complex, though undetectable, must be required for activity of the *TEP1r* transgene.

Importantly, transgenic expression of *TEP1r* exclusively in the fat body using the *Vg* promoter rescued the *TEP1* loss-of-function mutations (Fig 4a and 4b), showing that TEP1 produced in the fat body and secreted into the hemolymph is sufficient for parasite killing.

We tested whether expressing *TEP1r* in a wild type background where *TEP1s* is present could also reduce parasite loads after *P*. *berghei* infection. Oocyst counts revealed no difference in live oocyst numbers between the parental *TEP1s* line and the *TEP1s;Vg-TEP1r* transgenic line (Fig 4c, left panel). However, the number of melanized parasites was significantly elevated in these mosquitoes (Fig 4c, right panel). We verified that the increase in melanized parasites was attributable to the *TEP1r* transgene using allele specific RNAi. When transgenic *TEP1r*, but not endogenous *TEP1s*, was knocked down in the *TEP1s;Vg-TEP1r* transgenic line, the number of melanized parasites reverted to a level similar to control *dsLacZ*-injected mosquitoes (Fig 4d).

In the *TEP1s*;*Vg-TEP1r* line, transgenic *TEP1r* is induced to a peak level about 24 hours after a blood meal, possibly too late to efficiently fight parasite invasion. To induce *TEP1* expression prior to the infectious blood meal and attempt to increase TEP1 concentration in the hemolymph, we offered a non-infectious blood meal and infected mosquitoes three days later, before the *Vg* promoter had returned to its off state. The results of such an experiment (S4 Fig) show no difference in infection levels between mosquitoes that were pre-induced by blood meal and naïve mosquitoes.

### Binding of TEP1 to parasites

We verified that transgenic TEP1r binds to parasites in the *TEP1Δct2;Vg-TEP1r* line (S5 Fig) and asked whether this was also the case in *TEP1ΔT;Vg-TEP1r* mosquitoes in which formation of the mature TEP1-cut form is inhibited. To this end, we performed immuno-histochemical staining of *TEP1ΔT;Vg-TEP1r* midguts 24h after infection using TEP1 specific antibodies (Fig 5). Results show that in the presence of TEP1ΔT, transgenic TEP1r protein is not detectable on ookinete surfaces, despite its observed, SPCLIP1 dependent, anti-parasitic and melanization activity.

**Fig 6:**
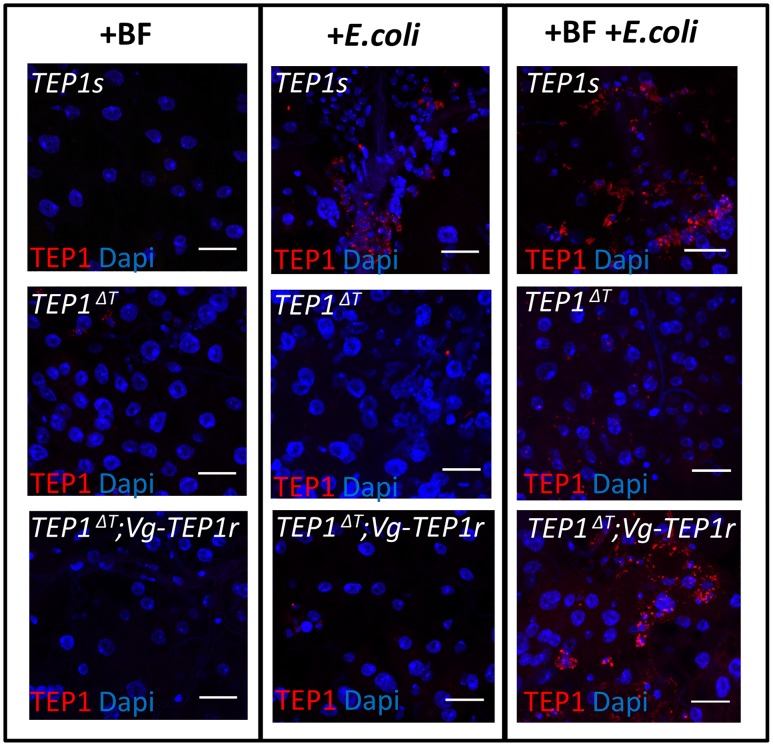
Fat body expressed transgenic TEP1 enters hemocytes. TEP1 antibody staining (red) of dissected mosquito carcasses after injection of heat killed *E*.*coli*. Nuclei are stained with Dapi (blue). Shown are wild type (*TEP1s*), *TEP1s* mutant (*TEP1*^*ΔT*^) and mutants complemented with transgenic *TEP1r* under control of the *Vg* promoter (*TEP1*^*ΔT*^*;Vg-TEP1r*). Compared are blood fed non injected (+BF), non blood fed injected (+*E*. *coli*) and blood fed, *E*. *coli* injected mosquitoes (+BF+ *E*. *coli*). scale bar = 20 μm.

We tested whether the fat body-expressed, full length TEP1r in the *TEP1ΔT;Vg-TEP1r* line can enter hemocytes after bacterial injection. To this end we examined TEP1 staining in carcasses of blood fed mosquitoes, injected with heat killed *E*. *coli*, 24h after blood meal (Fig 6). Results show that in blood-fed *TEP1ΔT;Vg-TEP1r* mosquitoes, TEP1r artificially induced in the fat body by the blood meal accumulated in the hemocytes only when mosquitoes were injected with *E*. *coli*. Control wild type mosquitoes showed that TEP1 accumulation in hemocytes depended on bacterial injection, regardless of blood feeding. The *TEP1ΔT* mutant line, used here to control for the specificity of antibodies for TEP1, showed no TEP1 staining in hemocytes under any condition. These results show that TEP1 detection in hemocytes can result from uptake of what is most likely TEP1-labeled bacteria.

### Optimization of synthetic transcription factors to over-express endogenous *TEP1*

Since transgenic expression of *TEP1r* in a wild-type *TEP1s* background failed to produce refractory mosquitoes, we asked whether artificially elevating the levels of the endogenous *TEP1s* allele may lead to increased immunity against *Plasmodium*. To this aim, we stimulated expression of the endogenous *TEP1* gene using synthetic transcription factors designed to bind specific promoter sequences.

Transcription Activator Like Effectors (TALEs) are transcription-activating proteins derived from *Xanthomonas* bacteria, containing a DNA-binding domain composed of nucleotide-binding repeats that can be arranged in the desired order to bind chosen DNA sequences [41,42,43]. In order to trigger over-expression of the endogenous *TEP1* gene, we designed TALE proteins that bind specific DNA sequences in the promoter region of *TEP1*. To find the best potential binding position for a TALE, we first analyzed the 3 kb promoter region of *TEP1* (S6 Fig). Using a luciferase reporter assay in *Drosophila* S2 cells and nested promoter deletions, we found that a 250 bp fragment of the promoter was sufficient to induce reporter activity. The minimal promoter sequence, harboring a potential NF-κB binding site, responded to the addition of bacteria to the culture medium by increased luciferase activity (S7 Fig). Mutating this NF-κB binding site abolished the reporter response to bacterial challenge (S8 Fig). This suggests that the NF-κB pathway of S2 cells can activate the mosquito *TEP1* promoter, in agreement with the known activation of native *TEP1* transcription by the mosquito NF-κB pathway [35]. We used this minimal promoter to test several transcriptional activation domains for reporter activation by TALEs, namely those of yeast Gal4, *Drosophila* Heat Shock Factor 1 and Herpes virus VP16, and found that the VP16 activation domain fused to a TALE DNA-binding domain leads to highest reporter induction (S9 Fig). The VP16 domain was thus chosen as the activation domain for all further designed TALEs. TALEs without an activation domain did not increase *TEP1* expression and served as negative controls.

**Fig 7:**
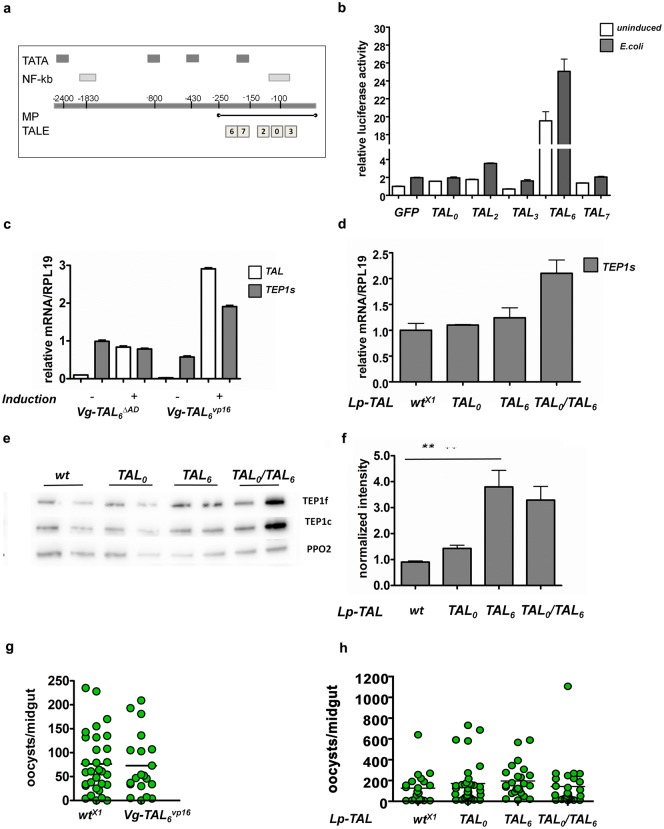
Synthetic TALE transcription factors targeting the *TEP1* promoter. **a**. Schematic representation of the ~3Kb *TEP1* promoter (not to scale). Shown are potential NF-κB sites (GGGRNNYYCC) (light grey boxes) and potential TATA boxes (dark grey boxes). The minimal promoter (MP) shown to be active in cells is indicated by a black line. Numbers on the promoter indicate the distance from the ATG. White boxes represent the binding position of each designed TALE. **b**. Luciferase assay in S2 cells showing the effect of TALEs on the minimal *TEP1* promoter. Different TALE constructs all carrying a VP16 activation domain and placed under the *Drosophila actin5c* promoter were co-transfected into S2 cells together with a firefly luciferase reporter gene under control of the minimal *TEP1* promoter. A plasmid carrying *GFP* placed under the same promoter as TALEs was used as reference for basal promoter activity. The *TEP1* promoter was further induced by addition of heat killed *E*. *coli* to the cells. Luciferase activity was normalized to the basal promoter activity without TALEs. **c**. qRT-PCR to assess the level of *TALE* and *TEP1* mRNA in transgenic mosquitoes expressing *Vg-TAL*_*6*_ comparing functional TALE with VP16 activation domain (*Vg-TAL*_*6*_^*vp16*^) and control line expressing the same TALE without activation domain (*Vg-TAL*_*6*_^*ΔAD*^), with or without induction of the *Vg* promoter by a blood meal 24h prior to mRNA extraction. **d.** qRT-PCR of transgenic mosquitoes constitutively expressing *Lp-TAL*_*6*_ or *Lp-TAL*_*0*_ or both, showing the level of *TEP1* mRNA. Levels of mRNA in each sample are normalized to *wt* and to the housekeeping reference gene *RPL19*. **e** Western blot analysis of transgenic mosquitoes constitutively expressing *Lp-TAL*_*6*_ or *Lp-TAL*_*0*_ or both. Wild type mosquitoes are used as controls. Shown are the bands for the full length and the cleaved forms of TEP1 as well as PPO2 as loading control. 2 samples for each mosquito line are presented to show sample variation. **f**. Quantification by densitometry of the TEP1 bands in (**e**). **g**. *P*. *berghei* infection of *Vg-TAL*^*vp16*^ transgenic mosquitoes. **h.** *P*. *berghei* Infection of transgenic mosquitoes constitutively expressing *TAL*_*0*_, *TAL*_*6*_ or both under the *Lp* promoter. In all infections, the empty docking line (wt^X1^) serves as control. Live oocysts (green circles) in each midgut are counted 7 days after infection. Number of oocysts and infection data are in S3 Table.

We constructed five TALEs that bind at different positions along the *TEP1* minimal promoter sequence (Fig 7a and S6 Fig) and tested their effect on reporter transcription in S2 cells (Fig 7b). Results show that TAL_3_ binding the closest to the transcription start site had no effect on reporter activity, while the other TALEs had a modest effect, increasing activity by about 1.5–2 fold. However, TAL_6_, binding the furthest from the transcriptional start site and just upstream of a sequence resembling a TATA box, triggered a strong, 20 fold increase in reporter activity. TAL_6_ was thus chosen to create transgenic mosquitoes with the aim of over-expressing endogenous *TEP1*.

In plants, natural and artificial TALEs induce target gene expression from alternative, TALE-specific transcription start sites [44,45]. We investigated whether TALEs triggered *TEP1* expression through an alternative transcription site when transfected with the luciferase reporter in S2 cells. PCR products from the 5' RACE assay were sequenced and revealed that unlike in plants, both TAL_0_ and TAL_6_ preserved the normal transcriptional start site of the mRNA which remained located 43 bp upstream of the ATG (S6 Fig).

### Transgenic mosquitoes expressing TALEs to drive endogenous *TEP1* expression

We generated several transgenic lines that express TALEs designed to bind and activate the *TEP1* promoter. Our first choice of promoter for the expression of the synthetic transcription factor itself was the inducible fat body specific *Vg* promoter, in order to increase endogenous *TEP1* levels after a blood meal, concomitant with *Plasmodium* parasite invasion. TAL_6_ fused to a VP16 (TAL_6_^vp16^) activation domain was chosen due to its high activity in S2 cells. A control transgenic line was created with the same TAL_6_ lacking an activation domain (TAL_6_^ΔAD^). Analysis of mRNA after blood feeding revealed that in both transgenic lines TALEs are induced by a blood meal, more efficiently in the case of Vg-TAL_6_^vp16^ (Fig 7c). Furthermore, TAL_6_^vp16,^ but not TAL_6_^ΔAD,^ induced a two-fold elevation in *TEP1* mRNA levels. This elevation in the endogenous levels of *TEP1* mRNA is modest compared to the 20-fold average elevation of the reporter in the cell line.

Since the *Vg* promoter triggers a pulse of transcription after a blood meal, TALEs may activate TEP1 too transiently to achieve a significant effect. Therefore, we also tested if a constitutive fat-body promoter would durably induce high levels of endogenous *TEP1*. For this, TAL_6_^vp16^ and TAL_0_^vp16^ were placed under the control of the *Lp* promoter, constitutively expressed in the fat body in larvae, pupae and adults. We included TAL_0_^vp16^ in addition to TAL_6_^vp16^ despite its modest effect in S2 cells (~1.5 fold increase, Fig 7b) because of its unique binding position on the NF-κB binding site. TAL_0_^vp16^ was designed to bind and activate not only the promoter of *TEP1*, but also similar sequences found in the promoters of *APL1C* and *LRIM1*, two proteins that are known to bind TEP1 and contribute to its activity (See Methods).

Analysis of *TEP1* mRNA in transgenic mosquitoes that constitutively express TAL_6_^vp16^ and/or TAL_0_^vp16^ (Fig 7d) revealed a maximum of 2-fold induction of endogenous *TEP1* mRNA in mosquitoes that expressed both TALES simultaneously (progeny of a cross between the two lines). Western blot analysis of TEP1 in these mosquitoes (Fig 7e) confirmed a 3–4 fold increase in TEP1 protein levels circulating in the hemolymph (Fig 7f). We thus proceeded to test whether this elevation in TEP1 levels in the hemolymph had any effect on parasite loads after *Plasmodium* infection.

Infection of the blood meal inducible *Vg*-*TAL*_*6*_^*vp16*^ transgenic line did not result in any significant change in parasite loads (Fig 7g). Likewise, analysis of parasite loads after infection of the constitutively expressing *Lp-TAL*_*6*_^*vp16*^ and *Lp-TAL*_*0*_^*vp16*^ lines did not show any significant difference in oocyst numbers between transgenic and control lines (Fig 7h).

## Discussion

### *TEP1* pathway genes are expressed mainly in the fat body

TEP1 is a secreted protein that circulates in the hemolymph of mosquitoes at all developmental stages starting from larval stages [20]. Previous observations suggested that TEP1 is produced by hemocytes and secreted from these cells as a full-length protein which is then cleaved in the hemolymph [20]. Evidence to support this model included immuno-histochemical staining of mosquito tissues that showed TEP1 accumulation inside hemocytes, particularly after bacterial challenge and 24-48h after infection with *P*. *berghei* [22,35,46]. However, these results may also be explained by uptake of TEP1 protein, or TEP1-labeled particles, by hemocytes. In addition, analysis of RNA expressed by hemocytes using microarrays has shown that *TEP1* transcript is present in these cells, but not enriched compared to whole female tissues, and is not further induced by infection [47]. *TEP1* did not emerge as a hemocyte transcript in the hemocyte transcriptomics study of Pinto *et al*. [48]. Of note, it is technically challenging to acquire a pure population of hemocytes without contamination by fat body cells that detach into the hemolymph during sample collection [49]. Here we obtain evidence that *TEP1*, *LRIM1* and *APL1C* are expressed primarily in the fat body. This is similar to the expression pattern of many insect anti-microbial peptides [50,51] and to that of some *Drosophila TEP* family member genes as determined by *in situ* hybridization [52]. In mammals, complement proteins phylogenetically and functionally homologous to *TEP1* are expressed and secreted from the liver ([53],reviewed in [54]). Interestingly, some of the functions of the liver are performed by the fat body in insects.

We crossed the *TEP1*-GFP reporter mosquitoes to our hemocyte-specific *PPO6*-RFP line, and observed that the DsRed-positive hemocytes were GFP negative. We note that this result does not rule out that sub-populations of hemocytes not expressing the *PPO6* promoter may also express *TEP1*; fine transcriptomic analyses will be required to quantitatively determine the contribution of each tissue to the global pool of circulating TEP1.

Our transgenic reporter lines also reveal that *TEP1*, but not *APL1* and *LRIM1*, is also expressed in the proventriculus of adult mosquitoes. Interestingly, the proventriculus was found to be a site of immune gene expression in *A*. *gambiae* [55] and in other insects such as tsetse flies [56]. In *A*. *gambiae*, mRNA transcripts of antimicrobial peptides *Defensin 1*, *Cecropins*, *Gambicin*, as well as *TEP1*, are enriched in the proventriculus [55]. *TEP1* is also expressed in the neighboring gastric caeca during larval stages [57]. Future studies may clarify whether proventriculus-expressed *TEP1* has a function that bypasses the requirement for LRM1 and APL1-C, perhaps in the lumen of the digestive tract where TEP1 could interact with the gut microbiota.

### *TEP1-GFP* reporter expression does not reflect immune status

Variation in GFP levels among individual mosquitoes led us to test whether GFP fluorescence intensity correlates with infection outcome. We found no such correlation and concluded that *GFP* expression either did not reflect TEP1 levels in the mosquitoes at the time of *P*. *berghei* infection, or that TEP1 level variations do not correlate with resistance to the parasite. The lack of correlation between GFP levels and immune status may be due to the remarkable stability of GFP protein, which persists throughout mosquito life in the fat body after a blood feeding-induced pulse of expression in the *Vg-GFP* line [34] compared to the highly dynamic turnover of TEP1 protein, which disappears by 24h after RNAi silencing [37].

### Transgenic expression of *TEP1* in the fat body

Lysis of ookinetes at the basal lamina is associated with binding of TEP1 directly to the ookinete surface [19]. However, TEP1 does not induce direct lysis of bound pathogens, and the molecular mechanism of killing remains unknown. It has been previously shown that TEP1-cut is the major form that binds ookinetes [21,22]. Binding of circulating TEP1-cut to pathogen surface triggers the recruitment of a convertase complex that requires the serine protease homologue SPCLIP1 and that cleaves circulating TEP1-full leading to further accumulation of TEP1-cut at the pathogen surface [38,39]. We expressed the *TEP1r* allele in two different *TEP1* mutant backgrounds: *TEP1ΔT* carries a single threonine deletion that appears to destabilize the protein, as mutant TEP1ΔT protein becomes faintly detectable only in aged mosquitoes [27]. TEP1Δct2 lacks the C-terminal third of the protein and is likely to be a complete null. In these two mutant backgrounds, transgenic *TEP1r* produced in the fat body and secreted into the hemolymph was sufficient to rescue parasite killing, and to trigger parasite melanization. Interestingly, in the *TEP1ΔT* background, but not in the *TEP1Δct2* background, maturation of transgenic TEP1r-full to TEP1-cut appeared to be inhibited as we never detected circulating TEP1-cut in the hemolymph, even after bacterial injection. This suggests that very low amounts of the non-functional TEP1ΔT protein inhibit maturation of TEP1-full, perhaps by blocking the enzyme responsible for TEP1 cleavage in the hemolymph. Possibly related to this, LRIM1/APL1C levels are reduced in the *TEP1ΔT*, but not in the *TEP1Δct2* mutant. In the *TEP1ΔT*;*VgTEP1r* mosquitoes, TEP1 binding is not detectable by antibody staining on ookinete surfaces, consistent with the lack of detectable circulating TEP1-cut form. Yet, both mutants rescued with *TEP1r* showed an increase in parasite melanization. We initially hypothesized that contrary to current views, processing of TEP1-full to TEP1-cut by the SPCLIP1-dependent convertase and accumulation of TEP1-cut at the parasite surface may be dispensable for parasite melanization in some contexts. However, disabling the TEP1 convertase complex through silencing SPCLIP1 in *TEP1ΔT*;*VgTEP1r* mosquitoes abolishes melanization and, to some extent, increases parasite numbers. Therefore, conversion of TEP1-full to TEP1-cut seems to be a prerequisite for parasite killing and melanization even in *TEP1ΔT;VgTEP1r* mosquitoes, but a large accumulation of TEP1-cut at the surface of parasites may not be necessary to trigger killing and melanization.

Another function of TEP1 in the hemolymph is the opsonization of bacteria [20,39,58,59], marking them for phagocytosis by hemocytes. Shortly after the injection of bacteria, TEP1 protein can be detected inside the hemocytes that are attached to dissected mosquito carcasses. The appearance of TEP1 inside these cells may be due to induced expression of *TEP1* within hemocytes, presumably aimed at replenishing TEP1 levels in the hemolymph, or due to phagocytosis of TEP1-covered bacteria by hemocytes. We observed that in a *TEP1* null mutant background, *TEP1r* expressed from a transgene in the fat-body could enter hemocytes. Our results suggest that TEP1 detected in hemocytes after bacterial challenge comes at least in part from uptake of circulating TEP1 protein.

*TEP1r* could be a candidate gene for creating malaria resistant mosquitoes by driving the gene into the wild mosquito population, for example using CRISPR-Cas9 gene drive [14,15]. This should not be achieved by replacement of endogenous *TEP1s* alleles with the more active *TEP1r*, as a few failed gene conversion events could result in Cas9-induced *TEP1s* loss-of-function mutations that would generate hyper-susceptible mosquitoes. Instead, the anti-parasitic factor should be driven into an independent neutral genomic locus. We thus tested whether over-expression of *TEP1r* in wild type background affects infection outcome. We found that transgenic *TEP1r* is indeed expressed in the presence of endogenous *TEP1s*, but fails to enhance parasite killing, while still able to stimulate melanization. Using allele specific knock-down we showed that the allelic difference between *TEP1r* and *TEP1s* is sufficient to explain the different intensities of the melanization reaction. In addition, silencing endogenous *TEP1s* in the same mosquitoes restored the ability of transgenic *TEP1r* to kill parasites and further elevated melanization (Fig 4d), as observed when transgenic *TEP1r* was expressed in a mutant background. This indicates that although less efficient than TEP1r for parasite killing, endogenous TEP1s protein is able to outcompete transgenic TEP1r for the parasite killing function. This is reminiscent of the apparent recessivity of *TEP1r* in *TEP1s* x *TEP1r* genetic crosses [25]. Therefore, simple transgenic expression of *TEP1r* in a wild type background where *TEP1s* is the most frequent allele would not be sufficient to render a mosquito population refractory to *Plasmodium* infection.

### Over-expressing endogenous *TEP1*

Since transgenic expression of *TEP1r* in a wild-type *TEP1s* background failed to produce refractory mosquitoes, we asked whether artificially elevating the levels of the endogenous *TEP1s* allele may lead to increased immunity against *Plasmodium*. To this aim, we stimulated expression of the endogenous *TEP1* gene using synthetic transcription factors designed to bind specific sites in the *TEP1* promoter.

We designed a luciferase reporter for *TEP1* promoter activity and tested it in *Drosophila* S2 cells. The promoter contained an NF-κB binding site, which mediated elevation of reporter activity when S2 cells were treated with heat-killed bacteria. Thus, the cloned *TEP1* promoter fragment is activated by the S2 cell NF-κB pathway, consistent with *TEP1* activation by the mosquito NF-κB pathway [35].

Once we determined the optimal TALE sequence for binding *TEP1* promoter, we created several transgenic mosquitoes lines that expressed TALEs either under an inducible promoter (*Vg*) or a constitutive fat body promoter (*Lp*). In all cases, the increase in endogenous *TEP1* expression was very modest both at mRNA and protein levels, indicating that cell culture assays are poor predictors of TALE activity *in vivo*. *TEP1* mRNA levels may be tightly regulated *in vivo*, or may depend on regulatory features that were absent or inactive in the S2 cells assays.

In wild type mosquitoes, some parasites are bound by TEP1 and killed, while others evade TEP1 binding. If the quantity of circulating TEP1 were the rate-limiting factor in parasite binding, we expected that even a modest 2-fold increase in *TEP1* expression would yield increased resistance to *Plasmodium*. However, infection assays with mosquitoes that expressed TALEs and showed a 2 to 3-fold elevation in TEP1 levels did not show any increase in resistance. This is similar to the lack of increased parasite killing in mosquitoes expressing transgenic *TEP1r* in addition to endogenous *TEP1s*, and suggests that the survival of some parasites is not due to a lack of TEP1. The absence of effect of the TALE-mediated over-expression of *TEP1s* contrasts with the elevated melanization obtained by transgenic expression of *TEP1r* in mosquitoes carrying endogenous *TEP1s*, again confirming that enhancing melanization is an allelic property of *TEP1r*.

To conclude, our results indicate that simply augmenting the level of a given anti-parasitic factor may not be sufficient to achieve greater resistance levels, and biotechnological approaches aiming at rendering the mosquitoes resistant to malaria will need to integrate the full complexity of molecular interactions between immune factors.

## Methods

### Mosquito rearing and *P*. *berghei* infection

*A*. *gambiae* mosquitoes were maintained in standard conditions (28°C, 75–80% humidity, 12-hr/12-hr light/dark cycle). Larvae were raised in deionized water and fed finely ground TetraMin fish food. Adults were fed on 10% sucrose *ad libitum* and females were blood-fed on anaesthetized mice. For infection, mosquitoes were fed on CD1 mice infected with the *P*. *berghei* GFP-con 259cl2 clone (ANKA strain) that constitutively expresses GFP [60]. Mice infected with *Plasmodium berghei* were sacrificed before they developed malaria symptoms by cervical dislocation. Mice used to blood feed and infect mosquitoes were anesthetized with a mix of xylazine and ketamine. Infected mosquitoes were maintained at 21°C, unfed mosquitoes were removed 24h post feeding. Seven days after infection, live midguts were mounted on microscope slides in PBS, photographed under fluorescent light, and parasites were counted on the pictures. Statistical significance of differences in parasite counts was evaluated with a Mann-Whitney non-parametric test.

### Mosquito transgenesis

Transgenic mosquitoes were created in the *A*. *gambiae* X1 *attP* docking line using the pDSA R/T/G/Y/P and pattB-RfB2 transgenesis vectors as described [34]. The following plasmids were constructed by Goldengate cloning to generate transgenic mosquitoes: pDSAP*-LRIM1-GFP*, pDSAT*-APL1-GFP*. In the case of the *TEP1-GFP*,*3xP3-RFP* reporter and of the *Vg-TEP1r*,*3xP3-YFP* constructs, plasmids were assembled using Gateway cloning into pattB-RfB2 [34]. *Vg-TALE and Lp-TALE* constructs were assembled by Goldengate cloning in pDSAR, pDSAT or pDSAG as described for TALEs [42]. Plasmid sequences, as well as promoter and TALE modules are provided in supplementary file S1 Text. In the case of TAL_0_, we exploited the TAL repeat “NS” di-residue binding ambiguous bases to allow simultaneous targeting of the *TEP1*, *LRIM1* and *APL1C* promoters by TAL_0_ (target sites indicated in S4 Table). Transgenic lines *PPO6-RFP*,*3xP3-YFP* and *Lp-RFP*,*3xP3-GFP* and *Vg-GFP*,*3xP3-RFP* were previously described [34]. Promoters and genes were amplified from genomic DNA or cDNA by PCR with primers containing appropriate sites for Goldengate or Gateway cloning. All constructs were verified by sequencing.

To introduce the *TEP1r*,*3xP3-YFP* transgene in the background of the *TEP1*^*ΔT*^ and *TEP1*^*Δct2*^ mutations [27], the transgenic line was backcrossed three times to the mutant line. After each backcross, YFP-positive progeny larvae were purified with the COPAS flow cytometer [61] to retain the transgene. After the last backcross, one leg from 48 male and 48 female mosquitoes kept alive in individual tubes was analyzed by PCR as described [27] using the Phire Direct Tissue PCR kit (Thermofisher). Mosquitoes showing homozygosity for the *TEP1* mutation were pooled to establish a homozygous mutant and heterozygous transgenic line. For experiments, COPAS sorting of this line was used to generate cultures containing 50% non-transgenic mutant control and 50% transgenic mutant mosquitoes. In some cases, non-mutant control mosquitoes with RFP eye fluorescence (such as *Vg-GFP*,*3xP3-RFP*) were added to the same culture. On the day of dissection, transgenic and non-transgenic mosquitoes were separated under a fluorescence microscope based on the eye fluorescence of the transgenesis markers. This process ensured identical exposures of all mosquito genotypes to the same growth and environmental conditions.

### Knockdown of *TEP1* by dsRNA

Allele specific knockdown of *TEP1* was performed by injection of dsRNA targeted specifically against *TEP1r* or *TEP1s* as previously described [25]. dsRNA was injected into the thorax of one day old female mosquitoes, three days prior to infection. Allele specific knockdown was verified by qRT-PCR. *SPCLIP1* RNAi knockdown was performed as described [39][62].

### Immuno-histochemistry

Mosquitoes were dissected in PBS, fixed in paraformaldehyde for 20 min, permeabilized in PBS containing 0.1% triton (PBST) for 10 min, blocked in PBST containing 1%BSA (PBSTB) for one hour. Mosquito tissues were incubated with primary antibody (rabbit anti TEP, mouse anti Lipophorin) for at least two hours, washed 3 times and incubated with fluorescent secondary antibody (Alexa488, or Cy3) in PBSTB containing 1μg/μl DAPI. Tissues were washed three times in PBST and mounted on a slide with a drop of Aqua/poly mount (Polysciences). For fat body analysis, mosquitoes were dissected and treated as previously described [63]. Images were collected under a Zeiss LSM 710 confocal microscope.

### RT-PCR

RNA for RT PCR was extracted from groups of 5–10 mosquitoes. Mosquitoes were collected on ice into 300 μl RNAzol RT (MRC), homogenized with a Precellys homogenizer, with 2 pulses of 20 seconds. RNA was purified on DirectZol RNA columns (Zymo Research) following manufacturer's instructions. RNA was treated with RNAse-free DNAse I (ThermoFisher). Reverse transcription was performed using iScript cDNA Synthesis Kit (Bio-Rad) using 500 μg of RNA. SYBR Green PCR Master Mix, (Applied Biosystems) qPCR reactions were set up on 96 well plates with three technical and three biological repetitions and were run on 7500 Real-time PCR machine (Applied Biosystems).

### Western blot analysis

Mosquito hemolymph from 10 mosquitoes was collected by proboscis clipping directly into 20 μl of Laemmli buffer. Samples were heated at 90°C for 2–5 minutes and 2 to 8 μl (depending on the experiment) were loaded on polyacrylamide gels. Rabbit polyclonal antibodies against TEP1, APL1C, LRIM1, PPO2 and Vg were used. Secondary HRP conjugated antibodies were used at 1:15000 dilution. Images were collected on Fusion FX7 scanner (Vilber Lourmat).

### Luciferase assay in S2 cells

S2 cells were grown at 23°C in Schneider's medium (Biowest) supplemented with 10% FCS (Invitrogen) and 1% Penicillin/Streptomycin solution (Sigma). Cells were seeded in 24 well plates (Starsted) at 50,000 cells/well one day prior to transfection. Constructs expressing both firefly luciferase and Renilla luciferase were used as reporters. *TEP1* promoter was cloned upstream of firefly luciferase. Renilla luciferase under the constitutive actin5c promoter was used as transfection control. Cells were transfected using Effectene (Qiagen) according to manufacturer's instructions. Cells were collected three days after transfection for luciferase activity analysis. Luciferase assay was performed using the Dual-Luciferase Reporter Assay System (Promega) according to manufacturer's instructions. Luciferase activity is depicted as Firefly activity divided by Renilla activity, averaged on three wells transfected with a set of given plasmids.

### 5'RACE in S2 cells

RNA from S2 cells was extracted using RNAzol RT (MRC) and purified on DirectZol RNA columns (Zymo Research). 5' RACE was performed using First Choice RLM-Race kit (Ambion) according to manufacturer's instructions. Briefly, ligation of specific primers to 5' and 3' ends of the RNA allows the amplification of a PCR product that corresponds to the transcription start site of gene of interest. The resulting PCR product was cloned into CloneJet PCR Cloning Kit (ThermoFisher Scientific) followed by sequencing of several clones.

## Ethics Statement

This project was approved by the French Ministry for Research (agreement #555.01) upon review by its Ethics Committee for Animal Research CREMEAS #35, which approved the research. Experiments were carried out in conformity with the 2010/63/UE European animal bioethics legislation. Our animal care facility received agreement #F67-482-2 from the veterinary services of the region Bas-Rhin (Direction Départementale de la Protection des Populations).

## Supporting Information

S1 Table Summary of infections of *TEP1-GFP* reporter with *P*. *berghei*. Control mosquitoes and *TEP1-GFP* transgenic mosquitoes were grown together as larvae. Immediately prior to infectious blood feeding mosquitoes were selected according to their GFP expression level separating into two groups with low or high expression. 7 days after infection, oocysts were counted and compared between the three groups. n is the number of analyzed midguts in each condition. *p* values were calculated for the difference in oocyst numbers between low and high GFP expressing groups, using the non parametric Mann-Whitney t test.
(XLSX)

S2 Table Infection data for Fig 4. *n* is the number of analyzed midguts, Prevalence of infection is the number of midguts with at least one parasite out of the total number of midguts. Oocysts and melanized parasites were counted 7 days after infection. *p* values are from the non parametric Mann-Whitney t-test. Compared groups are marked in bold and are indicated in Fig 4.
(XLSX)

S3 Table Infection data for Fig 7. *n* is the number of analyzed midguts, prevalence of infection is the number of midguts with at least one parasite out of the total number of midguts. Oocysts were counted 7 days after infection. *P values* were calculated using non parametric Mann-Whitney t-test analysis.
(XLSX)

S4 Table Designed TALES and their Repeat-variable di-residues (RVDs). Taking advantage of the ambiguous recognition of nucleotides by the RVD NS, TAL0 was designed to target TRWSRSBRGGRAWTCCCS, thus targeting the following promoter sites: TGAGGGCAGGGAATCCCC (*TEP1*), TATCAGGAGGAAATCCCC (*LRIM1*) and TATCACTGGGAATTCCCG (*APL1C*).
(XLSX)

S1 Fig Expression of GFP in *TEP1-GFP*,*3xP3-RFP* reporter mosquitoes. **a**. GFP expression in the proventriculus of a blood fed female. **b**. GFP expression in proventriculus of a male. **c**. GFP expression in a blood fed female showing expression in Malpighian tubules (mt) and ovarian duct (od) of a blood fed female 24h after blood feeding. Right panel shows GFP expression in boxed ovary. **d**. Two transgenic larvae expressing GFP under the *TEP1* promoter. Note the strikingly different levels of GFP expression. GFP expression is in green, transgenic marker in red. **e**. Two live transgenic *TEP1-GFP* mosquito abdomens showing variable GFP expression.
(TIF)

S2 Fig Variability in GFP levels in transgenic TEP1-GFP larvae. 100 larvae were analyzed on each indicated day and the level of GFP expression was noted, ranging from no GFP to +++ (highest GFP expression). Shown are 4 independent experiments analyzing four different generations.
(TIF)

S3 Fig TEP1 levels in transgenic *TEP1s;Vg-TEP1r*. **a**. qPCR analysis of *TEP1r* mRNA in *TEP1s;Vg-TEP1r* transgenic mosquitoes expressing *TEP1r* under control of the *Vg* promoter. Mosquito RNA was analyzed at different time points after a blood feeding. Expression is relative to *RPL19* mRNA in each sample and normalized to time point zero hours after blood feeding. **b**. Western blot analysis of mosquito whole body (carcasses) and hemolymph showing that there is no increase in TEP1 protein levels in *TEP1s;Vg-TEP1r* mosquitoes. Control mosquitoes expressed GFP under the *Vg* promoter (*TEP1s;Vg-GFP*). PPO2 and actin were used as loading control for hemolymph and carcasses.
(TIF)

S4 Fig Binding of transgenic TEP1r to ookinetes. Control mosquitoes expressing *TEP1r* (left panel), show TEP1 binding (red) to the majority of GFP expressing *P*. *berghei* parasites (green). In *TEP1* mutant mosquitoes (middle panel) TEP1 does not bind to ookinetes and in mutants over-expressing *TEP1r* (right panel), transgenic TEP1 is bound to some of the parasites.
(TIF)

S5 Fig *P*. *berghei* infection of mosquitoes expressing transgenic *TEP1r* in wt background following prior induction by blood feeding. Compared are transgenic mosquitoes expressing *TEP1r* or *GFP* under the *Vg* promoter. For each mosquito group the un-induced (no prior blood meal) and the induced (blood fed on a non infected mouse 3 days before infection) are compared. Shown are live parasites (green circles) and melanized parasites (black circles) in the midguts of blood fed mosquitoes 7 days after infection. Infection data is given in S2 Table.
(TIF)

S6 Fig *TEP1* promoter architecture. **a.** Schematic representation of the 3 kb promoter fragment, indicating the position of potential TATA boxes (yellow), potential NF-κB binding sites (blue) and TALE binding sites (red). Drawing not to scale. **b**. Four TEP1 promoter fragments fused to a *luciferase* reporter gene. The position of potential TATA boxes (yellow), potential NF-κB binding sites (blue), TALE binding sites (red), *Luciferase* gene (light blue) and SV40 term (green) is shown. **c**. Sequence of the 251bp minimal promoter with TALE binding sites (red) and transcription start site (determined by 5'RACE) indicated by an arrow and 5'UTR. The GGGAATCCC NF-κB binding site is in the TAL0 recognition site indicated by Italic.
(TIF)

S7 Fig Analysis of the activity of successive truncations of the *TEP1* promoter region in S2 cells. Four different luciferase constructs (described in S3b Fig) were transfected into S2 cells and analyzed for Luciferase activity. For each condition, induction of the NF-κB pathway was triggered by addition of heat killed bacteria to cell medium. Depicted is luciferase activity in non-induced (white) and *E*. *coli* challenged (grey) cells. Luciferase activity is normalized to the activity in cells transfected with the full length promoter and uninduced. Shown is the average of three independent experiments. Transfected construct carries firefly luciferase under the control of TEP1 as well as Renilla luciferase under a constitutive promoter which serves as control. Luciferase activity is expressed as firefly luciferase activity divided by Renilla luciferase activity.
(TIF)

S8 Fig Determining the best activation domain for TALES. TALEs with the same DNA binding domain (TAL0) but different activation domains were co-transfected into S2 cells together with the Luciferase reporter under the control of the minimal TEP1 promoter fragment. Control (no TAL) were transfected with GFP, TALΔAD has no activation domain and serves as control, yeast GAL4, heat shock factor 1 transcription (HSF) and Herpes virus VP-16 domains were tested. Depicted is luciferase activity in uninduced (white) and induced (grey) cells. Luciferase activity is normalized to the activity in cells transfected with GFP and uninduced. Shown is a representative of at least 3 independent experiments. Error bars depict standard deviation from the mean of three biological repetitions.
(TIF)

S9 Fig Mutating the NF-κB binding site in the *TEP1* promoter abolishes induction by *E*.*coli*. S2 cells were transfected with two different reporters, one with a 250bp fragment of TEP1 promoter carrying an NF-κB binding site, or with the same promoter fragment where the NF-κB binding site was mutated (GGGAATCCCC to CGGAATACCG). Together with the reporter, cells were transfected either with a plasmid encoding GFP (no TAL) or a TAL6-VP16 construct. Depicted is luciferase activity with (grey) or without (white) *E*. *coli* challenge. Luciferase activity is normalized to relative to the activity in cells transfected with GFP and without *E*. *coli*. Shown is a representative of at least 3 independent experiments. Error bars depict standard deviation from the mean of three biological repetitions.
(TIF)

S1 File *SPCLIP1* silencing abolishes *P*. *berghei* melanization in *TEP1ΔT; Vg-TEP1r* mosquitoes. (DOCX)

S1 Text Sequences. Complete sequences of plasmids, as well as promoters and TALE domains that were used in this study.
(PDF)
