# Supplementary figures and images for: Transgenic Expression of the Anti-parasitic Factor TEP1 in the Malaria Mosquito Anopheles gambiae
Source: PLoS Pathog. 2017 Jan 17;13(1):e1006113. doi: 10.1371/journal.ppat.1006113 (PMC5240933; doi:10.1371/journal.ppat.1006113)

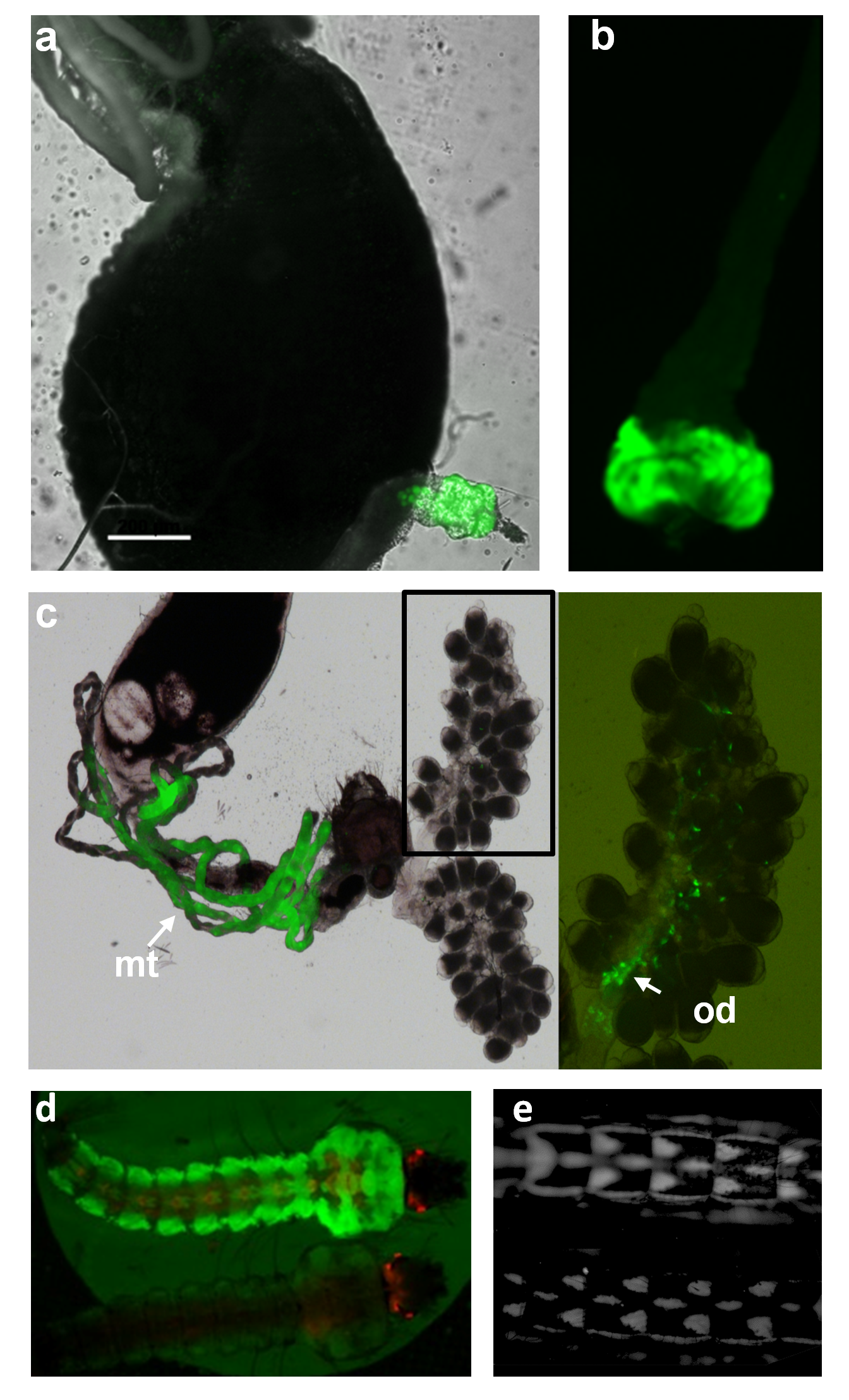

Supplement: S1 Fig — a. GFP expression in the proventriculus of a blood fed female. b. GFP expression in proventriculus of a male. c. GFP expression in a blood fed female showing expression in Malpighian tubules (mt) and ovarian duct (od) of a blood fed female 24h after blood feeding. Right panel shows GFP expression in boxed ovary. d. Two transgenic larvae expressing GFP under the TEP1 promoter. Note the strikingly different levels of GFP expression. GFP expression is in green, transgenic marker in red. e. Two live transgenic TEP1-GFP mosquito abdomens showing variable GFP expression. (TIF) [file ppat.1006113.s005.tif]

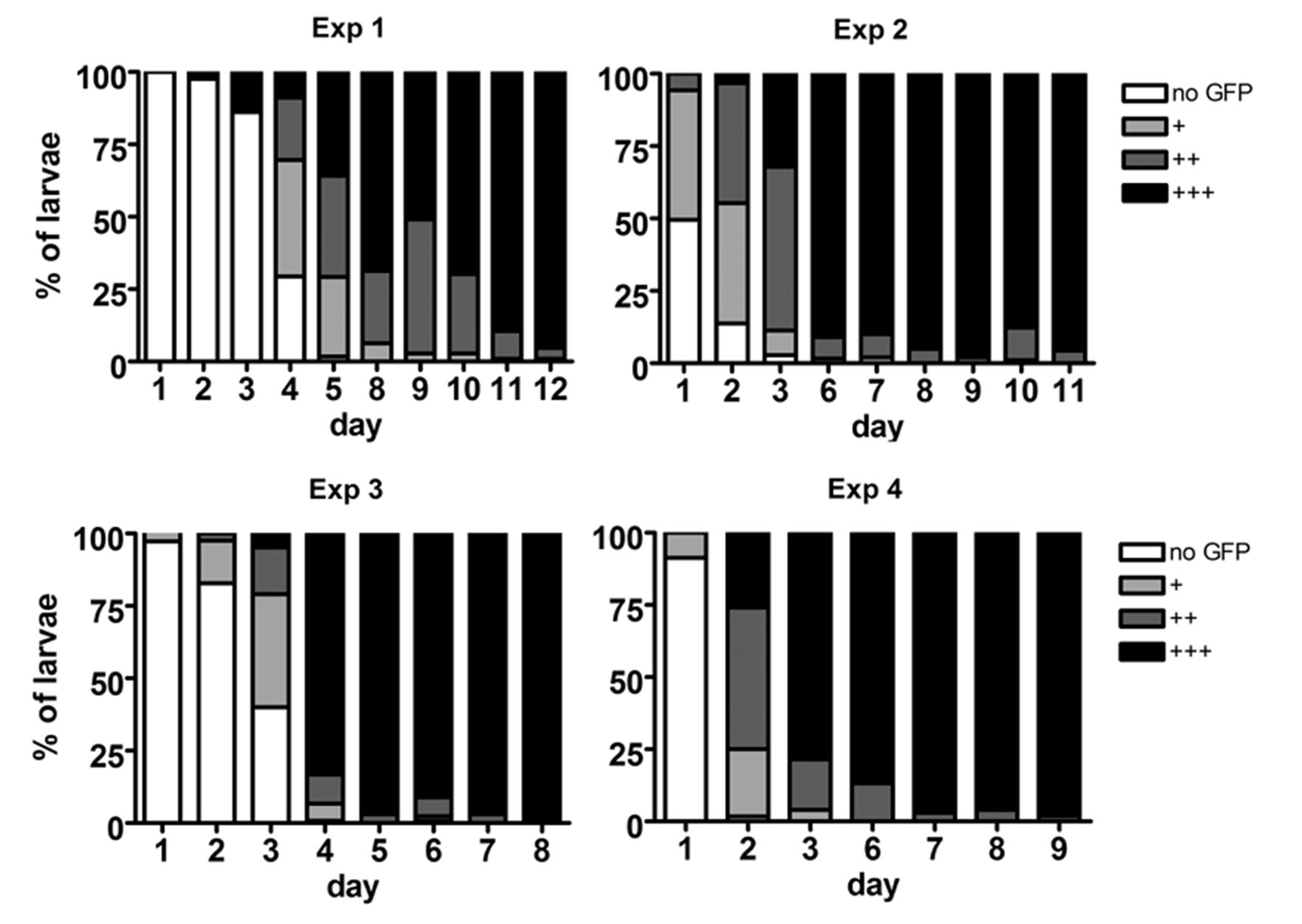

Supplement: S2 Fig — 100 larvae were analyzed on each indicated day and the level of GFP expression was noted, ranging from no GFP to +++ (highest GFP expression). Shown are 4 independent experiments analyzing four different generations. (TIF) [file ppat.1006113.s006.tif]

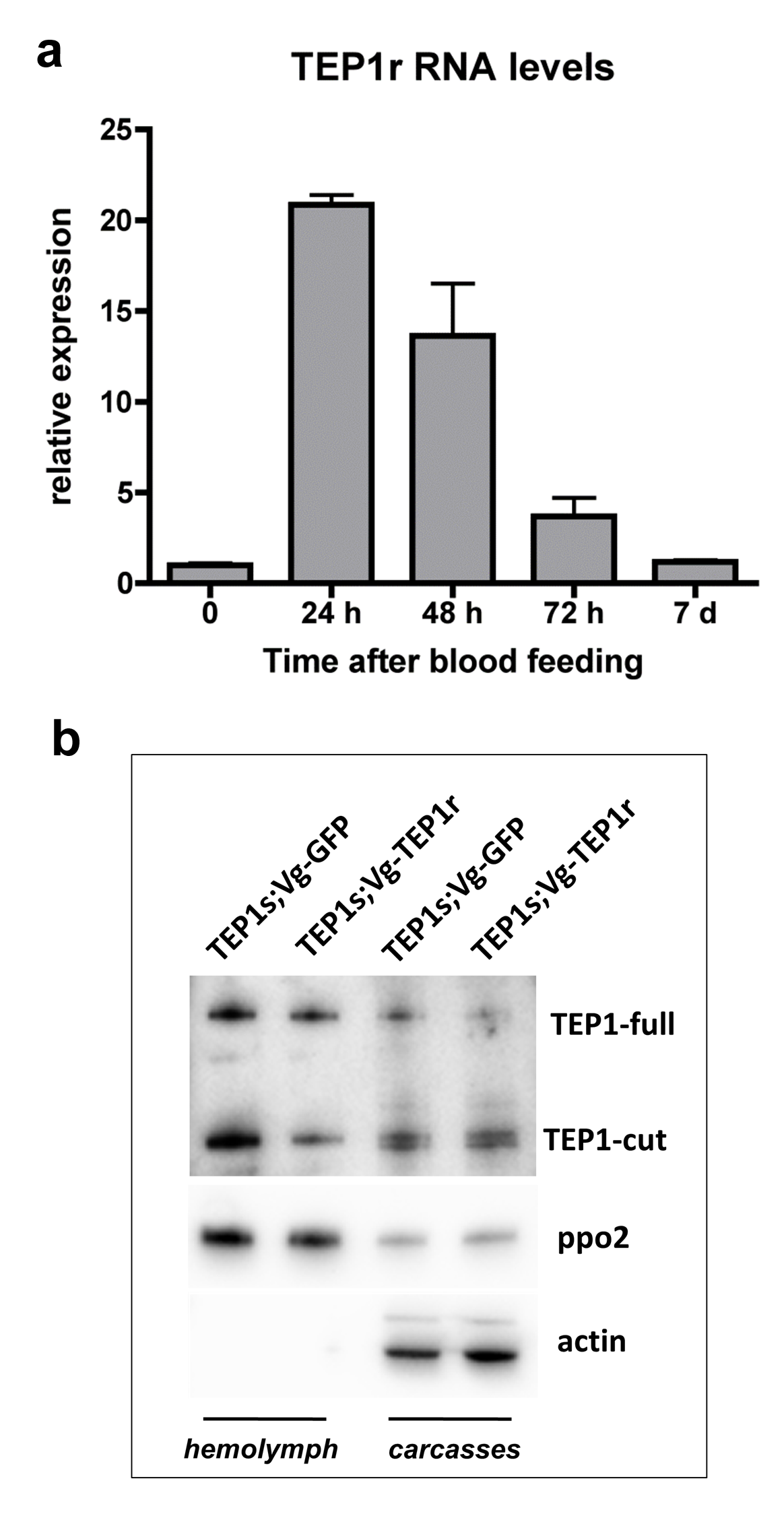

Supplement: S3 Fig — a. qPCR analysis of TEP1r mRNA in TEP1s;Vg-TEP1r transgenic mosquitoes expressing TEP1r under control of the Vg promoter. Mosquito RNA was analyzed at different time points after a blood feeding. Expression is relative to RPL19 mRNA in each sample and normalized to time point zero hours after blood feeding. b. Western blot analysis of mosquito whole body (carcasses) and hemolymph showing that there is no increase in TEP1 protein levels in TEP1s;Vg-TEP1r mosquitoes. Control mosquitoes expressed GFP under the Vg promoter (TEP1s;Vg-GFP). PPO2 and actin were used as loading control for hemolymph and carcasses. (TIF) [file ppat.1006113.s007.tif]

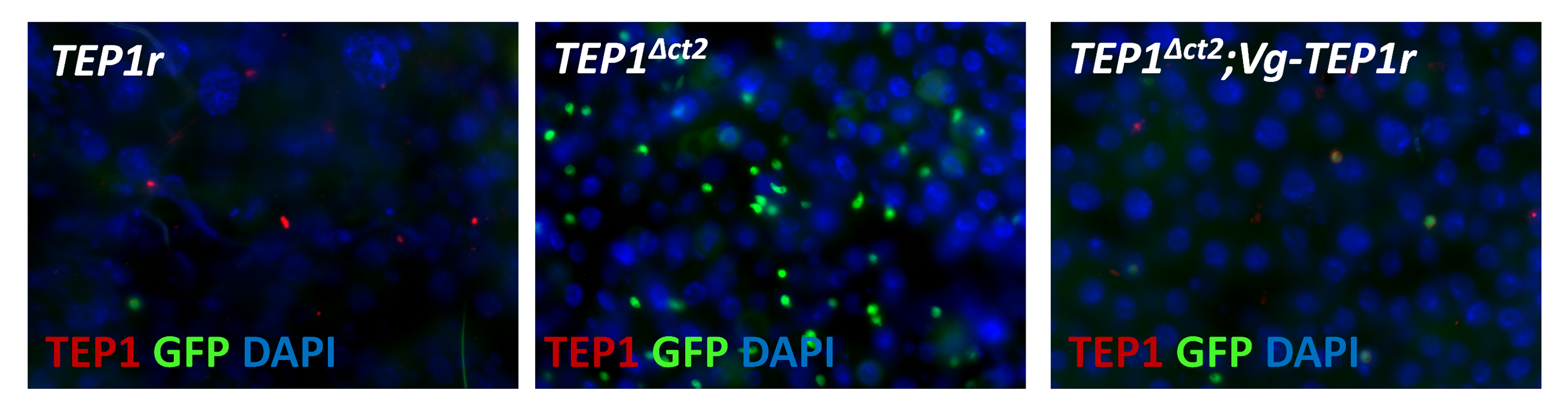

Supplement: S4 Fig — Control mosquitoes expressing TEP1r (left panel), show TEP1 binding (red) to the majority of GFP expressing P. berghei parasites (green). In TEP1 mutant mosquitoes (middle panel) TEP1 does not bind to ookinetes and in mutants over-expressing TEP1r (right panel), transgenic TEP1 is bound to some of the parasites. (TIF) [file ppat.1006113.s008.tif]

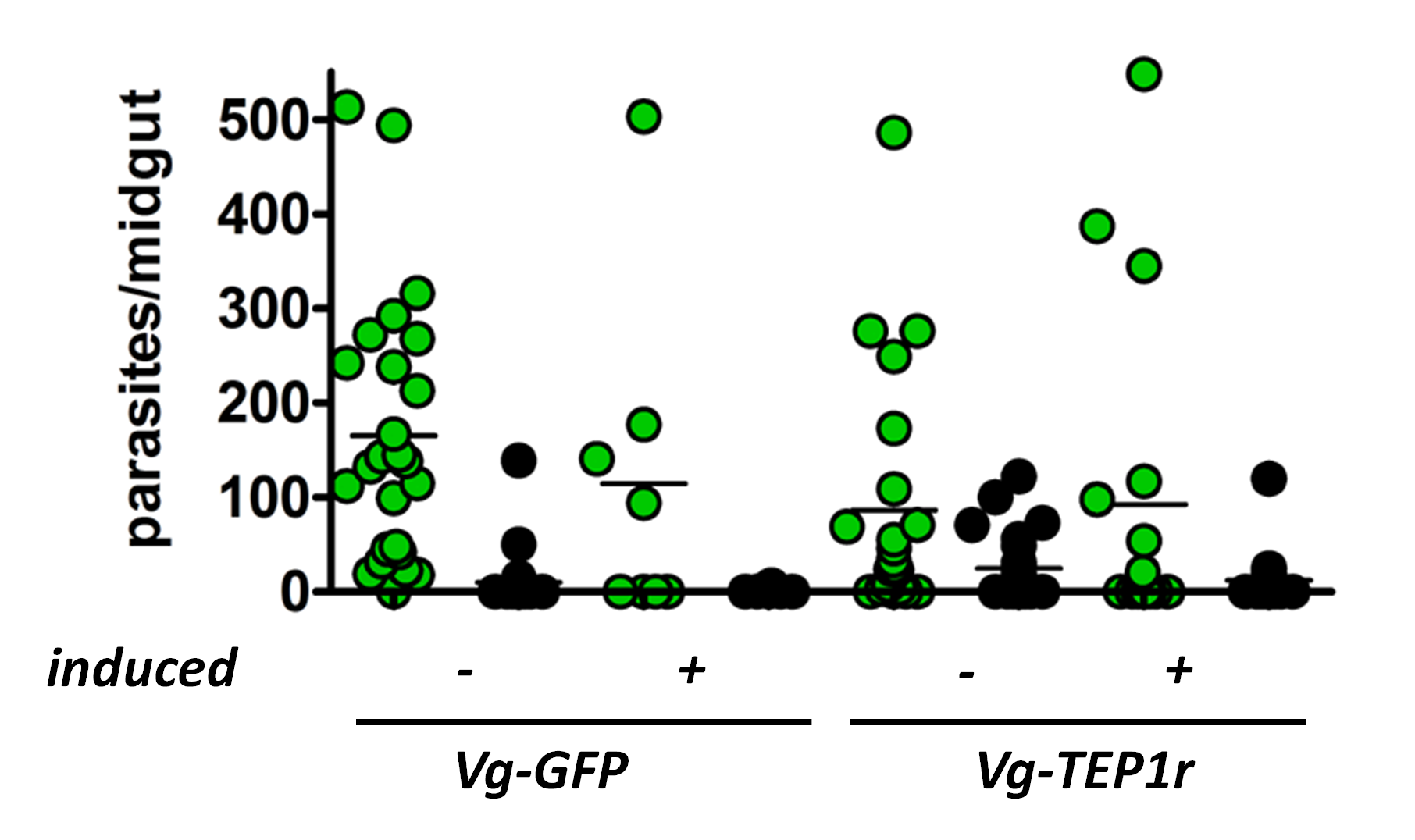

Supplement: S5 Fig — Compared are transgenic mosquitoes expressing TEP1r or GFP under the Vg promoter. For each mosquito group the un-induced (no prior blood meal) and the induced (blood fed on a non infected mouse 3 days before infection) are compared. Shown are live parasites (green circles) and melanized parasites (black circles) in the midguts of blood fed mosquitoes 7 days after infection. Infection data is given in S2 Table. (TIF) [file ppat.1006113.s009.tif]

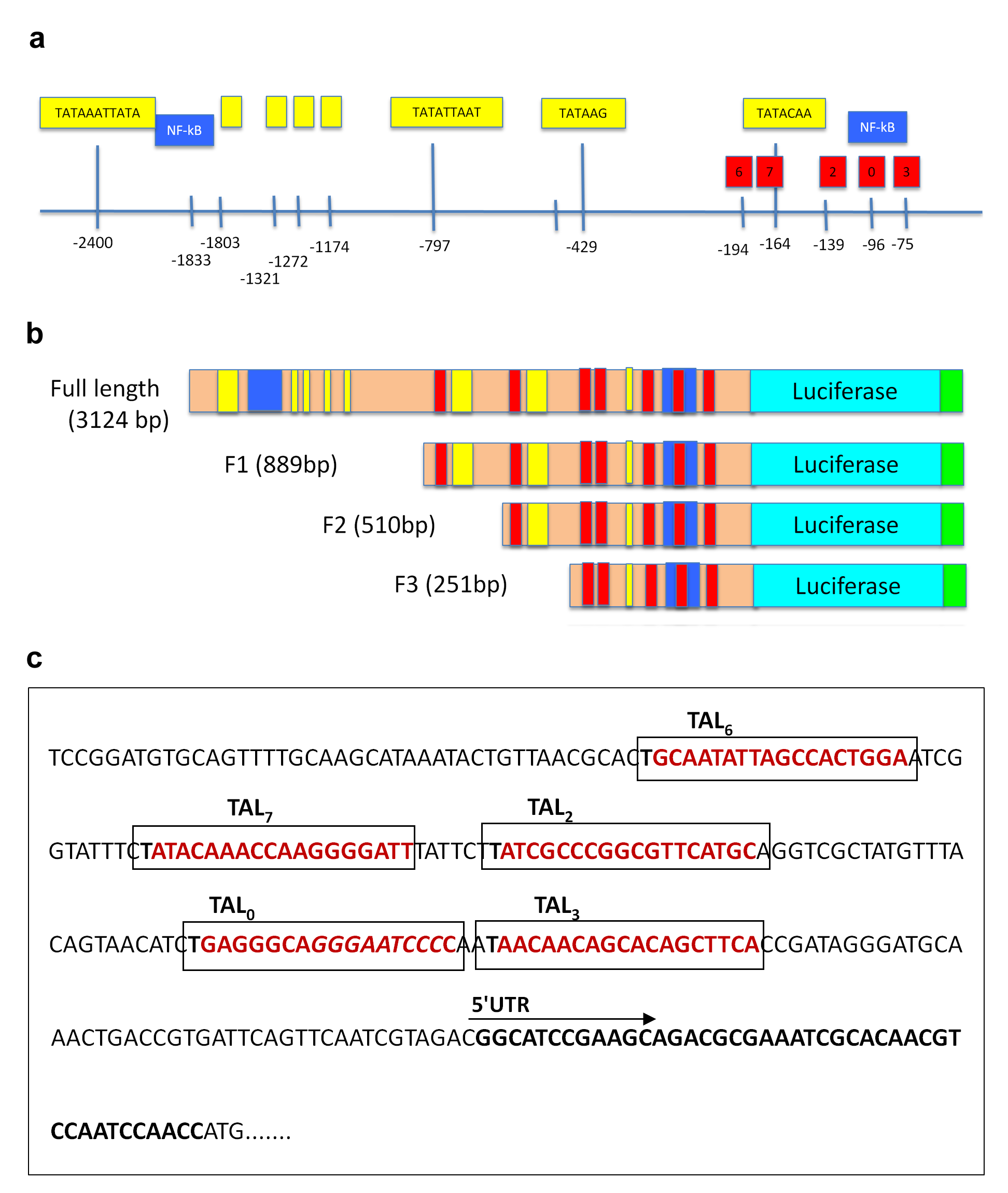

Supplement: S6 Fig — a. Schematic representation of the 3 kb promoter fragment, indicating the position of potential TATA boxes (yellow), potential NF-κB binding sites (blue) and TALE binding sites (red). Drawing not to scale. b. Four TEP1 promoter fragments fused to a luciferase reporter gene. The position of potential TATA boxes (yellow), potential NF-κB binding sites (blue), TALE binding sites (red), Luciferase gene (light blue) and SV40 term (green) is shown. c. Sequence of the 251bp minimal promoter with TALE binding sites (red) and transcription start site (determined by 5'RACE) indicated by an arrow and 5'UTR. The GGGAATCCC NF-κB binding site is in the TAL0 recognition site indicated by Italic. (TIF) [file ppat.1006113.s010.tif]

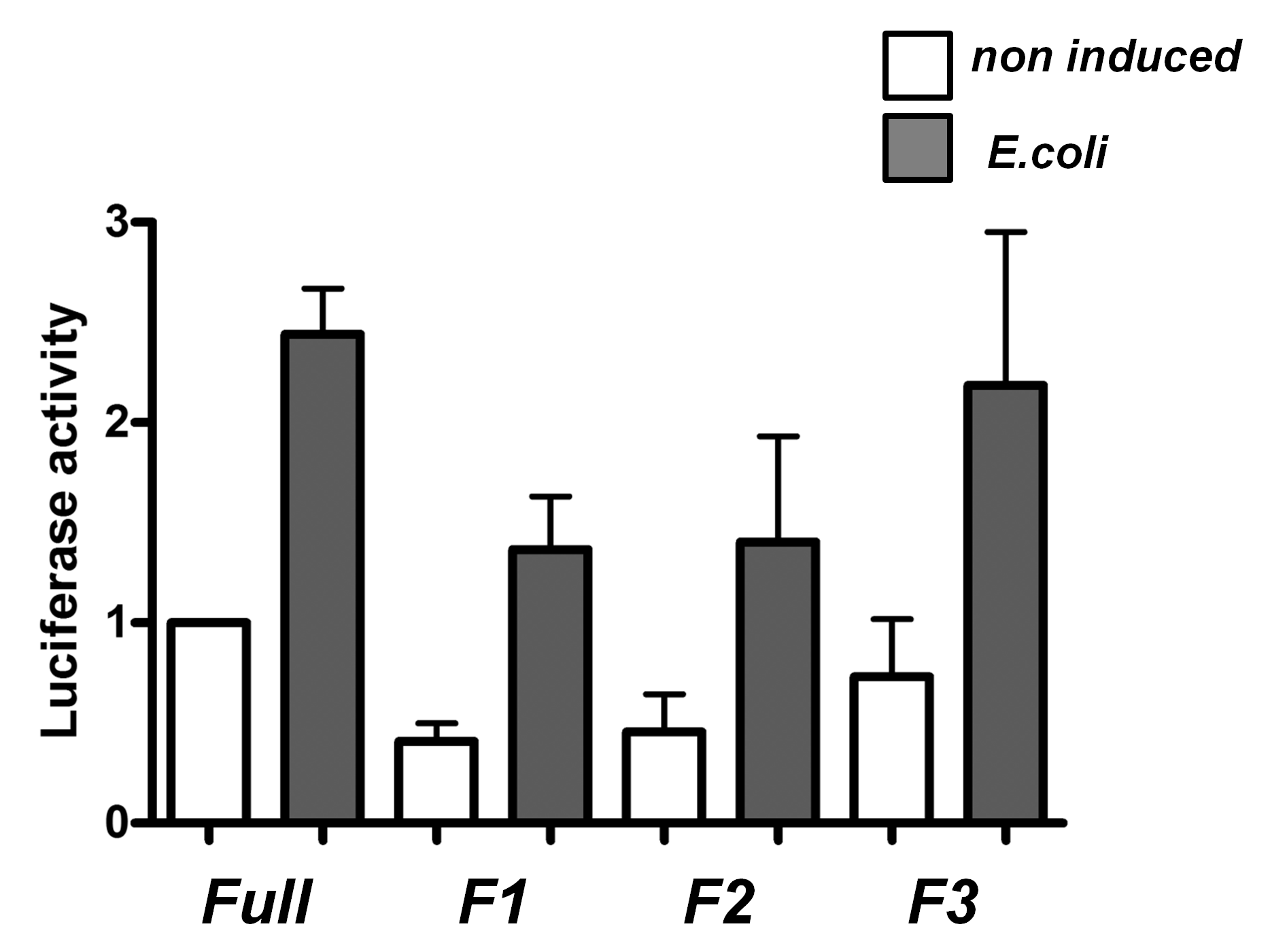

Supplement: S7 Fig — Four different luciferase constructs (described in S3b Fig) were transfected into S2 cells and analyzed for Luciferase activity. For each condition, induction of the NF-κB pathway was triggered by addition of heat killed bacteria to cell medium. Depicted is luciferase activity in non-induced (white) and E. coli challenged (grey) cells. Luciferase activity is normalized to the activity in cells transfected with the full length promoter and uninduced. Shown is the average of three independent experiments. Transfected construct carries firefly luciferase under the control of TEP1 as well as Renilla luciferase under a constitutive promoter which serves as control. Luciferase activity is expressed as firefly luciferase activity divided by Renilla luciferase activity. (TIF) [file ppat.1006113.s011.tif]

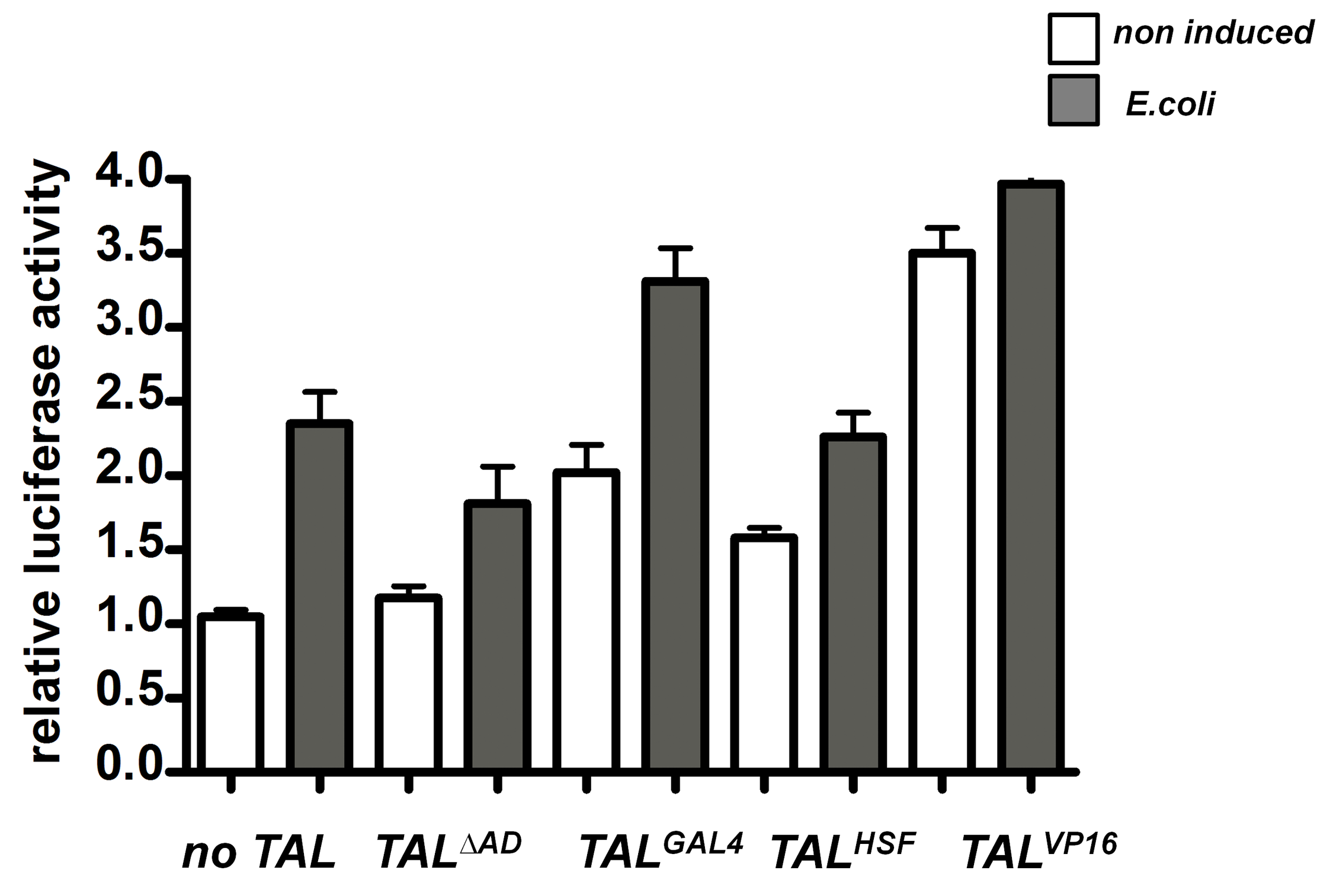

Supplement: S8 Fig — TALEs with the same DNA binding domain (TAL0) but different activation domains were co-transfected into S2 cells together with the Luciferase reporter under the control of the minimal TEP1 promoter fragment. Control (no TAL) were transfected with GFP, TALΔAD has no activation domain and serves as control, yeast GAL4, heat shock factor 1 transcription (HSF) and Herpes virus VP-16 domains were tested. Depicted is luciferase activity in uninduced (white) and induced (grey) cells. Luciferase activity is normalized to the activity in cells transfected with GFP and uninduced. Shown is a representative of at least 3 independent experiments. Error bars depict standard deviation from the mean of three biological repetitions. (TIF) [file ppat.1006113.s012.tif]

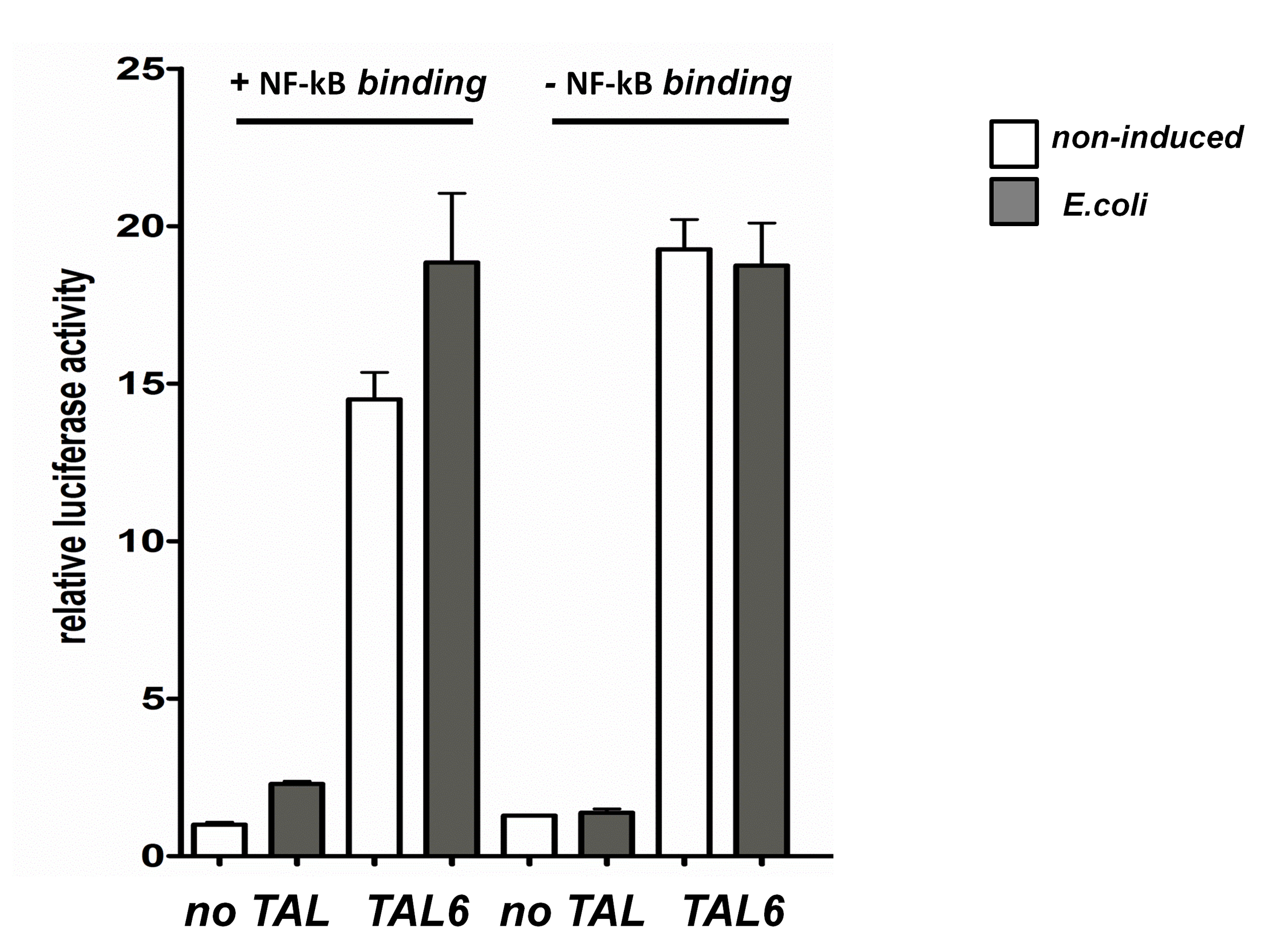

Supplement: S9 Fig — S2 cells were transfected with two different reporters, one with a 250bp fragment of TEP1 promoter carrying an NF-κB binding site, or with the same promoter fragment where the NF-κB binding site was mutated (GGGAATCCCC to CGGAATACCG). Together with the reporter, cells were transfected either with a plasmid encoding GFP (no TAL) or a TAL6-VP16 construct. Depicted is luciferase activity with (grey) or without (white) E. coli challenge. Luciferase activity is normalized to relative to the activity in cells transfected with GFP and without E. coli. Shown is a representative of at least 3 independent experiments. Error bars depict standard deviation from the mean of three biological repetitions. (TIF) [file ppat.1006113.s013.tif]
