# Supplementary material for: Transgenic Expression of the Anti-parasitic Factor TEP1 in the Malaria Mosquito Anopheles gambiae
Source: PLoS Pathog. 2017 Jan 17;13(1):e1006113. doi: 10.1371/journal.ppat.1006113 (PMC5240933; doi:10.1371/journal.ppat.1006113)
Supplement: S1 File — (DOCX) [file ppat.1006113.s014.docx]

**Supplementary file**

***SPCLIP1* silencing abolishes *P. berghei* melanization in *TEP1∆T; Vg-TEP1r* mosquitoes**

*TEP1∆T* mutant mosquitoes rescued with the *Vg-TEP1r* transgene were injected with double-stranded RNA corresponding to the *LacZ* sequence (negative control) or to *SPCLIP1* [39, 62]*.* On day 3 following dsRNA injection, mosquitoes were offered a *P. berghei* infected mouse. The following tables show the number of live and melanized parasites counted in each midgut of mosquitoes dissected 7 days after infection. The graphs following the tables show parasite counts without associating live and melanized parasites for a given midgut, and statistical significance analyzed with a Mann-Whitney test.

**Experiment 1**

| ds*LacZ* | | | ds*SPCLIP1* | | |
| --- | --- | --- | --- | --- | --- |
| Gut # | Number of live parasites | Number of melanized parasites | Gut # | Number of live parasites | Number of melanized parasites |
| 1 | 531 | 0 | 1 | 898 | 0 |
| 2 | 506 | 0 | 2 | 887 | 0 |
| 3 | 347 | 0 | 3 | 736 | 0 |
| 4 | 318 | 0 | 4 | 707 | 0 |
| 5 | 152 | 235 | 5 | 593 | 0 |
| 6 | 117 | 0 | 6 | 590 | 0 |
| 7 | 108 | 0 | 7 | 492 | 0 |
| 8 | 91 | 0 | 8 | 393 | 0 |
| 9 | 85 | 9 | 9 | 253 | 0 |
| 10 | 65 | 0 | 10 | 218 | 0 |
| 11 | 21 | 44 | 11 | 147 | 0 |
| 12 | 15 | 86 | 12 | 115 | 0 |
| 13 | 6 | 71 | 13 | 85 | 0 |
| 14 | 6 | 140 | 14 | 68 | 0 |
| 15 | 4 | 35 |  |  |  |
| 16 | 2 | 278 |  |  |  |
| 17 | 1 | 0 |  |  |  |
| 18 | 0 | 5 |  |  |  |

**Experiment 2**

| ds*LacZ* | | | ds*SPCLIP1* | | |
| --- | --- | --- | --- | --- | --- |
| Gut # | Number of live parasites | Number of melanized parasites | Gut # | Number of live parasites | Number of melanized parasites |
| 1 | 258 | 0 | 1 | 560 | 0 |
| 2 | 240 | 0 | 2 | 520 | 0 |
| 3 | 57 | 0 | 3 | 354 | 0 |
| 4 | 36 | 40 | 4 | 283 | 0 |
| 5 | 34 | 0 | 5 | 176 | 0 |
| 6 | 34 | 25 | 6 | 111 | 0 |
| 7 | 14 | 0 | 7 | 110 | 0 |
| 8 | 12 | 52 | 8 | 85 | 0 |
| 9 | 8 | 20 | 9 | 48 | 0 |
| 10 | 6 | 27 | 10 | 34 | 0 |
| 11 | 5 | 0 | 11 | 26 | 0 |
| 12 | 3 | 0 | 12 | 12 | 0 |
| 13 | 2 | 0 | 13 | 11 | 0 |
| 14 | 0 | 0 | 14 | 10 | 0 |
| 15 | 0 | 0 | 15 | 8 | 0 |
|  |  |  | 16 | 6 | 0 |
|  |  |  | 17 | 4 | 0 |
|  |  |  | 18 | 2 | 0 |
|  |  |  | 19 | 1 | 0 |
|  |  |  | 20 | 0 | 0 |
|  |  |  | 21 | 0 | 0 |
|  |  |  | 22 | 0 | 0 |
|  |  |  | 23 | 0 | 0 |
|  |  |  | 24 | 0 | 0 |
|  |  |  | 25 | 0 | 0 |
