# Supplementary material for: Transgenic Expression of the Anti-parasitic Factor TEP1 in the Malaria Mosquito Anopheles gambiae
Source: PLoS Pathog. 2017 Jan 17;13(1):e1006113. doi: 10.1371/journal.ppat.1006113 (PMC5240933; doi:10.1371/journal.ppat.1006113)
Supplement: S1 Text — Complete sequences of plasmids, as well as promoters and TALE domains that were used in this study. (PDF) [file ppat.1006113.s015.pdf]

Plasmid sequences

pDSAP-LRIM1-GFP

```
LOCUS       pDSAPLrim1-GFP          7202 bp    DNA     circular       22-JUN-
     2016
FEATURES             Location/Qualifiers
     misc_feature    complement(5317..5412)
                     /note="attL2"
     misc_feature    complement(5463..5481)
                     /note="M13R"
     misc_feature    3718..3767
                     /note="3xP3 element"
     misc_feature    complement(337..459)
                     /note="attR4"
     misc_feature    271..286
                     /note="M13F"
     misc_feature    3141..3367
                     /note="SV40 term"
     misc_feature    3368..3644
                     /note="attB"
     misc_feature    3860..4508
                     /note="OpIE2 promoter"
     misc_feature    4509..5110
                     /note="pac puromycin resistance ORF"
     misc_feature    5110..5269
                     /note="SV40 terminator"
     misc_feature    complement(5507..5525)
                     /note="pDONR-RP"
     misc_feature    1455..1467
                     /note=""
     misc_feature    2375..2409
                     /note="UTR"
     misc_feature    468..2374
                     /note="Lrim1 promoter region"
     misc_feature    2416..3135
                     /note="GFP"
     source          1..7202
                     /dnas_title="pDSAPLrim1-GFP"
ORIGIN
        1 CTTTCCTGCG TTATCCCCTG ATTCTGTGGA TAACCGTATT ACCGCCTTTG AGTGAGCTGA
       61 TACCGCTCGC CGCAGCCGAA CGACCGAGCG CAGCGAGTCA GTGAGCGAGG AAGCGGAAGA
      121 GCGCCCAATA CGCAAACCGC CTCTCCCCGC GCGTTGGCCG ATTCATTAAT GCAGCTGGCA
      181 CGACAGGTTT CCCGACTGGA AAGCGGGCAG TGAGCGCAAC GCAATTAATA CGCGTACCGC
      241 TAGCATGGAT GTTTTCCCAG TCACGACGTT GTAAAACGAC GGCCAGTCTT AAGCTCGGGC
      301 CCCTACAGGT CACTAATACC ATCTAAGTAG TTGATTCATA GTGACTGGAT ATGTTGTGTT
      361 TTACAGTATT ATGTAGTCTG TTTTTTATGC AAAATCTAAT TTAATATATT GATATTTATA
      421 TCATTTTACG TTTCTCGTTC AACTTTTCTA TACAAAGTTg gtACcggATC CCGGGCACGC
      481 TCAACAGTGC CTGAGAAGCA ATGAAAATTA ATTTCCATTT AAAGACGTGC TGTTTATGTT
      541 TTTTTTATAA TGGCGGTTGA TTGAACCCGA ACAAAACTTG ACTTTGTGCT TTTGTACGTA
      601 AGCTGCAAGG TTGGTGATGT GGTTGGTGGT GATAAAAAGA CCAAAATCTC CGATGCCAGT
      661 GATAGTTTCG CTTCGAATGG GTTAGATTCT GTGAACACAT GTGAGGAAAT GGTTGTTCAA
      721 TTTTTTTATG ACATCTTGGC TGTAACGTTC TCCAGCTGCT GGTATGAGAC GCGAACAATC
      781 GGTTTGCTTG TTTGGCGCAC CCATTACACA TCTGGCTGCG TGAACCGCAC ATGTGGTATC
      841 ATTAAGTGGC GATGACTGTG AAATTGTGAA TGAGCTGATA GTGACAACAA AAAAATCCAA
```

901 CAGTTTAATA GTTTCTTCTC GATTTGATCG TAAAACAACT ACAAAAAAAA TCCCAACTGC
961 TGACTGTGCC CATTAGTGTC GTGCTTACTG TGGAGAAAAA AAAAAGCGAA CGCCAAGCAC
1021 AACGCAGTCA TTGTCTCGGT CCCTGTGGTC TTAAATTGAT TTTTATTTGT TTTTGGGACG
1081 TACGAAAGTG TGTTGTGAAC GGCGTGAAGT ATTTATTGTC GCGGTCGGTA TGGCAGGCCA
1141 ACGCACCAAA CAACAAGACA CCCGCACACA TTGACGTTAA TATTTATGTT TGTTCATAAA
1201 ATTATTAACC GATCGTTACC CCTACCGGGC ACGGTGCTGG CTCGAGGTAG GTTCGAAGCG
1261 TTCAAACCAA CGATTTTGAC TGTCTTGGTC GAAAACGTGC TAGCGCCAGC ATGGTCACCG
1321 TACACAGaat cggtgcgttt cgcgtttttg ttgacctgga tttggtctta tttttgtcac
1381 tttgttaact gcgagcagtg accgcggttg tacgacacgg tcaacagcgt ggtCGGATGC
1441 AGTAGCAGCA TCACCAATAC ACCCGTActt aaacatgcga gcacaaacat tgctatcatg
1501 ctgaacgttt gatgttaaaa cgcgtgtgac agcagaagaa tggttcgctg tgtccgcggc
1561 tgctagcgat cgtaccacaa tcgaccgtaa agtgcatcgt gcagctaatt agtcacagca
1621 caggttcgaa tggcacagca gcaggtttga cacctctggt ttgcgaatga aattatcaga
1681 aggaaattgc cagcttgtgc tagtgttaca tgaatcatgt ggttcatatt tccatgacta
1741 attgacaagg tagtattcca ccgtgttgga ttctgttcat ctgctcggta cgggttgttg
1801 tacttttaaa acatttattt aaatatgtat gtatttaaaa aatatggcga agttcatgtt
1861 ttacattatt ataaacattg gacctgaaat aagtaatttt tccatttaat aaaaaaaatt
1921 gtgttaaatt gcaagttatc actcaaatga tattgtgttc ttatcgaaat attttaatgt
1981 aataactaca ttaaagataa aaattctaat atcttcatta ctgaaagaaa aattgaatgt
2041 catattggtg gtaactgtta ctctttgttt taaaataatt attttccaag aaaaatatca
2101 cattttagtc ttcagttact ttgaatttta gcacaagaaa atcgttaata ccacaagaat
2161 cggtacaatt atttttacat ttctgtaaaa aaagtatttc gaccaaacgc taccttccgt
2221 taagaaacgt ttcaaagatt caAGCTCTAG GGGATTGCAT CCgaagtcaa ccaaaacatg
2281 accccgccgg taccgcttat caggaggaaa tccccggcgg gcgaagctaa tcactggcga
2341 ggcatggcat ctgcaccatc gctgtcagta gtgaGTGAAG CGCGATCGAG TAAGAACGCG
2401 AAACGAAAGA CCACCATGGT GAGCAAGGGC GAGGAGCTGT TCACCGGGGT GGTGCCCATC
2461 CTGGTCGAGC TGGACGGCGA CGTAAACGGC CACAAGTTCA GCGTGTCCGG CGAGGGCGAG
2521 GGCGATGCCA CCTACGGCAA GCTGACCCTG AAGTTCATCT GCACCACCGG CAAGCTGCCC
2581 GTGCCCTGGC CCACCCTCGT GACCACCCTG ACCTACGGCG TGCAGTGCTT CAGCCGCTAC
2641 CCCGACCACA TGAAGCAGCA CGACTTCTTC AAGTCCGCCA TGCCCGAAGG CTACGTCCAG
2701 GAGCGCACCA TCTTCTTCAA GGACGACGGC AACTACAAGA CCCGCGCCGA GGTGAAGTTC
2761 GAGGGCGACA CCCTGGTGAA CCGCATCGAG CTGAAGGGCA TCGACTTCAA GGAGGACGGC
2821 AACATCCTGG GGCACAAGCT GGAGTACAAC TACAACAGCC ACAACGTCTA TATCATGGCC
2881 GacAAGCAGA AGAACGGCAT CAAGGTGAAC TTCAAGATCC GCCACAACAT CGAGGaCGGC
2941 AGCGTGCAGC TCGCCGACCA CTACCAGCAG AACACCCCCA TCGGCGacGG CCCCGTGCTG
3001 CTGCCCGaCA ACCACTACCT GAGCACCCAG TCCGCCCTGA GCAAagaCCC CAACGAgaag
3061 CGCGATCACA TGGTCCTGCT GgaGTTCGTG ACCGCCGCCG GgatCacTCT CGGCATGGAC
3121 GAGCTGTACA AGTAAgcttC TAGACATAAT CAGCCATACC ACATTTGTAG AGGTTTTACT
3181 TGCTTTAAAA AACCTCCCAC ACCTCCCCCT GAACCTGAAA CATAAAATGA ATGCAATTGT
3241 TGTTGTTAAC TTGTTTATTG CAGCTTATAA TGGTTACAAA TAAAGCAATA GCATCACAAA
3301 TTTCACAAAT AAAGCATTTT TCTTCACTGC ATTCTAGTTG TGGTTTGTCC AAACTCATCA
3361 ATGTATCtcg aCGATGTAGG TCACaGTCTC GAAGCCGCGG TGCGGGTGCC AGGGCGTGCC
3421 CTTGGGCTCC CCGGGCGCGT ACTCCACCTC ACCCATCTGG TCCATCATGA TGAACGGGTC
3481 GAGGTGGCGG TAGTTGATCC CGGCGAACGC GCGGCGCACC GGGAAGCCCT CGCCCTCGAA
3541 ACCGCTGGGC GCGGTGGTCA CGGTGAGCAC GGGACGTGCG ACGGCGTCGG CGGGTGCGGA
3601 TACGCGGGGC AGCGTCAGCG GGTTCTCGAC GGTCACGGCG GGCAattCCT GCAGACTTCC
3661 GGTATCTCGC GTTTGTTTGA TCGCACGGTT CCCACAATGG TTAATTCGAG CTCGCCCGGG
3721 GATCTAATTC AATTAGAGAC TAATTCAATT AGAGCTAATT CAATTAGGAT CCAAGCTTAT
3781 CGATTTCGAT AAGCTTGATA TCGAATTCCT GCAGCCCGGG GGATCCActa gtggatcccc
3841 cgggctgcag gaattcgatC ATGATGATAA ACAATGTATG GTGCTAATGT TGCTTCAACA
3901 ACAATTCTGT TGAACTGTGT TTTCATGTTT GCCAACAAGC ACCTTTATAC TCGGTGGCCT
3961 CCCCACCACC AACTTTTTTG CACTGCAAAA AAACACGCTT TTGCACGCGG GCCCATACAT
4021 AGTACAAACT CTACGTTTCG TAGACTATTT TACATAAATA GTCTACACCG TTGTATACGC
4081 TCCAAATACA CTACCACACA TTGAACCTTT TTGCAGTGCA AAAAAGTACG TGTCGGCAGT
4141 CACGTAGGCC GGCCTTATCG GGTCGCGTCC TGTCACGTAC GAATCACATT ATCGGACCGG
4201 ACGAGTGTTG TCTTATCGTG ACAGGACGCC AGCTTCCTGT GTTGCTAACC GCAGCCGGAC
4261 GCAACTCCTT ATCGGAACAG GACGCGCCTC CATATCAGCC GCGCGTTATC TCATGCGCGT

```
     4321 GACCGGACAC GAGGCGCCCG TCCCGCTTAT CGCGCCTATA AATACAGCCC GCAACGATCT
     4381 GGTAAACACA GTTGAACAGC ATCTGTTCGA ATTAATTCgG GATCTgccgg gCTGCAGCAC
     4441 GTGTTGACAA TTAATCATCG GCATAGTATA TCGGCATAGT ATAATACGAC TCACTATAGG
     4501 AGGGCCACcA TGaccgagta caagcccacg gtgcgcctcg ccacccgcga cgacgtcccc
     4561 Agggccgtac gcaccctcgc cgccgcgttc gccgactacc ccgccacgcg ccacaccgtc
     4621 gaTccggacc gccacatcga gcgggtcacc gagctgcaag aactcttcct cacgcgcgtc
     4681 gggctcgaca tcggcaaggt gtgggtcgcg gacgacggcg ccgcggtggc ggtctggacc
     4741 acgccggaga gcgtcgaagc gggggcggtg ttcgccgaga tcggcccgcg catggccgag
     4801 ttgagcggtt cccggctggc cgcgcagcaa cagatggaag gcctcctggc gccgcaccgg
     4861 cccaaggagc ccgcgtggtt cctggccacc gtcggcgtct cgcccgacca ccagggcaag
     4921 ggtctgggca gcgccgtcgt gctccccgga gtggaggcgg ccgagcgcgc cggggtgccc
     4981 gccttcctgg agacctccgc gccccgcaac ctccccttct acgagcggct cggcttcacc
     5041 gtcaccgccg acgtcgagGt gcccgaagga ccgcgcacct ggtgcatgac ccgcaagccc
     5101 ggtgcctgaC TCGACCTCGA AACTTGTTTA TTGCAGCTTA TAATGGTTAC AAATAAAGCA
     5161 ATAGCATCAC AAATTTCACA AATAAAGCAT TTTTTTCACT GCATTCTAGT TGTGGTTTGT
     5221 CCAAACTCAT CAATGTATCT TATCATGTCT ggatcatggg gggggcccgG TACGGCGCGC
     5281 CTAGAGCGGC CGCCACCGCG GTGGAGCTCG AGTACCCAGC TTTCTTGTAC AAAGTTGGCA
     5341 TTATAAGAAA GCATTGCTTA TCAATTTGTT GCAACGAACA GGTCACTATC AGTCAAAATA
     5401 AAATCATTAT TTGCCATCCA GCTGCAGGGC GGCCGCGATA TCCCCTATAG TGAGTCGTAT
     5461 TACATGGTCA TAGCTGTTTC CTGGCAGCTC TGGCCCGTGT CTCAAAATCT CTGATGTTAC
     5521 ATTGCACAAG ATAAAAATAT ATCATCATGA ACAATAAAAC TGTCTGCTTA CATAAACAGT
     5581 AATACAAGGG GTGTTATGAG CCATATTCAA CGGGAAACGT CGAGGCCGCG ATTAAATTCC
     5641 AACATGGATG CTGATTTATA TGGGTATAAA TGGGCTCGCG ATAATGTCGG GCAATCAGGT
     5701 GCGACAATCT ATCGCTTGTA TGGGAAGCCC GATGCGCCAG AGTTGTTTCT GAAACATGGC
     5761 AAAGGTAGCG TTGCCAATGA TGTTACAGAT GAGATGGTCA GACTAAACTG GCTGACGGAA
     5821 TTTATGCCTC TTCCGACCAT CAAGCATTTT ATCCGTACTC CTGATGATGC ATGGTTACTC
     5881 ACCACTGCGA TCCCCGGAAA AACAGCATTC CAGGTATTAG AAGAATATCC TGATTCAGGT
     5941 GAAAATATTG TTGATGCGCT GGCAGTGTTC CTGCGCCGGT TGCATTCGAT TCCTGTTTGT
     6001 AATTGTCCTT TTAACAGCGA TCGCGTATTT CGTCTCGCTC AGGCGCAATC ACGAATGAAT
     6061 AACGGTTTGG TTGATGCGAG TGATTTTGAT GACGAGCGTA ATGGCTGGCC TGTTGAACAA
     6121 GTCTGGAAAG AAATGCATAA ACTTTTGCCA TTCTCACCGG ATTCAGTCGT CACTCATGGT
     6181 GATTTCTCAC TTGATAACCT TATTTTTGAC GAGGGGAAAT TAATAGGTTG TATTGATGTT
     6241 GGACGAGTCG GAATCGCAGA CCGATACCAG GATCTTGCCA TCCTATGGAA CTGCCTCGGT
     6301 GAGTTTTCTC CTTCATTACA GAAACGGCTT TTTCAAAAAT ATGGTATTGA TAATCCTGAT
     6361 ATGAATAAAT TGCAGTTTCA TTTGATGCTC GATGAGTTTT TCTAATCAGA ATTGGTTAAT
     6421 TGGTTGTAAC ACTGGCAGAG CATTACGCTG ACTTGACGGG ACGGCGCAAG CTCATGACCA
     6481 AAATCCCTTA ACGTGAGTTA CGCGTCGTTC CACTGAGCGT CAGACCCCGT AGAAAAGATC
     6541 AAAGGATCTT CTTGAGATCC TTTTTTTCTG CGCGTAATCT GCTGCTTGCA AACAAAAAAA
     6601 CCACCGCTAC CAGCGGTGGT TTGTTTGCCG GATCAAGAGC TACCAACTCT TTTTCCGAAG
     6661 GTAACTGGCT TCAGCAGAGC GCAGATACCA AATACTGTTC TTCTAGTGTA GCCGTAGTTA
     6721 GGCCACCACT TCAAGAACTC TGTAGCACCG CCTACATACC TCGCTCTGCT AATCCTGTTA
     6781 CCAGTGGCTG CTGCCAGTGG CGATAAGTCG TGTCTTACCG GGTTGGACTC AAGACGATAG
     6841 TTACCGGATA AGGCGCAGCG GTCGGGCTGA ACGGGGGGTT CGTGCACACA GCCCAGCTTG
     6901 GAGCGAACGA CCTACACCGA ACTGAGATAC CTACAGCGTG AGCTATGAGA AAGCGCCACG
     6961 CTTCCCGAAG GGAGAAAGGC GGACAGGTAT CCGGTAAGCG GCAGGGTCGG AACAGGAGAG
     7021 CGCACGAGGG AGCTTCCAGG GGGAAACGCC TGGTATCTTT ATAGTCCTGT CGGGTTTCGC
     7081 CACCTCTGAC TTGAGCGTCG ATTTTTGTGA TGCTCGTCAG GGGGGCGGAG CCTATGGAAA
     7141 AACGCCAGCA ACGCGGCCTT TTTACGGTTC CTGGCCTTTT GCTGGCCTTT TGCTCACATG
     7201 TT
//
```

pDSAT-APL1-GFP

```
LOCUS       pDSAT                   7006 bp    DNA     circular      22-JUN-
    2016
```

```
FEATURES             Location/Qualifiers
     misc_feature    complement(5121..5216)
                     /note="attL2"
     misc_feature    complement(5267..5285)
                     /note="M13R"
     misc_feature    3831..3880
                     /note="3xP3 element"
     misc_feature    3920..3927
                     /note="TATA"
     misc_feature    complement(337..459)
                     /note="attR4"
     misc_feature    271..286
                     /note="M13F"
     misc_feature    3254..3480
                     /note="SV40 term"
     misc_feature    3481..3757
                     /note="attB"
     misc_feature    4836..5057
                     /note="SV40 term"
     misc_feature    4091..4807
                     /note="mTurquoise2"
     misc_feature    complement(5311..5329)
                     /note="DONR-RP"
     misc_feature    468..2485
                     /note="APL1c Promoter region"
     misc_feature    2465..2485
                     /note="Race UTR"
     misc_feature    2486..2518
                     /note="UTR"
     misc_feature    2465..2518
                     /note="Exon1"
     misc_feature    complement(2523..3225)
                     /note="GFP"
     source          1..7006
                     /dnas_title="pDSAT APL1_GFP"
ORIGIN
        1 CTTTCCTGCG TTATCCCCTG ATTCTGTGGA TAACCGTATT ACCGCCTTTG AGTGAGCTGA
       61 TACCGCTCGC CGCAGCCGAA CGACCGAGCG CAGCGAGTCA GTGAGCGAGG AAGCGGAAGA
      121 GCGCCCAATA CGCAAACCGC CTCTCCCCGC GCGTTGGCCG ATTCATTAAT GCAGCTGGCA
      181 CGACAGGTTT CCCGACTGGA AAGCGGGCAG TGAGCGCAAC GCAATTAATA CGCGTACCGC
      241 TAGCATGGAT GTTTTCCCAG TCACGACGTT GTAAAACGAC GGCCAGTCTT AAGCTCGGGC
      301 CCCTACAGGT CACTAATACC ATCTAAGTAG TTGATTCATA GTGACTGGAT ATGTTGTGTT
      361 TTACAGTATT ATGTAGTCTG TTTTTTATGC AAAATCTAAT TTAATATATT GATATTTATA
      421 TCATTTTACG TTTCTCGTTC AACTTTTCTA TACAAAGTTg gtACcggATC Ccttctggtc
      481 tgtgtgcgag ggttattagg aaggtgatta ggattaggaa gggaacatta aacaAttatt
      541 tatttaattt attttctatg acaagcaact gtaaaataaa gtagcataat tgtagcacaa
      601 ttagtatata aaaaattaat tatatcaatt gctacgtcaa tgatctagtt aagacactac
      661 gaccggctgg aaggttttta tcgcgcatat ttagtctggg gcagtttagt tataccataa
      721 catccacaac aggtgctgga attattgctc aaaagtcggt ataaattcga cgctagtcgg
      781 aaaagtctcc agaatcaaaa tccggacacA cagtcgtcGg gagtcggtgt cgtcacgagt
      841 tgaagtaggc cgatatctgc gccattcgta gtagaacatc atcgtacagt tgcaatcttc
      901 caaattgaac tgtcgacatt ctctcattgt aacacttttt cttaacaaca gcgccacaat
      961 gactaagtcg cattggtatg aaagatggcc aaaattatgt acatactttt tttaataaag
     1021 ctttgctgat gtatttttaa aacagtgtta caatcaaagc tctgcttgtg taatacacac
     1081 acaaagtgaa gaaagaaaaa caaaaacaaa atgtaatacc ttatcacaaa Cacattgcac
     1141 aggtcgtcta cgactgcgag atcgatcggc tacgtctcgg acgccaatct ccgaactcca
     1201 aaagactctg aacgaatcgg agtaagggtg gcactcgggg ggttccgttt tctccggagc
```

1261 taaaatcaaa tcaaataata gctgcggccc gttcagagac gcggagagtg aaacccatca
1321 ctagtttggg ttgtttgcga accgacgagg tagttttgaa caccgaaccg aaagaaacat
1381 gggcatgtag aagatggtta tcatgcagct attaagcata gcgctttgGa tgaatttctg
1441 tattgataac aaacatctca ggatacactt ttttccattt ccttttttatt catcataccc
1501 aacgtcaacc cgacgatgtc atcaggtatg tgtatattag ctgacattgA atgttggagc
1561 gaaagaacaa gttcgctcac ctgttagata ttgctttgca gatcgctaca aCccagatac
1621 aaccagggtc cgagcatcct Ggacaattat taccgggtca tgccatgacg aagtgctttt
1681 tcgtaagcga cacctgcaac aatgaagcca ttttttctta ttttttgttt ttccatcatg
1741 aaaacacgaa ctggctatta tttctctggt Aaaagatatt taaatctaaa catgaacatc
1801 acatcccaag tttccggtca cccagatgat ggtgaaatgc ttttGcagct tcaaaaaaac
1861 catgctcaat agcaatcaca actaaactgc tcattctgct gctttgggaa tcccggtgct
1921 gcgaacggtt tcgaagtgac ccgcaaagtt caatattgtg catccagcct ctgccgatcg
1981 cacattatca gcattgcgcg agtggaagac ctctcgcaca tctatacaca cgcactCtct
2041 ccctctctct ctttatctct ctcacacGtt ctcggGTGAG CAGCATCGAT CGCACCTCAA
2101 AGCACAATCA AAAACATGCA GTGGTATTTG CTCagaacag ttcgtgctaa tacaagtagg
2161 tttgtcgcac aatcactaca cttttcggaa tctcaaTagt aagagatgtc tGcaaacacc
2221 tgtcccaacc aatGatgttg gatcggtccc agcttgtacg gtcgaccttt gccctcgaaa
2281 atcacaccag actgtagtga gttaaaataa acacaaaatc cggcaaTtgc tgggcAgcgt
2341 tggtgcgaat ttactcaatc attacatttg ttttcatttt caggacgccg ttatcactgg
2401 gaattcccgt aacgaattca tctttcagtg tgctacgcta gttctaccgc aggaatgatt
2461 gctcagtttc aatccatcat tcgagGAAGA AACACAGTTT TCGAGCATTG AGTACCACaA
2521 CCACCACCAT GGTGAGCAAG GGCGAGGAGC TGTTCACCGG GGTGGTGCCC ATCCTGGTCG
2581 AGCTGGACGG CGACGTAAAC GGCCACAAGT TCAGCGTGTC CGGCGAGGGC GAGGGCGATG
2641 CCACCTACGG CAAGCTGACC CTGAAGTTCA TCTGCACCAC CGGCAAGCTG CCCGTGCCCT
2701 GGCCCACCCT CGTGACCACC CTGACCTACG GCGTGCAGTG CTTCAGCCGC TACCCCGACC
2761 ACATGAAGCA GCACGACTTC TTCAAGTCCG CCATGCCCGA AGGCTACGTC CAGGAGCGCA
2821 CCATCTTCTT CAAGGACGAC GGCAACTACA AGACCCGCGC CGAGGTGAAG TTCGAGGGCG
2881 ACACCCTGGT GAACCGCATC GAGCTGAAGG GCATCGACTT CAAGGAGGAC GGCAACATCC
2941 TGGGGCACAA GCTGGAGTAC AACTACAACA GCCACAACGT CTATATCATG GCCGacAAGC
3001 AGAAGAACGG CATCAAGGTG AACTTCAAGA TCCGCCACAA CATCGAGGaC GGCAGCGTGC
3061 AGCTCGCCGA CCACTACCAG CAGAACACCC CCATCGGCGa cGGCCCCGTG CTGCTGCCCG
3121 aCAACCACTA CCTGAGCACC CAGTCCGCCC TGAGCAAaga CCCCAACGAg aagCGCGATC
3181 ACATGGTCCT GCTGgaGTTC GTGACCGCCG CCGGgatCac TCTCGGCATG GACGAGCTGT
3241 ACAAGTAAgc ttCTAGACAT AATCAGCCAT ACCACATTTG TAGAGGTTTT ACTTGCTTTA
3301 AAAAACCTCC CACACCTCCC CCTGAACCTG AAACATAAAA TGAATGCAAT TGTTGTTGTT
3361 AACTTGTTTA TTGCAGCTTA TAATGGTTAC AAATAAAGCA ATAGCATCAC AAATTTCACA
3421 AATAAAGCAT TTTTTCTTCAC TGCATTCTAG TTGTGGTTTG TCCAAACTCA TCAATGTATC
3481 tcgaCGATGT AGGTCACaGT CTCGAAGCCG CGGTGCGGGT GCCAGGGCGT GCCCTTGGGC
3541 TCCCCGGGCG CGTACTCCAC CTCACCCATC TGGTCCATCA TGATGAACGG GTCGAGGTGG
3601 CGGTAGTTGA TCCCGGCGAA CGCGCGGCGC ACCGGGAAGC CCTCGCCCTC GAAACCGCTG
3661 GGCGCGGTGG TCACGGTGAG CACGGGACGT GCGACGGCGT CGGCGGGTGC GGATACGCGG
3721 GGCAGCGTCA GCGGGTTCTC GACGGTCACG GCGGGCAatt CCTGCAGACT TCCGGTATCT
3781 CGCGTTTGTT TGATCGCACG GTTCCCACAA TGGTTAATTC GAGCTCGCCC GGGGATCTAA
3841 TTCAATTAGA GACTAATTCA ATTAGAGCTA ATTCAATTAG GATCCAAGCT TATCGATTTC
3901 GAACCCTCGA CCGCCGGAGT ATAAATAGAG GCGCTTCGTC TACGGAGCGA CAATTCAATT
3961 CAAACAAGCA AAGTGAACAC GTCGCTAAGC GAAAGCTAAG CAAATAAACA AGCGCAGCTG
4021 AACAAGCTAA ACAATCGGGG TACCGCTAGA GTCGACGGTA CCGCGGGCCC GGGATCCACC
4081 GGTCGCCACc atggtgagca agggcgagga gctgttcacc ggggtggtgc ccatcctggt
4141 cgagctggac ggcgacgtaa acggccacaa gttcagcgtg tccggcgagg gcgagggcga
4201 tgccacctac GGCAAGCTGA CCCTGAAGTT Catctgcacc accggcaagc tgcccgtgcc
4261 ctggcccacc ctcgtgacca ccctgtcctg gggcgtgcag tgcttcgccc gctaccccga
4321 ccacatgaag cagcacgact tcttcaagtc cgccatgccc gaaggctacg tccaggagcg
4381 caccatcttc ttcaaggacg acggcaacta caagacccgc gccgaggtga agttcgaggg
4441 cgacaccctg gtgaaccgca tcgagctgaa gggcatcgac ttcaaggagg acggcaacat
4501 cctggggcac aagctggagt acaactacTt Tagcgacaac gtctatatca ccgccgacaa
4561 gcagaagaac ggcatcaagg ccaacttcaa gatccgccac aacatcgagg acggcggcgt
4621 gcagctcgcc gaccactacc agcagaacac ccccatcggc gacggccccg tgctgctgcc

```
     4681 cgacaaccac tacctgagca cccagtccaa gctgagcaaa gaccccaacg agaagcgcga
     4741 tcacatggtc ctgctggagt tcgtgaccgc cgccgggatc actctcggca tggacgagct
     4801 gtacaagtcc ggaTGAtaga tctgacgggt gatcaAATCA GCCATACCAC ATTTGTAGAG
     4861 GTTTTACTTG CTTTAAAAAA CCTCCCACAC CTCCCCCTGA ACCTGAAACA TAAAATGAAT
     4921 GCAATTGTTG TTGTTAACTT GTTTATTGCA GCTTATAATG GTTACAAATA AAGCAATAGC
     4981 ATCACAAATT TCACAAATAA AGCATTTTTT TCACTGCATT CTAGTTGTGG TTTGTCCAAA
     5041 CTCATCAATG TATCTTAAAG CTTATCGATA CGCGTACGGC GCGCCTAGAG CGGCCGCCAC
     5101 CGCGGTGGAG CTCGAGTACC CAGCTTTCTT GTACAAAGTT GGCATTATAA GAAAGCATTG
     5161 CTTATCAATT TGTTGCAACG AACAGGTCAC TATCAGTCAA AATAAAATCA TTATTTGCCA
     5221 TCCAGCTGCA GGGCGGCCGC GATATCCCCT ATAGTGAGTC GTATTACATG GTCATAGCTG
     5281 TTTCCTGGCA GCTCTGGCCC GTGTCTCAAA ATCTCTGATG TTACATTGCA CAAGATAAAA
     5341 ATATATCATC ATGAACAATA AAACTGTCTG CTTACATAAA CAGTAATACA AGGGGTGTTA
     5401 TGAGCCATAT TCAACGGGAA ACGTCGAGGC CGCGATTAAA TTCCAACATG GATGCTGATT
     5461 TATATGGGTA TAAATGGGCT CGCGATAATG TCGGGCAATC AGGTGCGACA ATCTATCGCT
     5521 TGTATGGGAA GCCCGATGCG CCAGAGTTGT TTCTGAAACA TGGCAAAGGT AGCGTTGCCA
     5581 ATGATGTTAC AGATGAGATG GTCAGACTAA ACTGGCTGAC GGAATTTATG CCTCTTCCGA
     5641 CCATCAAGCA TTTTATCCGT ACTCCTGATG ATGCATGGTT ACTCACCACT GCGATCCCCG
     5701 GAAAAACAGC ATTCCAGGTA TTAGAAGAAT ATCCTGATTC AGGTGAAAAT ATTGTTGATG
     5761 CGCTGGCAGT GTTCCTGCGC CGGTTGCATT CGATTCCTGT TTGTAATTGT CCTTTTAACA
     5821 GCGATCGCGT ATTTCGTCTC GCTCAGGCGC AATCACGAAT GAATAACGGT TTGGTTGATG
     5881 CGAGTGATTT TGATGACGAG CGTAATGGCT GGCCTGTTGA ACAAGTCTGG AAAGAAATGC
     5941 ATAAACTTTT GCCATTCTCA CCGGATTCAG TCGTCACTCA TGGTGATTTC TCACTTGATA
     6001 ACCTTATTTT TGACGAGGGG AAATTAATAG GTTGTATTGA TGTTGGACGA GTCGGAATCG
     6061 CAGACCGATA CCAGGATCTT GCCATCCTAT GGAACTGCCT CGGTGAGTTT TCTCCTTCAT
     6121 TACAGAAACG GCTTTTTCAA AAATATGGTA TTGATAATCC TGATATGAAT AAATTGCAGT
     6181 TTCATTTGAT GCTCGATGAG TTTTTCTAAT CAGAATTGGT TAATTGGTTG TAACACTGGC
     6241 AGAGCATTAC GCTGACTTGA CGGGACGGCG CAAGCTCATG ACCAAAATCC CTTAACGTGA
     6301 GTTACGCGTC GTTCCACTGA GCGTCAGACC CCGTAGAAAA GATCAAAGGA TCTTCTTGAG
     6361 ATCCTTTTTT TCTGCGCGTA ATCTGCTGCT TGCAAACAAA AAAACCACCG CTACCAGCGG
     6421 TGGTTTGTTT GCCGGATCAA GAGCTACCAA CTCTTTTTCC GAAGGTAACT GGCTTCAGCA
     6481 GAGCGCAGAT ACCAAATACT GTTCTTCTAG TGTAGCCGTA GTTAGGCCAC CACTTCAAGA
     6541 ACTCTGTAGC ACCGCCTACA TACCTCGCTC TGCTAATCCT GTTACCAGTG GCTGCTGCCA
     6601 GTGGCGATAA GTCGTGTCTT ACCGGGTTGG ACTCAAGACG ATAGTTACCG GATAAGGCGC
     6661 AGCGGTCGGG CTGAACGGGG GGTTCGTGCA CACAGCCCAG CTTGGAGCGA ACGACCTACA
     6721 CCGAACTGAG ATACCTACAG CGTGAGCTAT GAGAAAGCGC CACGCTTCCC GAAGGGAGAA
     6781 AGGCGGACAG GTATCCGGTA AGCGGCAGGG TCGGAACAGG AGAGCGCACG AGGGAGCTTC
     6841 CAGGGGGAAA CGCCTGGTAT CTTTATAGTC CTGTCGGGTT TCGCCACCTC TGACTTGAGC
     6901 GTCGATTTTT GTGATGCTCG TCAGGGGGGC GGAGCCTATG GAAAAACGCC AGCAACGCGG
     6961 CCTTTTTACG GTTCCTGGCC TTTTGCTGGC CTTTTGCTCA CATGTT
//
```

pattB-RfB2 TEP1-GFP,3xP3-RFP (*TEP1-GFP* reporter)

```
LOCUS       attBrf2_TEP1_GFP        8929 bp    DNA     circular       22-JUN-
     2016
FEATURES             Location/Qualifiers
     misc_feature    complement(8288..8308)
                     /note="T7"
     misc_feature    complement(8314..8330)
                     /note=""
     misc_feature    complement(2216..2500)
                     /note="attB"
     misc_feature    2516..2523
                     /note="attR1"
```

```
     misc_feature    2173..2192
                     /note="T3"
     misc_feature    2138..2156
                     /note="M13R"
     misc_feature    6609..6629
                     /note="attB3"
     misc_feature    8244..8267
                     /note="attB2"
     misc_feature    2516..2537
                     /note="attB1"
     misc_feature    complement(5832..6551)
                     /note="GFP"
     misc_feature    2547..2580
                     /note="FRT site"
     misc_feature    5733..5754
                     /note="attB4"
     gene            6677..6876
                     /note="sv40 terminator"
     misc_feature    6919..6924
                     /note="EcoRI"
     misc_feature    6997..7046
                     /note="3xP3 element"
     misc_feature    7256..8181
                     /note="DsRed"
     misc_feature    7086..7093
                     /note="TATA"
     misc_feature    2617..5720
                     /note="TEP1 5' region"
     misc_feature    5705..5720
                     /note="TEP1 5'UTR fragment"
     source          1..8929
                     /dnas_title="attBrf2_TEP1_GFP_Sv40dsRED"
ORIGIN
        1 GTGGCACTTT TCGGGGAAAT GTGCGCGGAA CCCCTATTTG TTTATTTTTC TAAATACATT
       61 CAAATATGTA TCCGCTCATG AGACAATAAC CCTGATAAAT GCTTCAATAA TATTGAAAAA
      121 GGAAGAGTAT GAGTATTCAA CATTTCCGTG TCGCCCTTAT TCCCTTTTTT GCGGCATTTT
      181 GCCTTCCTGT TTTTGCTCAC CCAGAAACGC TGGTGAAAGT AAAAGATGCT GAAGATCAGT
      241 TGGGTGCACG AGTGGGTTAC ATCGAACTGG ATCTCAACAG CGGTAAGATC CTTGAGAGTT
      301 TTCGCCCCGA AGAACGTTTT CCAATGATGA GCACTTTTAA AGTTCTGCTA TGTGGCGCGG
      361 TATTATCCCG TATTGACGCC GGGCAAGAGC AACTCGGTCG CCGCATACAC TATTCTCAGA
      421 ATGACTTGGT TGAGTACTCA CCAGTCACAG AAAAGCATCT TACGGATGGC ATGACAGTAA
      481 GAGAATTATG CAGTGCTGCC ATAACCATGA GTGATAACAC TGCGGCCAAC TTACTTCTGA
      541 CAACGATCGG AGGACCGAAG GAGCTAACCG CTTTTTTGCA CAACATGGGG GNATCATGTA
      601 ACTCGCCTTN GATCGTTGGG AACCGGAGCT GAATGAAGCC ATACCAAACG ACGAGCGTGA
      661 CACCACGATG CCTGTAGCAA TGGCAACAAC GTTGCGCAAA CTATTAACTG GCGAACTACT
      721 TACTCTAGCT TCCCGGCAAC AATTAATAGA CTGGATGGAG GCGGATAAAG TTGCAGGACC
      781 ACTTCTGCGC TCGGCCCTTC CGGCTGGCTG GTTTATTGCT GATAAATCTG GAGCCGGTGA
      841 GCGTGGGTCT CGCGGTATCA TTGCAGCACT GGGGCCAGAT GGTAAGCCCT CCCGTATCGT
      901 AGTTATCTAC ACGACGGGGA GTCAGGCAAC TATGGATGAA CGAAATAGAC AGATCGCTGA
      961 GATAGGTGCC TCACTGATTA AGCATTGGTA ACTGTCAGAC CAAGTTTACT CATATATACT
     1021 TTAGATTGAT TTAAAACTTC ATTTTTAATT TAAAAGGATC TAGGTGAAGA TCCTTTTTGA
     1081 TAATCTCATG ACCAAAATCC CTTAACGTGA GTTTTCGTTC CACTGAGCGT CAGACCCCGT
     1141 AGAAAAGATC AAAGGATCTT CTTGAGATCC TTTTTTTCTG CGCGTAATCT GCTGCTTGCA
     1201 AACAAAAAAA CCACCGCTAC CAGCGGTGGT TTGTTTGCCG GATCAAGAGC TACCAACTCT
     1261 TTTTCCGAAG GTAACTGGCT TCAGCAGAGC GCAGATACCA AATACTGTCC TTCTAGTGTA
     1321 GCCGTAGTTA GGCCACCACT TCAAGAACTC TGTAGCACCG CCTACATACC TCGCTCTGCT
     1381 AATCCTGTTA CCAGTGGCTG CTGCCAGTGG CGATAAGTCG TGTCTTACCG GGTTGGACTC
```

1441 AAGACGATAG TTACCGGATA AGGCGCAGCG GTCGGGCTGA ACGGGGGGTT CGTGCACACA
1501 GCCCAGCTTG GAGCGAACGA CCTACACCGA ACTGAGATAC CTACAGCGTG AGCTATGAGA
1561 AAGCGCCACG CTTCCCGAAG GGAGAAAGGC GGACAGGTAT CCGGTAAGCG GCAGGGTCGG
1621 AACAGGAGAG CGCACGAGGG AGCTTCCAGG GGGAAACGCC TGGTATCTTT ATAGTCCTGT
1681 CGGGTTTCGC CACCTCTGAC TTGAGCGTCG ATTTTTGTGA TGCTCGTCAG GGGGGCGGAG
1741 CCTATGGAAA AACGCCAGCA ACGCGGCCTT TTTACGGTTC CTGGCCTTTT GCTGGCCTTT
1801 TGCTCACATG TTCTTTCCTG CGTTATCCCC TGATTCTGTG GATAACCGTA TTACCGCCTT
1861 TGAGTGAGCT GATACCGCTC GCCGCAGCCG AACGACCGAG CGCAGCGAGT CAGTGAGCGA
1921 GGAAGCGGAA GAGCGCCCAA TACGCAAACC GCCTCTCCCC GCGCGTTGGC CGATTCATTA
1981 ATGCAGCTGG CACGACAGGT TTCCCGACTG GAAAGCGGGC AGTGAGCGCA ACGCAATTAA
2041 TGTGAGTTAG CTCACTCATT AGGCACCCCA GGCTTTACAC TTTATGCTTC CGGCTCGTAT
2101 GTTGTGTGGA ATTGTGAGCG GATAACAATT TCACACAGGA AACAGCTATG ACCATGATTA
2161 CGCCAAGCTC GAAATTAACC CTCACTAAAG GGAACAAAAG CTGGCTAGAA CTAGTGTCGA
2221 CATGCCCGCC GTGACCGTCG AGAACCCGCT GACGCTGCCC CGCGTATCCG CACCCGCCGA
2281 CGCCGTCGCA CGTCCCGTGC TCACCGTGAC CACCGCGCCC AGCGGTTTCG AGGGCGAGGG
2341 CTTCCCGGTG CGCCGCGCGT TCGCCGGGAT CAACTACCGC CACCTCGACC CGTTCATCAT
2401 GATGGACCAG ATGGGTGAGG TGGAGTACGC GCCCGGGGAG CCCAAGGGCA CGCCCTGGCA
2461 CCCGCACCGC GGCTTCGAGA CCGTGACCTA CATCGTCGAC ACTAGTGGat catcaacaag
2521 tttGTACAAA AAAGCAGGCT GGTACCGAAG TTCCTATTCT CTAGAAAGTA TAGGAACTTC
2581 GTCGACGGTA TCGATAAGCT tagggataac agggtaATGA CCATAGAACT TGCTGTATGG
2641 AGATTGAACA GTAAACTTGA AATGCCGTCG GGGGCCCCTT CGATTATCAG TCACAAATTT
2701 TTGCTTTTTT TCTAAAATTT TTGCTGACGG TGGGGGTGGG GGGGTGCAAA TGATTCACCA
2761 GCTTCGGGGC CCCAACGGCC ATCCTTCCGG GGCCTTTGTC GCTAGTACTC TGTCCGTACG
2821 GCATACTATT GATTGGTGAT CTACGGGGCC CCTATATCGG CGGGGCCCCA GGCGACCGCC
2881 TAGTCCGCCT ACCGTTCGAT TCGCCACTGC TTATGATTTT TGTTCTAGAC AACATTTTAA
2941 AATGAAATTT GTCATAAATT TGCGTTATGT TGTTAGCTTA CAAATAAGTT TTGATCATAT
3001 GTTTTTGATC ATATGTATAT GTTTTGATCA ATTTGTTCTT CTTGGGTTTG CCGAACAAGT
3061 TGCGTTGTCA TGCCGGCCTA TACAGGCTTT AGAGACTTAT TGGGTACCAC GCATCCGGAT
3121 ACACGGTCCT TTGCTACAAG AGGTAGTCCA TACCAGATAT GAAATCATGA AGGGAATGTT
3181 GTAAAGTCGC TGACGACTCA ATAACATGCT CTGAAATTGG GAAGAACCAA TGCCAAAGAT
3241 GTGCAAATGA AAATAAAATA AATTATGTCA AAGATCCTCC AACATGTCAA GAATGCCAAA
3301 GGATTGCATA TAAATTATAA GTTTGATGTT ACATTTGCTC AGAAAAATAT TCTCTGTAGA
3361 ATGAATAATG TAGGTACTTT ATTTTGGGAC AGTGATATTT ATCAATTTTG AAAAAAAAAA
3421 AAAGTTTTCT TGAAGCAAAT TTCTCATTTT TGTCATGCTA TGGTACTACA CATTCTTAAG
3481 CATATCACGA ACAATGTTTC AAAAAATAAA GCAGTACAAT AACTTCATTT TTCACGAAAA
3541 AAAAAACAAA TGTTAAATTG TAAGTAGTGG GCACTTTATT TTGCCGGCCA CTGTATATCG
3601 TATTTGTTAG CTGACAACAT AACGCAACTT TATGACAAGT TTCGTTTTAA AATGATGTCT
3661 AGAACGTAAA TTATTAGGTT TTCAATCCAC GAATTTTCAA ACTCCCATAC AAAAAGTTGG
3721 GCTTACTCGT GCAGGCGGTC GTAGAAATAC GTCGTTTTTT TGCGAGTAAG TTACAGTAGA
3781 AAACAATATC AAAATTCCAG GAGAATATTG TTAGTGGAGA TGGAAGCATT GATAAAAATT
3841 TCATGCATAT TAGACAAGCA GATAAAAAGG TATGAGCTTT TGAATCCTCA AAAATGTAAT
3901 GTCTCATACA AAATATACGG TCCTACCCAG GTAGGCGGTC ATAGATGTAA AGGGGTGTGG
3961 ATAGAGTTTG GAAGCACTGA AGATTACTAT GATGCATATC AAAGTAAGTA GTAGCTGGTC
4021 TCGCGATACA GTCGTCATCT CGTACGACTT AACAACATGC CCATCATGGG TTCAAGCACC
4081 GAATAGACCG TGCCCCCATA CGTAAGACAG ACTATTCTAC TATGTGTAAT AAGTAAGTCA
4141 CTGAAAACCG AGCAAAGTAA AGTAGTGGCA CAGGCACAAC GACAATCTTT GCGATAACGG
4201 TTGTTGTGCC AAAGAAGAAG AAGAAGAAGA AGAAGAAGAT ATCAAAGTAT GTCCATTTTG
4261 TCCCGCGTGC TCATTTTGCC CCGTGTTCCC TTACTCTAGC GGGAATCGTA TTTGGTTGAG
4321 CTATCTTGCA AATAAGGTCG ACCATGCCAG AGTAACATGG GTTCGTAGCG AAGTGATCAA
4381 CTGTTATCAA TGTTTTATAC GTTTCGCGTT TTCACCTTTG TGTATAATAA TTGGAATCAA
4441 ATCATATAAG ATGGTTTAGT ATGCACTTTC GAGCCAATTT AGTTTTTCTT TCATTCAACT
4501 AATTCTTGCA GGCTTGGAGT TTAAAGAAAA AGTACTTTAA GGTATAAAAA AACCCCAGCA
4561 CAACATAAAT AATTGTAAGT TGTAAGATAG TGATTTGATA AACATGAAGC AAAACTGAAA
4621 AACAATTGAA GCAATTTTTC CCAGATAATT ATTTATCTTT TTGTTATTTA TTGATTAGAA
4681 TTCAACAGAC CACTGCCTTC TTAAAGCGAA ACAAGGTCAC AAACATTTGA TACAGTCTCG
4741 TTTCAGTCAT CGTTTTTGCC GCGTTCAGTC AGAAACAAGT GTCCGTCAAA CTTCTTCATC
4801 TTCTTTTTTT ATTTCCAAAT CAATGGTTAA GGCATTTGCT GCATTCTATT TGCTAACCTT

4861 GTACATCATG TACGTATCTG TTCTGCATTG ATTAATTGCG TGGTCCATTT CATGTATATT
4921 AATCCTTGCC AAATAGTTAA CCGAATCAAA AACACCAATT GAGTCCTTTT CACTTGATGC
4981 GTTGATGGAT CAATTAACTT TTCGTACTGT TCGTTTCTAG AGAACTAACG GAGAACTAAT
5041 CATGACGTCT TCACACATTA CGGCTTACCA CAGGTAAATA TTTCACCCAG TACAATGCAA
5101 ACGACTCACT AAGCATTGTT TTAAAATCAT TGTTTTGAAC CGTATCACAA GATTATTTAA
5161 GAAATTTTCT TCAATAAAAA GCTACTCTTT AATTTCGTGT TTACAAATAC TTCAGCAGAA
5221 GAAATACAGA ATTGCAGAAA AATTAGCATG CTCGAAATAA CATCAAACTA CTAATACTTA
5281 TGTTTCTATA AGAGTTTACA ATGAGCAAAT AACAATATTA AACAAAACTA TGTGTGATAA
5341 AAGCAATACA GTAATCTATC AGTGAGCCAA AAAACTTACT ATGCAACAAG TTAATGCCAT
5401 ACTGTTTAAT GGCAACCTAG GTTAAAATTC AAACATTAAA TTACTTTTGT TAAAGGTAAA
5461 CTCTAGCAGG ATGTGCAGTT TTGCAAGCAT AAATACTGTT AACGCACTGC AATATTAGCC
5521 ACTGGAATCG GTATTTCTAT ACAAACCAAG GGGATTTATT CTTATCGCCC GGCGTTCATG
5581 CAGGTCGCTA TGTTTACAGT AACATCTGAG GGCAGGGAAT CCCCAATAAC AACAGCACAG
5641 CTTCACCGAT AGGGATGCAA ACTGACCGTG ATTCAGTTCA ATCGTAGACG GCATCCGAAG
5701 CAGACGCGAA ATCGCACAAC tagTCGAGCA CCCAACTTTT CTATACAAAG TTGGTACCGG
5761 GCCCCCCGCT AGCGTCGACG GTATCGATAA GCTTGATATC GAATTCGTTA ACAGATCCAC
5821 CGGTCGCCAC CATGGTGAGC AAGGGCGAGG AGCTGTTCAC CGGGGTGGTG CCCATCCTGG
5881 TCGAGCTGGA CGGCGACGTA AACGGCCACA AGTTCAGCGT GTCCGGCGAG GGCGAGGGCG
5941 ATGCCACCTA CGGCAAGCTG ACCCTGAAGT TCATCTGCAC CACCGGCAAG CTGCCCGTGC
6001 CCTGGCCCAC CCTCGTGACC ACCCTGACCT ACGGCGTGCA GTGCTTCAGC CGCTACCCCG
6061 ACCACATGAA GCAGCACGAC TTCTTCAAGT CCGCCATGCC CGAAGGCTAC GTCCAGGAGC
6121 GCACCATCTT CTTCAAGGAC GACGGCAACT ACAAGACCCG CGCCGAGGTG AAGTTCGAGG
6181 GCGACACCCT GGTGAACCGC ATCGAGCTGA AGGGCATCGA CTTCAAGGAG GACGGCAACA
6241 TCCTGGGGCA CAAGCTGGAG TACAACTACA ACAGCCACAA CGTCTATATC ATGGCCGacA
6301 AGCAGAAGAA CGGCATCAAG GTGAACTTCA AGATCCGCCA CAACATCGAG GaCGGCAGCG
6361 TGCAGCTCGC CGACCACTAC CAGCAGAACA CCCCCATCGG CGacGGCCCC GTGCTGCTGC
6421 CCGaCAACCA CTACCTGAGC ACCCAGTCCG CCCTGAGCAA agaCCCCAAC GAgaagCGCG
6481 ATCACATGGT CCTGCTGgaG TTCGTGACCG CCGCCGGgat CacTCTCGGC ATGGacGAgc
6541 TGTacaAGTa AAgCgGCCgc GGcTcGAGGG tACCTctAGa GCGGCCGCCA CCGCGGTGGA
6601 GCTCGAGACA ACTTTGTATA ATAAAGTTGG TACCGGGCCC CCCGCTAGCG TCGACGGTAT
6661 CGATAAGCTT GATATCGAAT TCTCTAGATC ATAATCAGCC ATACCACATT TGTAGAGGTT
6721 TTACTTGCTT TAAAAAACCT CCCACACCTC CCCCTGAACC TGAAACATAA AATGAATGCA
6781 ATTGTTGTTG TTAACTTGTT TATTGCAGCT TATAATGGTT ACAAATAAAG CAATAGCATC
6841 ACAAATTTCA CAAATAAAGC ATTTTTCTTC ACTGCATTCT AGTTGTGGTT TGTCCAAACT
6901 CATCAATGTA TCAAGGGCGA ATTCCTGCAG CCCACTTCCG GTATCTCGCG TTTGTTTGAT
6961 CGCACGGTTC CCACAATGGT TAATTCGAGC TCGCCCGGGG ATCTAATTCA ATTAGAGACT
7021 AATTCAATTA GAGCTAATTC AATTAGGATC CAAGCTTATC GATTTCGAAC CCTCGACCGC
7081 CGGAGTATAA ATAGAGGCGC TTCGTCTACG GAGCGACAAT TCAATTCAAA CAAGCAAAGT
7141 GAACACGTCG CTAAGCGAAA GCTAAGCAAA TAAACAAGCG CAGCTGAACA AGCTAAACAA
7201 TCGGGGTACC GCTAGAGTCG ACGGTACCGC GGGCCCGGGA TCCACCGGTC GCCACCATGG
7261 TGCGCTCCTC CAAGAACGTC ATCAAGGAGT TCATGCGCTT CAAGGTGCGC ATGGAGGGCA
7321 CCGTGAACGG CCACGAGTTC GAGATCGAGG GCGAGGGCGA GGGCCGCCCC TACGAGGGCC
7381 ACAACACCGT GAAGCTGAAG GTGACCAAGG GCGGCCCCCT GCCCTTCGCC TGGGACATCC
7441 TGTCCCCCCA GTTCCAGTAC GGCTCCAAGG TGTACGTGAA GCACCCCGCC GACATCCCCG
7501 ACTACAAGAA GCTGTCCTTC CCCGAGGGCT TCAAGTGGGA GCGCGTGATG AACTTCGAGG
7561 ACGGCGGCGT GGTGACCGTG ACCCAGGACT CCTCCCTGCA GGACGGCTGC TTCATCTACA
7621 AGGTGAAGTT CATCGGCGTG AACTTCCCCT CCGACGGCCC CGTAATGCAG AAGAAGACCA
7681 TGGGCTGGGA GGCCTCCACC GAGCGCCTGT ACCCCCGCGA CGGCGTGCTG AAGGGCGAGA
7741 TCCACAAGGC CCTGAAGCTG AAGGACGGCG GCCACTACCT GGTGGAGTTC AAGTCCATCT
7801 ACATGGCCAA GAAGCCCGTG CAGCTGCCCG GCTACTACTA CGTGGACTCC AAGCTGGACA
7861 TCACCTCCCA CAACGAGGAC TACACCATCG TGGACCAGTA CGAGCGCACC GAGGGCCGCC
7921 ACCACCTGTT CCTGTAGCGG CCGCGACTCT AGATCATAAT CAGCCATACC ACATTTGTAG
7981 AGGTTTTACT TGCTTTAAAA AACCTCCCAC ACCTCCCCCT GAACCTGAAA CATAAAATGA
8041 ATGCAATTGT TGTTGTTAAC TTGTTTATTG CAGCTTATAA TGGTTACAAA TAAAGCAATA
8101 GCATCACAAA TTTCACAAAT AAAGCATTTT TCTTCACTGC ATTCTAGTTG TGGTTTGTCC
8161 AAACTCATCA ATGTATCTTA AAGCTTATCG ATACGCGTAC GGCGCGCCTA GAGCGGCCGC
8221 CACCGCGGTG GAGCTCGAGT ACCCAGCTTT cttgtacaaa gtggttgatC GGTACGTACC

```
     8281 CAATTCGCCC TATAGTGAGT CGTATTACAA TTCACTGGCC GTCGTTTTAC AACGTCGTGA
     8341 CTGGGAAAAC CCTGGCGTTA CCCAACTTAA TCGCCTTGCA GCACATCCCC CTTTCGCCAG
     8401 CTGGCGTAAT AGCGAAGAGG CCCGCACCGA TCGCCCTTCC CAACAGTTGC GCAGCCTGAA
     8461 TGGCGAATGG AAATTGTAAG CGTTAATATT TTGTTAAAAT TCGCGTTAAA TTTTTGTTAA
     8521 ATCAGCTCAT TTTTTAACCA ATAGGCCGAA ATCGGCAAAA TCCCTTATAA ATCAAAAGAA
     8581 TAGACCGAGA TAGGGTTGAG TGTTGTTCCA GTTTGGAACA AGAGTCCACT ATTAAAGAAC
     8641 GTGGACTCCA ACGTCAAAGG GCGAAAAACC GTCTATCAGG GCGATGGCCC ACTACGTGAA
     8701 CCATCACCCT AATCAAGTTT TTTGGGGTCG AGGTGCCGTA AAGCACTAAA TCGGAACCCT
     8761 AAAGGGAGCC CCCGATTTAG AGCTTGACGG GGAAAGCCGG CGAACGTGGC GAGAAAGGAA
     8821 GGGAAGAAAG CGAAAGGAGC GGGCGCTAGG GCGCTGGCAA GTGTAGCGGT CACGCTGCGC
     8881 GTAACCACCA CACCCGCCGC GCTTAATGCG CCGCTACAGG GCGCGTCAG
//
```

pattB-RfB2-Vg-TEP1r SV40YFP (line *Vg-TEP1r*)

```
LOCUS       pattB-RfB2-VgTEP       10653 bp    DNA     circular       21-JUN-
     2016
FEATURES             Location/Qualifiers
     misc_feature    complement(2216..2500)
                     /note="attB"
     misc_feature    2563..4264
                     /note="Vitellogenin promoter"
     promoter        4372..8372
                     /note="ORF TEP1r"
     misc_feature    8472..8671
                     /note="sv40 terminator"
     gene            8980..9696
                     /note="EYFP (Clontech)"
     misc_feature    9703..9957
                     /note="SV40 term sequences"
     misc_feature    8720..8928
                     /note="3xP3-hsp70 promoter"
     source          1..10653
                     /dnas_title="pattB-RfB2-VgTEP1rSV40YFP"
ORIGIN
        1 GTGGCACTTT TCGGGGAAAT GTGCGCGGAA CCCCTATTTG TTTATTTTTC TAAATACATT
       61 CAAATATGTA TCCGCTCATG AGACAATAAC CCTGATAAAT GCTTCAATAA TATTGAAAAA
      121 GGAAGAGTAT GAGTATTCAA CATTTCCGTG TCGCCCTTAT TCCCTTTTTT GCGGCATTTT
      181 GCCTTCCTGT TTTTGCTCAC CCAGAAACGC TGGTGAAAGT AAAAGATGCT GAAGATCAGT
      241 TGGGTGCACG AGTGGGTTAC ATCGAACTGG ATCTCAACAG CGGTAAGATC CTTGAGAGTT
      301 TTCGCCCCGA AGAACGTTTT CCAATGATGA GCACTTTTAA AGTTCTGCTA TGTGGCGCGG
      361 TATTATCCCG TATTGACGCC GGGCAAGAGC AACTCGGTCG CCGCATACAC TATTCTCAGA
      421 ATGACTTGGT TGAGTACTCA CCAGTCACAG AAAAGCATCT TACGGATGGC ATGACAGTAA
      481 GAGAATTATG CAGTGCTGCC ATAACCATGA GTGATAACAC TGCGGCCAAC TTACTTCTGA
      541 CAACGATCGG AGGACCGAAG GAGCTAACCG CTTTTTTGCA CAACATGGGG GNATCATGTA
      601 ACTCGCCTTN GATCGTTGGG AACCGGAGCT GAATGAAGCC ATACCAAACG ACGAGCGTGA
      661 CACCACGATG CCTGTAGCAA TGGCAACAAC GTTGCGCAAA CTATTAACTG GCGAACTACT
      721 TACTCTAGCT TCCCGGCAAC AATTAATAGA CTGGATGGAG GCGGATAAAG TTGCAGGACC
      781 ACTTCTGCGC TCGGCCCTTC CGGCTGGCTG GTTTATTGCT GATAAATCTG GAGCCGGTGA
      841 GCGTGGGTCT CGCGGTATCA TTGCAGCACT GGGGCCAGAT GGTAAGCCCT CCCGTATCGT
      901 AGTTATCTAC ACGACGGGGA GTCAGGCAAC TATGGATGAA CGAAATAGAC AGATCGCTGA
      961 GATAGGTGCC TCACTGATTA AGCATTGGTA ACTGTCAGAC CAAGTTTACT CATATATACT
     1021 TTAGATTGAT TTAAAACTTC ATTTTTAATT TAAAAGGATC TAGGTGAAGA TCCTTTTTGA
     1081 TAATCTCATG ACCAAAATCC CTTAACGTGA GTTTTCGTTC CACTGAGCGT CAGACCCCGT
```

1141 AGAAAAGATC AAAGGATCTT CTTGAGATCC TTTTTTTCTG CGCGTAATCT GCTGCTTGCA
1201 AACAAAAAAA CCACCGCTAC CAGCGGTGGT TTGTTTGCCG GATCAAGAGC TACCAACTCT
1261 TTTTCCGAAG GTAACTGGCT TCAGCAGAGC GCAGATACCA AATACTGTCC TTCTAGTGTA
1321 GCCGTAGTTA GGCCACCACT TCAAGAACTC TGTAGCACCG CCTACATACC TCGCTCTGCT
1381 AATCCTGTTA CCAGTGGCTG CTGCCAGTGG CGATAAGTCG TGTCTTACCG GGTTGGACTC
1441 AAGACGATAG TTACCGGATA AGGCGCAGCG GTCGGGCTGA ACGGGGGGTT CGTGCACACA
1501 GCCCAGCTTG GAGCGAACGA CCTACACCGA ACTGAGATAC CTACAGCGTG AGCTATGAGA
1561 AAGCGCCACG CTTCCCGAAG GGAGAAAGGC GGACAGGTAT CCGGTAAGCG GCAGGGTCGG
1621 AACAGGAGAG CGCACGAGGG AGCTTCCAGG GGGAAACGCC TGGTATCTTT ATAGTCCTGT
1681 CGGGTTTCGC CACCTCTGAC TTGAGCGTCG ATTTTTGTGA TGCTCGTCAG GGGGGCGGAG
1741 CCTATGGAAA AACGCCAGCA ACGCGGCCTT TTTACGGTTC CTGGCCTTTT GCTGGCCTTT
1801 TGCTCACATG TTCTTTCCTG CGTTATCCCC TGATTCTGTG GATAACCGTA TTACCGCCTT
1861 TGAGTGAGCT GATACCGCTC GCCGCAGCCG AACGACCGAG CGCAGCGAGT CAGTGAGCGA
1921 GGAAGCGGAA GAGCGCCCAA TACGCAAACC GCCTCTCCCC GCGCGTTGGC CGATTCATTA
1981 ATGCAGCTGG CACGACAGGT TTCCCGACTG GAAAGCGGGC AGTGAGCGCA ACGCAATTAA
2041 TGTGAGTTAG CTCACTCATT AGGCACCCCA GGCTTTACAC TTTATGCTTC CGGCTCGTAT
2101 GTTGTGTGGA ATTGTGAGCG GATAACAATT TCACACAGGA AACAGCTATG ACCATGATTA
2161 CGCCAAGCTC GAAATTAACC CTCACTAAAG GGAACAAAAG CTGGCTAGAA CTAGTGTCGA
2221 CATGCCCGCC GTGACCGTCG AGAACCCGCT GACGCTGCCC CGCGTATCCG CACCCGCCGA
2281 CGCCGTCGCA CGTCCCGTGC TCACCGTGAC CACCGCGCCC AGCGGTTTCG AGGGCGAGGG
2341 CTTCCCGGTG CGCCGCGCGT TCGCCGGGAT CAACTACCGC CACCTCGACC CGTTCATCAT
2401 GATGGACCAG ATGGGTGAGG TGGAGTACGC GCCCGGGGAG CCCAAGGGCA CGCCCTGGCA
2461 CCCGCACCGC GGCTTCGAGA CCGTGACCTA CATCGTCGAC ACTAGTGgat catcaacaag
2521 tttGTACAAA AAAGCAGGCT GGTACCGGGC CCCCCGCTAG CGTCGAGTTC AACTCGACCA
2581 TAATAATTGA TCCGTCAATC CATATTGGTC CGCAATAATG AAAGTTGCAA GAGTACGACG
2641 GTATGAAAAG AGGTTCAGTA AGTTGTAAAC TAATAGTTTC TTCCCAACGT TCAAATGCTG
2701 GCAAATCTTT TCGCGGGCCG CACTTCGTCG ATCGCTAGTC TTAATGATAA TTTCTGAGAA
2761 AAAGGTGCTA CTGCATCTAC TATATTCTAC TGGATATAAA TGAAATAACA ACGTGAGACT
2821 CACCTACAAC ATGTAATTTA TTGATGGTTT AGTTTAACCA ACCTATGAAA TAATTTGATA
2881 TAGAAATTTG TAGTCGTTTT TCTATGAAGT AAAATTCTAA AATCAAACAT TAAACTGTTT
2941 TGTAGTACCC GGACTCATGG TATGGCTTCT ATTAGCCGTA AACAAAGATT TACAATTGAC
3001 TAAGGTTAGG TCCGACACTG TAGGAGCCAG CGCGTCCTTT CAATACATCA ACGGACCATC
3061 TCGTGTTGTT AAATACTTAT TATTATTATG GTTTGCTAAT TGATATGTTC CAAGACCGAT
3121 TTGGATTTCG AAATAAGTAT TCTCTGATTC ATTTTGGGAG CCGGTCTCGT GATACAGTCG
3181 TCAGCCCGTA CGACTTAACT ACATTCTCGT CATGGGTTCA AGCCCCAGAT GGACCGTGCC
3241 GCCATACGTA GAGTCAGTCC TATCCTGTTA TGGGGGGTAA TACATAAGAC ACTGAAAGCC
3301 AACCCCACAA GTGGTACAGA CAAGCCTTGa CCGACAATTG TTGTTGAGCC AAACAGAAGA
3361 AGAATCCATT TCGGGAAATG ATTTTATCAT TCAATCAAAC CAGTCAATCA TAAACATCAT
3421 AGTTTTAAAT ACTCAAAACT AGTTGAGATC TTTAAAACAC ATTATTTTAG TTTAATTAAA
3481 TGATCTGTTA GCTAGAAGGT AGATACGATA TTTTAGACAT TTCGTAATAG ATCGCAAATC
3541 TCTATTATGT TGGTAATTCA CTTCGTAAAA CTCTTAGGCA AAACTCTTAT TAGTAAACAA
3601 AATACTAATC AAACACTGAT AAACTAACGC GATTTATACA TTGGACAAAG AAGAGGCTGA
3661 TTTTAAAAAT ACTCGCTTTA AAATTTGCTT CATTCATCAA TGTATTGTAA AGCACATAAA
3721 GAACACAATC ATTGACTGAA AACAATTCCA CGTCTCAGCC AACTTCCAGG ATCAATGAAA
3781 TAGCAAGTTC CAAGTTCCAT TTCATTGATT ATGGTAACTA CTGATTATTT TCAATAACAA
3841 ATACTTCGAA GACTGCACAA TTCAAAAGTA TGCCAGAAAG AAAGGATTAC TATCAATTGT
3901 GGGTTAATCA AACTAAGACA GGTGGCAAAA ATGGAACCAT TGATTAAGGC AGCCACTGAC
3961 CGATTTCATT TAAAAAACAC ACTCTTGGAA GTTTCCACAC AATCTCACCT TTTGCCAATT
4021 TTAGCAAAGA CGTTGTGCTG CACTGATAAG AATCGAACTG TAAACATGTG GGCAGTAAAA
4081 ATTATTTCAT CGTTCAACAC GGCGGTCATT ACACTATTCG AAGCAGCTGA AAAGATTTGA
4141 TGATAGCAGG ACCGTGAGAT CAGCAAATTT GAGGTATAAA AGATGATCCT GCGACCACCA
4201 GAAGGCACAT TCGAGCTTTG GAGTGCATTC AAAGCATCCG GGCAACTGCG AACAACCGAA
4261 CCatCGATCT CGAGCACCCA ACTTTTCTAT ACAAAGTTGG TACCGGGCCC CCCGCTAGCG
4321 TCGACGGTAT CGATAAGCTT GATATCGAAT TCCTGCAGCC CGGGGGATCC AATGTGGCAG
4381 TTCATAAGGT CACGAATATT AACGGTGATA ATCTTCATAG GTGCTGCTCA TGGGCTACTG
4441 GTTGTGGGTC CGAAATTTAT ACGGGCCAAC CAGGAATACA CTCTGGTGAT CAGCAACTTT
4501 AACTCACAGC TAAGCAAAGT GGACCTGCTG TTAAAACTGG AAGGCGAAAC TGATAATGGT

4561 TTAAGCGTTC TGAACGTTAC CAAGATGGTT GACGTGCGAC GTAATATGAA CCGAATGATC
4621 AACTTCAATA TGCCTGAGGA TCTGACGGCT GGAAACTACA AAATAACTAT CGATGGACAG
4681 CGTGGCTTCA GCTTTCACAA GGAGGCAGAG CTGGTGTATC TCAGCAAATC GATATCGGGG
4741 CTAATACAGG TCGATAAGCC CGTATTTAAA CCTGGGGATA CGGTGAACTT CCGTGTGATC
4801 GTGCTGGACA CGGAGCTGAA ACCGCCGGCG AGGGTCAAGT CGGTTTATGT AACTATACGA
4861 GATCCTCAGC GCAATGTGAT TCGCAAATGG TCCACGGCAA AACTGTATGC CGGTGTGTTC
4921 GAGAGCGATC TACAGATAGC GCCTACTCCA ATGCTCGGGG TCTGGAATAT CTCGGTGGAG
4981 GTGGAAGGAG AAGAGCTTGT GTCAAAGACG TTTGAGGTGA AGGAGTACGT GTTGTCAACG
5041 TTCGACGTGC AGGTCATGCC ATCGGTGATT CCACTGGAAG AGCATCAAGC TGTGAATCTT
5101 ACAATCGAAG CGAACTATCA CTTTGGTAAG CCAGTGCAAG GAGTGGCCAA GGTGGAGCTG
5161 TACCTAGACG ACGATAAGCT AAAACTGAAA AAAGAGCTGA CTGTGTACGG AAAGGGCCAG
5221 GTAGAGTTGC GCTTTGACAA TTTTGCAATG GATGCGGATC AGCAGGATGT ACCAGTGAAG
5281 GTGTCGTTCG TCGAGCAGTA CACAAATCGT ACGGTGGTCA AACAGTCACA AATCACGGTA
5341 TATAGGTATG CGTACCGAGT AGAGTTGATA AAAGAGAGTC CACAGTTTCG TCCGGGACTC
5401 CCGTTCAAAT GTGCGCTTCA GTTTACACAC CATGATGGAA CACCGGCTAA AGGCATTAGC
5461 GGTAAGGTAG AGGTATCCGA TGTACGATTC GAAACGACAA CAACGAGTGA TAACGATGGA
5521 TTGATTAAGC TCGAGCTGCA ACCAAGTGAG GGTACTGAAC AACTCAGTAT TCACTTCAAT
5581 GCTGTTGATG GATTCTTTTT TTATGAAGAT GTGAATAAGG TAGAAACGGT TACGGATGCG
5641 TATATTAAAC TGGAGCTGAA ATCACCCATC AAACGGAACA AATTGATGCG TTTCATGGTG
5701 ACGTGCACGG AGCGCATGAC ATTCTTCGTG TACTATGTCA TGTCAAAGGG CAATATCATC
5761 GATGCAGGAT TCATGCGACC CAACAAGCAA CCGAAGTACC TGTTGCAGCT GAACGCAACA
5821 GAAAAGATGA TTCCGAGGGC GAAAATTCTC ATCGCTACCG TAGCGGGCCG CACGGTGGTG
5881 TACGACTTCG CAGACCTCGA TTTCCAAGAG CTTCGCAATA ATTTTGATTT AAGCATTGAC
5941 GAGCAAGAGA TCAAGCCGGG ACGACAAATC GAGCTGAGCA TGTCTGGACG CCCAGGAGCG
6001 TACGTTGGGC TGGCCGCGTA TGACAAAGCC TTGCTGCTTT TCAACAAGAA CCACGACCTG
6061 TTCTGGGAGG ACATTGGGCA GGTGTTTGAT GGGTTCCATG CAATCAATGA GAACGAGTTT
6121 GACATATTCC ACAGCTTGGG TCTGTTCGCC AGGACATTGG ACGATATCTT GTTCGACAGT
6181 GCAAATGAAA AGACGGGGCG TAATGCACTG CAGTCAGGCA AGCCGATCGG CAAGCTGGTG
6241 TCGTATCGGA CGAACTTCCA GGAATCGTGG TTGTGGAAAA ATGTTTCCAT CGGACGATCG
6301 GGAAGTCGCA AGTTGATCGA GGTAGTACCG GACACGACCA CCTCCTGGTA TCTGACGGGC
6361 TTCTCGATCG ATCCCGTGTA CGGGTTGGGT ATCATCAAGA AGCCAATCCA GTTCACAACA
6421 GTCCAGCCGT TCTACATCGT AGAGAACTTA CCATATTCAA TCAAACGAGG CGAAGCGGTT
6481 GTGTTGCAGT TTACGCTGTT CAACAACCTT GGAGCGGAGT ATATAGCCGA TGTGACGCTG
6541 TACAATGTGG CCAACCAGAC CGAGTTCGTC GGACGTCCAA ATACGGATCT CAGCTACACC
6601 AAATCCGTGA GCGTTCCTCC AAAAGTTGGT GTGCCAATCT CGTTCCTCAT CAAGGCCCGC
6661 AAGCTCGGCG AGATGGCGGT TCGTGTAAAG GCTTCGATAA TGCTGGGACA CGAAACGGAC
6721 GCCCTGGAAA AGGTAATACG GGTGATGCCT GAAAGTTTGG TGCAGCCGAG AATGGATACA
6781 CGCTTTTTCT GCTTCGACGA TCACAAAAAT CAAACGTTTC CGATCAACTT GGACATCAAC
6841 AAGAAGGCCG ACAGTGGATC GACAAAGATT GAGTTTCGAC TAAATCCCAA TTTGTTGACC
6901 ACGGTCATCA AGAACCTGGA CCATCTTCTC GGCGTTCCGA CGGGATGTGG TGAGCAGAAT
6961 ATGGTCAAAT TTGTTCCCAA CATTTTGGTA CTGGATTATT TGCATGCCAT CGGGTCGAAA
7021 GAACAGCATC TAATCGACAA AGCTACGAAT TTGTTGCGTC AAGGATATCA AAACCAGATG
7081 CGCTACCGTC AGACGGATGG TTCATTTGGT TTGTGGGAGA CTACTAATGG TAGCGTGTTT
7141 CTCACCGCGT TCGTTGGCAC ATCGATGCAA ACTGCAGTAA AATACATAAG CGATATTGAT
7201 GCAGCAATGG TGGAGAAGGC ATTGGATTGG TTAGCCTCGA AGCAGCATTT CTCGGGACGG
7261 TTTGACAAGG CCGGTGCAGA GTATCACAAA GAAATGCAAG GAGGGTTGCG CAATGGTGTG
7321 GCCCTCACAT CATATGTGTT GATGGCATTG CTGGAGAATG ACATTGCCAA AGCAAAGCAC
7381 GCAGAGGTGA TTCAAAAAGG AATGACCTAT CTGAGCAATC AGTTTGGATC CATCAACAAT
7441 GCATACGACC TATCGATAGC AACCTACGCG ATGATGTTGA ACGGACACAC CATGAAGGAG
7501 GAGGCACTCA ATAAGCTGAT TGATATGTCT TTCATTGATG CTGATAAAAA CGAACGGTTC
7561 TGGAACACAA CGAATCCAAT AGAAACCACC GCATATGCTC TGCTGTCGTT TGTGATGGCC
7621 GAGAAGTACA CAGACGGTAT ACCGGTCATG AATTGGTTGG TGAATCAACG TTACGTTACC
7681 GGTAGCTTTC CGAGCACGCA AGACACGTTT GTGGGGCTGA AAGCGCTGAC CAAAATGGCG
7741 GAAAAGATAT CTCCGTCCCG AAACGACTAC ACCGTTCAAC TGAAGTACAA GAAGAGTGCA
7801 AAATACTTCA AAATAAACTC GGAGCAAATT GATGTGGAAA ACTTCGTGGA TATACCGGAG
7861 GACACAAAAA AGCTCGAGAT CAATGTGGGG GGCATTGGAT TTGGGTTGTT AGAGGTGGTT
7921 TATCAATTTA ATTTGAATCT CGTCAACTTT GAGAATAGAT TCCAACTAGA CCTGGAGAAA

```
     7981 CAGAACACAG GCTCTGACTA CGAGCTGAGG CTGAAGGTCT GTGCCAGCTA CATACCCCAG
     8041 CTGACCGACA GACGATCGAA CATGGCACTG ATTGAGGTAA CCTTACCGAG CGGTTACGTG
     8101 GTTGATCGCA ATCCGATCAG CGAGCAGACG AAGGTGAATC CGATTCAGAA AACTGAAATC
     8161 CGTTACGGTG GCACTTCAGT CGTTTTATAC TACGACAATA TGGGCAGCGA GCGTAACTGT
     8221 TTCACCCTGA CCGCGTACAG ACGCTTTAAG GTCGCATTGA AGCGTCCAGC GTATGTGGTT
     8281 GTGTATGATT ATTATAATAC AAATCTGAAC GCCATCAAAG TGTACGAAGT GGACAAGCAG
     8341 AATTTGTGCG AAATCTGTGA CGAAGAAGAC TGtcctGCAG AGTGCAAGAA ATAGTCctcG
     8401 AGACAACTTT GTATAATAAA GTTGGTACCG GGCCCCCCGC TAGCGTCGAC GGTATCGATA
     8461 AGCTTGATAT CGAATTCTCT AGATCATAAT CAGCCATACC ACATTTGTAG AGGTTTTACT
     8521 TGCTTTAAAA AACCTCCCAC ACCTCCCCCT GAACCTGAAA CATAAAATGA ATGCAATTGT
     8581 TGTTGTTAAC TTGTTTATTG CAGCTTATAA TGGTTACAAA TAAAGCAATA GCATCACAAA
     8641 TTTCACAAAT AAAGCATTTT TCTTCACTGC ATTCTAGTTG TGGTTTGTCC AAACTCATCA
     8701 ATGTATCAAG GGCGAATTCG GGGATCTAAT TCAATTAGAG ACTAATTCAA TTAGAGCTAA
     8761 TTCAATTAGG ATCCAAGCTT ATCGATTTCG AACCCTCGAC CGCCGGAGTA TAAATAGAGG
     8821 CGCTTCGTCT ACGGAGCGAC AATTCAATTC AAACAAGCAA AGTGAACACG TCGCTAAGCG
     8881 AAAGCTAAGC AAATAAACAA GCGCAGCTGA ACAAGCTAAA CAATCGGGGT ACCGCTAGAG
     8941 TCGACGGTAC CGCGGGCCCG GGATCCACCG GTCGCCACCA TGGTGAGCAA GGGCGAGGAG
     9001 CTGTTCACCG GGGTGGTGCC CATCCTGGTC GAGCTGGACG GCGACGTAAA CGGCCACAAG
     9061 TTCAGCGTGT CCGGCGAGGG CGAGGGCGAT GCCACCTACG GCAAGCTGAC CCTGAAGTTC
     9121 ATCTGCACCA CCGGCAAGCT GCCCGTGCCC TGGCCCACCC TCGTGACCAC CTTCGGCTAC
     9181 GGCCTGCAGT GCTTCGCCCG CTACCCCGAC CACATGAAGC AGCACGACTT CTTCAAGTCC
     9241 GCCATGCCCG AAGGCTACGT CCAGGAGCGC ACCATCTTCT TCAAGGACGA CGGCAACTAC
     9301 AAGACCCGCG CCGAGGTGAA GTTCGAGGGC GACACCCTGG TGAACCGCAT CGAGCTGAAG
     9361 GGCATCGACT TCAAGGAGGA CGGCAACATC CTGGGGCACA AGCTGGAGTA CAACTACAAC
     9421 AGCCACAACG TCTATATCAT GGCCGACAAG CAGAAGAACG GCATCAAGGT GAACTTCAAG
     9481 ATCCGCCACA ACATCGAGGA CGGCAGCGTG CAGCTCGCCG ACCACTACCA GCAGAACACC
     9541 CCCATCGGCG ACGGCCCCGT GCTGCTGCCC GACAACCACT ACCTGAGCTA CCAGTCCGCC
     9601 CTGAGCAAAG ACCCCAACGA GAAGCGCGAT CACATGGTCC TGCTGGAGTT CGTGACCGCC
     9661 GCCGGGATCA CTCTCGGCAT GGACGAGCTG TACAAGTAAA GCGGCCGCGA CTCTAGATCA
     9721 TAATCAGCCA TACCACATTT GTAGAGGTTT TACTTGCTTT AAAAAACCTC CCACACCTCC
     9781 CCCTGAACCT GAAACATAAA ATGAATGCAA TTGTTGTTGT TAACTTGTTT ATTGCAGCTT
     9841 ATAATGGTTA CAAATAAAGC AATAGCATCA CAAATTTCAC AAATAAAGCA TTTTTTTCAC
     9901 TGCATTCTAG TTGTGGTTTG TCCAAACTCA TCAATGTATC TTAAAGCTTA TCGATACctc
     9961 gagTACCCAG CTTTcttgta caaagtggtt gatCGGTACG TACCCAATTC GCCCTATAGT
    10021 GAGTCGTATT ACAATTCACT GGCCGTCGTT TTACAACGTC GTGACTGGGA AAACCCTGGC
    10081 GTTACCCAAC TTAATCGCCT TGCAGCACAT CCCCCTTTCG CCAGCTGGCG TAATAGCGAA
    10141 GAGGCCCGCA CCGATCGCCC TTCCCAACAG TTGCGCAGCC TGAATGGCGA ATGGAAATTG
    10201 TAAGCGTTAA TATTTTGTTA AAATTCGCGT TAAATTTTTG TTAAATCAGC TCATTTTTTA
    10261 ACCAATAGGC CGAAATCGGC AAAATCCCTT ATAAATCAAA AGAATAGACC GAGATAGGGT
    10321 TGAGTGTTGT TCCAGTTTGG AACAAGAGTC CACTATTAAA GAACGTGGAC TCCAACGTCA
    10381 AAGGGCGAAA AACCGTCTAT CAGGGCGATG GCCCACTACG TGAACCATCA CCCTAATCAA
    10441 GTTTTTTGGG GTCGAGGTGC CGTAAAGCAC TAAATCGGAA CCCTAAAGGG AGCCCCCGAT
    10501 TTAGAGCTTG ACGGGGAAAG CCGGCGAACG TGGCGAGAAA GGAAGGGAAG AAAGCGAAAG
    10561 GAGCGGGCGC TAGGGCGCTG GCAAGTGTAG CGGTCACGCT GCGCGTAACC ACCACACCCG
    10621 CCGCGCTTAA TGCGCCGCTA CAGGGCGCGT CAG
//
```

Promoter sequences:

Lp promoter

ATCCagcttgcggggaagacacattcgagatacgctaagtgattgagcgattacgatctagcaaaacatacgttcagct
gtgagaataatcatccatcttcctgcaatgagcagttcattcccgattgagggattttattccccgggggccttttcaaacggctt
aatataagcaattaatagtattttttctttcaggttagtttactgtaatggtgtaattgtcatcttacacctccgtctgataagagatta
cgaagctcagtatgatgaaataaataagataaatttatttaaaaaagaacaattgctatgagagtgaaatacaacagtggc
gttcacaatattcgaaaaacaataaaattaaaaaaaaaacaagaaaacattcacaaacatatcaatctgctttcatcgac
accgaactgctagcctccccagtctaaccgcggtggggacgtttaattgcctttgttctcgcacccggtcaaacatacacttc
ggaccttgctccgaaccccactgtgatccctagctcgtcatcatcattgccggcatcatgctaagcgtgcattattttcacaact
tagcgtaatgctagcgtgcgctagcaacaaactcgcccgcagactcgtcacagcaccggtacgatcgatcgtttaccgttc
cctttcccgatcgggttggctgcgatatccgtgtccgggtagaaaacttccccttttacacacacacactcacatacacacag
agctgaatagcaacttaccttatctgttcgtcatcgctcgggcggatctggacgaatcttcgcaccgataaccatgtggatcta
cgacctccgcttggctgtctctctgctcatgtgtatgtctgtgtgtgtgtgtatgtgagcttcttccctcaaatccctcgatctcgctgt
gscaacaatcaaacgtgcaagtgcaaacattgcaccccattgattatacacccaacaccaaccaattccccttgcggagg
catctctgtgctcggcagcatgtttaccgcagatctacagagaacttcaattgaggtcctttccacccccagccctcaaccgg
caatccggcagccactggatcatcagcgaaagagagagagagcagagcagaacagaggtgaccaactgtggtatcg
cttcccgcgcgccggtgtgttggtgtccatttcggtgatcgcgatcccggccgcttccagcaccgtccaccgatcagtcacaa
aaacgctctccaaaccccttatcagcaccgttcgctgatgtgaaccccccgttcaaaccccaaatgcagtgtttgtattgctgtg
tgtatgtgtacgtgcgtgtgtgtggaaattctataaaagtaggcacccgtggccgggatccgttattcccgtcctgaggcccg
cccgggatcgctggtgacgacagacgagccgctgtgtgacgtacgtagtgcccgatcggtaaagagtgaaccgtcttctct
gcagtgtaggagagaacggtttcatctttttcgcccacacccccccggttacattccatgttgaactgtaaggtctagtgaaca
tttcctgagtgtggaaagtgtggtttagtgcgtgagagtgcacggacacgacacT**ATG**

Vg promoter

ATCCcgctagcgtcgagttcaactcgaccataataattgatccgtcaatccatattggtccgcaataatgaaagttgcaag
agtacgacggtatgaaaagaggttcagtaagttgtaaactaatagtttcttcccaacgttcaaatgctggcaaatcttttcgcg
ggccgcacttcgtcgatcgctagtcttaatgataatttctgagaaaaaggtgctactgcatctactatattctactggatataaat
gaaataacaacgtgagactcacctacaacatgtaatttattgatggtttagtttaaccaacctatgaaataatttgatatagaa
atttgtagtcgtttttctatgaagtaaaattctaaaatcaaacattaaactgttttgtagtacccggactcatggtatggcttctatta
gccgtaaacaaagatttacaattgactaaggttaggtccgacactgtaggagccagcgcgtcctttcaatacatcaacgga
ccatctcgtgttgttaaatacttattattattatggtttgctaattgatatgttccaagaccgatttggatttcgaaataagtattctctg
attcattttgggagccggtctcgtgatacagtcgtcagcccgtacgacttaactacattctcgtcatgggttcaagccccagat
ggaccgtgccgccatacgtagagtcagtcctatcctgttatgggggtaatacataagacactgaaagccaaccccacaa
gtggtacagacaagccttgaccgacaattgttgttgagccaaacagaagaagaatccatttcgggaaatgattttatcattc
aatcaaaccagtcaatcataaacatcatagttttaaatactcaaaactagttgagatctttaaaacacattattttagtttaatta
aatgatctgttagctagaaggtagatacgatattttagacatttcgtaatagatcgcaaatctctattatgttggtaattcacttcgt
aaaactcttaggcaaaactcttattagtaaacaaaatactaatcaaacactgataaactaacgcgatttatacattggacaa
agaagaggctgattttaaaaatactcgctttaaaatttgcttcattcatcaatgtattgtaaagcacataaagaacacaatcatt
gactgaaaacaattccacgtctcagccaacttccaggatcaatgaaatagcaagttccaagttccatttcattgattatggta
actactgattattttcaataacaaatacttcgaagactgcacaattcaaaagtatgccagaaagaaaggattactatcaattg
tgggttaatcaaactaagacaggtggcaaaaatggaaccattgattaaggcagccactgaccgatttcatttaaaaaaca
cactcttggaagtttccacacaatctcaccttttgccaattttagcaaagacgttgtgctgcactgataagaatcgaactgtaa
acatgtgggcagtaaaaattatttcatcgttcaacacggcggtcattacactattcgaagcagctgaaaagatttgatgatag
caggaccgtgagatcagcaaatttgaggtataaaagatgatcctgcgaccaccagaaggcacattcgagctttggagtgc
attcaaagcatccgg

TEP1 minimal promoter

tccggatgtgcagttttgcaagcataaatactgttaacgcactgcaatattagccactggaatcggtatttctatacaaaccaa ggggatttattcttatcgcccggcgttcatgcaggtcgctatgtttacagtaacatctgagggcagggaatccccaataacaa cagcacagcttcaccgatagggatgcaaactgaccgtgattcagttcaatcgtagacggcatccgaagcagacgcgaaa tcgcacaacgtccaatccaacc

TALE sequences:

N terminal domain (start codon is in bold, *Bsa*I overhangs are capitalized):

T**ATG**gccaagaagaagaggaaggtgcaggtggatctacgcacgctcggctacagccagcagcaacaggagaaga tcaaaccgaaggttcgttcgacagtggcgcagcaccacgaggcactggtcggccatgggtttacacacgcgcacatcgtt gcgctcagccaacacccggcagcgttagggaccgtcgctgtcaagtatcaggacatgatcgcagcgttgccagaggcg acacacgaagcgatcgttggcgtcggcaaacagtggtccggcgcacgcgctctggaggccttgctcacggtggcggga gagttgagaggtccaccgttacagttggacacaggccaacttctcaagattgcaaagcgtggcggcgtgaccgcagtgg aggcagtgcatgcatggcgcaatgcactgacgggtgcccccct

C terminal domain (*Bsa*I overhangs are capitalized):

AGCAttgttgcccagttatctcgccctgatccggcgttggccgcgttgaccaacgaccacctcgtcgccttggcctgcctcg gcggacgtcctgcgctggatgcagtgaaaaagggattgccgcacgcgccggccttgatcaaaagaaccaatcgccgtat tcccgaacgcacatcccatcgcgttgcc

Repeat domain: (Capital letters correspond to *Bsa*I overhangs, nnnnnn correspond to two codons encoding the repeat-variable diresidues (RVD) that determine specificity for DNA bases)

GAACccccggagcaggtggtggccatcgccagcnnnnnnggcggtaagcaggcgctggagacggtgcagcggct gttgccggtgctgtgccaggcccatggcctgacACCGgagcaggtggtggccatcgccagcnnnnnnggtggcaag caggcgctggagacggtgcagcggctgttgccggtgctgtgccaggcccatggcctCACCccggagcaggtggtggc catcgccagcnnnnnnggcggtaagcaggcgctggagacggtgcagcggctgttgccggtgctgtgccaggcccatgg cctgACTCcggagcaggtggtggccatcgccagcnnnnnnggtggcaagcaggcgctggagacggtgcagcggct gttgccggtgctgtgccaggcCCATggcctgaccccggagcaggtggtggccatcgccagcnnnnnnggtggcaag caggcgcTGGAgacggtgcagcggctgttgccggtgctgtgccaggcgcatggccTTACcccggagcaggtggtg gccatcgccagcnnnnnnggtggcaagcaggcgctggagacggtgcagcggctgttgccggtgctgtgccaggcccat ggcCTGAccccggagcaggtggtggccatcgccagcnnnnnnggcggtaagcaggcgctggagacggtgcagcg gctgttgccggtgctgtgccaggcccatggcctgacACCGgagcaggtggtggccatcgccagcnnnnnnggtggca agcaggcgctggagacggtgcagcggctgttgccggtgctgtgccaggcccatggcctCACCccggagcaggtggtg gccatcgccagcnnnnnnggcggtaagcaggcgctggagacggtgcagcggctgttgccggtgctgtgccaggcccat ggcctgACTCcggagcaggtggtggccatcgccagcnnnnnnggtggcaagcaggcgctggagacggtgcagcg gctgttgccggtgctgtgccaggcCCATggcctgaccccggagcaggtggtggccatcgccagcnnnnnnggtggca agcaggcgcTGGAgacggtgcagcggctgttgccggtgctgtgccaggcgcatggccTTACcccggagcaggtgg tggccatcgccagcnnnnnnggtggcaagcaggcgctggagacggtgcagcggctgttgccggtgctgtgccaggccc atggcCTGAccccggagcaggtggtggccatcgccagcnnnnnnggcggtaagcaggcgctggagacggtgcag

cggctgttgccggtgctgtgccaggcccatggcctgacACCGgagcaggtggtggccatcgccagcnnnnnnggtgg
caagcaggcgctggagacggtgcagcggctgttgccggtgctgtgccaggcccatggcctCACCccggagcaggtg
gtggccatcgccagcnnnnnnggcggtaagcaggcgctggagacggtgcagcggctgttgccggtgctgtgccaggcc
catggcctgACTCcggagcaggtggtggccatcgccagcnnnnnnggtggcaagcaggcgctggagacggtgcag
cggctgttgccggtgctgtgccaggcCCATggcctgaccccggagcaggtggtggccatcgccagcnnnnnnggtgg
caagcaggcgc

Vp16 activation domain:

GGTGttcatctcgacggtgaggacgtggctatggcacacgccgatgcgctggatgattttgacctggatatgctgggtgat
ggcgacagcccaggacccggtttcacgccacacgactcggctccgtacggagcgctggacaccgctgacttcgagttcg
agcagatgttcaccgacgccctgggaatcgacgaatacggtggct
